# Solvent-Free and Efficient Synthesis of Silatranes *via* an Organocatalytic Protocol under Mild Conditions

**DOI:** 10.1021/acssuschemeng.3c07293

**Published:** 2024-01-10

**Authors:** Myong Joon Oh, Ireneusz Kownacki, Maciej Kubicki

**Affiliations:** † Faculty of Chemistry, Adam Mickiewicz University in Poznan, Uniwersytetu Poznanskiego 8, 61-614 Poznan, Poland; ‡ Center for Advanced Technology, Adam Mickiewicz University in Poznan, Uniwersytetu Poznanskiego 10, 61-614 Poznan, Poland

**Keywords:** silatranes, silane coupling agents, organocatalysis, amidines, organic bases

## Abstract

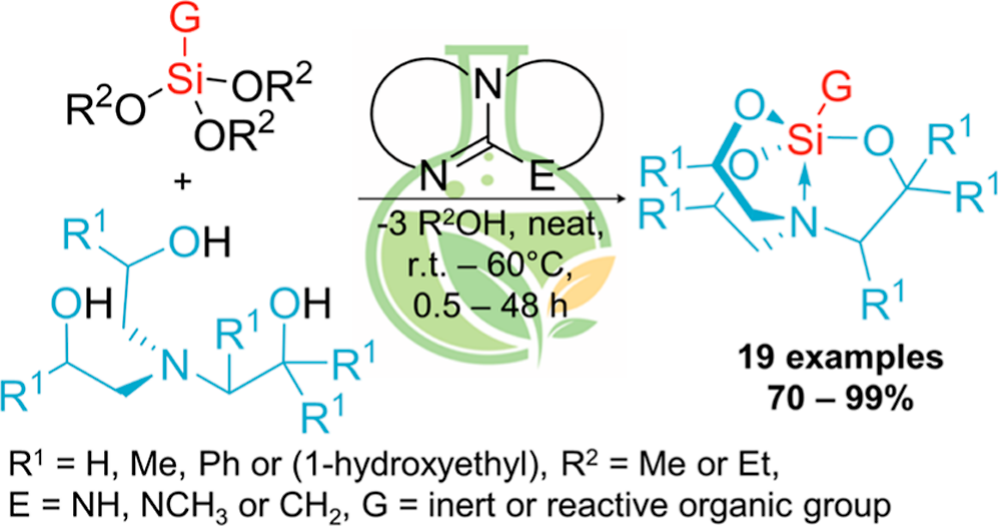

The organocatalytic approach to the formation of silatranyl cages permitted the design of a solvent-free and efficient protocol for the preparation of various organosilatranes. We discovered that amidine derivatives efficiently catalyze the conversion of trialkoxysilanes into organosilatranes, and their catalytic activity is related to the p*K*_BH+_ values. NMR studies of equimolar reactions of 1,8-diazabicyclo[5.4.0]undec-7-ene (DBU) and 1,5,7-triazabicyclo[4.4.0]dec-5-ene (TBD) with selected substrates allowed proposing a reliable scheme for the transesterification process and silatranyl cage formation. In addition, green chemistry metrics for the scaled-up synthesis of vinylsilatrane (**3k**) were appointed. Finally, a scheme for the industrial production of silatrane derivatives with DBU and solvent regeneration was proposed, supported by a catalyst recycling experiment.

## Abstract 2

A new methodology for the synthesis of silatranes was developed and demonstrated to obtain a library of silatrane derivatives, fulfilling most criteria of green chemistry.

## Introduction

Silatranes make up a well-known, specific subclass of trialkoxysilanes with the *C*_3*v*_ symmetry of the trialkoxysilyl moiety. In a silatrane molecule, all three alkoxyalkyl arms are connected to a nitrogen atom, which is in turn transannularly coordinated to the silicon center. Thus, a tricyclic, rigid cage is created.^1,2^ Thanks to the hypervalence of the central silicon atom and a significant steric hindrance, the silatrane cage system does not readily undergo nucleophilic substitution. Accordingly, hydrolysis and transesterification occur exceptionally slowly at neutral pH and ambient temperature due to a strong chelating effect.^3^ Consequently, the silatranes exhibit high air stability, a long shelf life, incredible chemical resistance, and significantly greater thermal stability than their alkoxysilyl counterparts.^4^ The above-mentioned properties are very attractive, particularly in terms of obtaining alkoxysilyl-functionalized polymers. Moreover, due to the high dipole moment and the strong electron-donating effect of the silatrane skeleton architecture, such derivatives not only are an interesting object of theoretical research^2,5−8^ but have also been investigated as unique organosilicon functional reagents,^9−14^ precursors of advanced polymer materials,^15^ silicon-based catalysts,^16^ or optical probes.^17^ For the above-mentioned reasons, silatrane derivatives have also been extensively studied in the context of their biological activity.^18−28^

Apart from that, another potential application of silatrane derivatives is surface modification. Several recently published reports have proved that silatrane derivatives provide higher functional group density on the treated surface, better surface coverage and molecular uniformity, stability within a wider pH range, and better reproducibility of the created film on inorganic oxide particles^29,30^ than conventional silane coupling agents (SCAs). The beneficial effects of the silatranyl group arise from its suppressed tendency to self-condensation, in contrast to a common trialkoxysilyl moiety.^31^ For this reason, they seem to be very attractive alternatives of conventional SCAs in metal oxide surface treatment.^29,30,32−37^

**Figure 1:**
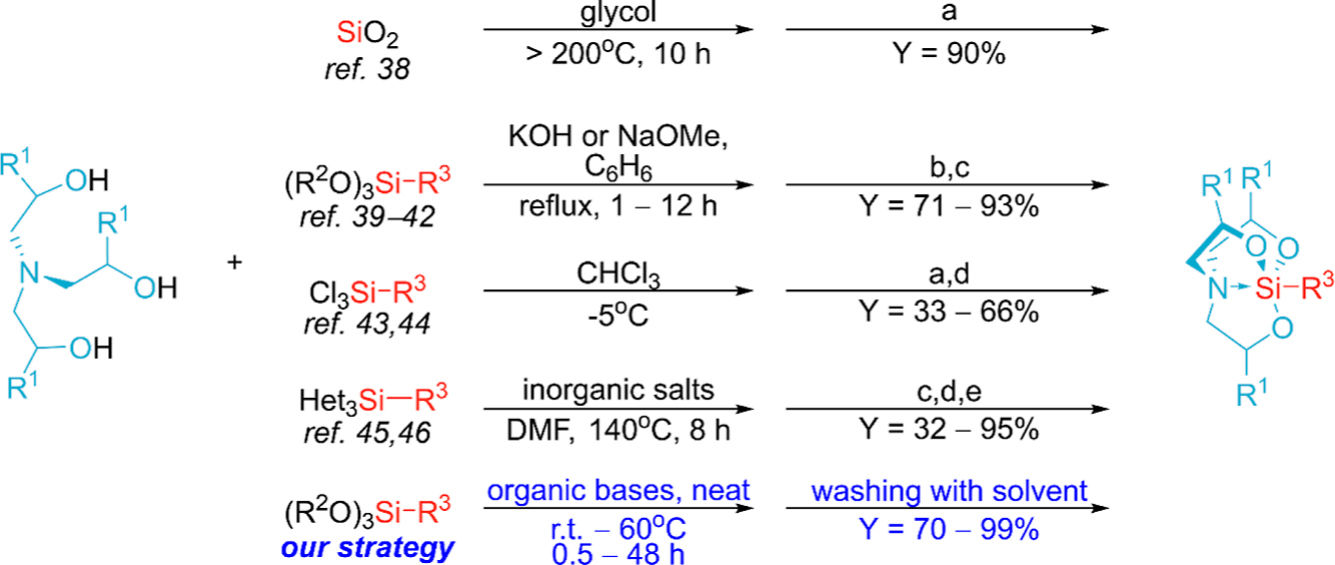
Overview of the hitherto known methods leading to silatrane derivatives by silatranyl cage formation *vs* our strategy. R^1^ = H and Me. R^2^ = Me and Et. R^3^ is the organic functional group. Het = 2-thienyl, 2-furyl, 2-(4,5-dihydrofuryl), and 2-(5,6-dihydro-4*H*-pyranyl). Workup process: a—vacuum drying at >120 °C, b—vacuum distillation, c—recrystallization, d—extraction, and e—column chromatography.

However, despite many advantages, silatranes, unfortunately, are not as widely used as could be expected. The reason is the cumbersome methodology for their synthesis and purification. Many methods of obtaining silatranes with the use of triethanolamine (TEOA) or its derivatives have been described in the literature, as shown in Figure 1.^2,38−46^

In some proposed procedures, the corresponding boratranes^46−49^ have also been used. However, the known protocols leading to the expected silatranes usually require long-term heating of the substrates in high-boiling-point solvents and/or the need to use strong inorganic bases as catalysts (see Figure 1). Therefore, silicon precursors equipped with thermally or chemically unstable functional groups cannot be employed in the protocols mentioned above. Moreover, conducting the processes with the use of inorganic catalysts/salts^39−42,45,46^ requires their separation from the products obtained, as in the long time of storage they may lead to undesirable transformation or even decomposition of silatranyl cages as well as organofunctional groups (exemplary in **3a**, **3d**, **3e**, **3g**, **3i**, **3j**, **3n**, and **3o–3s**), which is a serious problem and limiting their applicability to a small number of derivatives. Purification of crude silatrane products is another issue that must be overcome. Vacuum-assisted techniques, column chromatography, or recrystallization are often necessary, as shown in Figure 1. From the point of view of the large-scale production of silatranes, the use of catalytic systems or procedures mentioned above is very cumbersome to perform and economically unfavorable.

Therefore, our current studies are focused on developing a novel method that will enable easy synthesis and isolation without the need to use additional purification methods. Thus, the concept of research to solve this problem was based on the possibility of using soluble and easily removable organic bases (organocatalysts) instead of inorganic ones^39−42,45,46^ (Figure 1) as promoters of the selective formation of silatranyl cages in a solvent-free process. Additionally, the transformation of reagents to desired products should take place in very mild conditions, ensuring low energy consumption, which will also be in line with the guidelines of green chemistry.^50^

## Results and Discussion

In the initial phase of the study, tests of the catalytic activity of commonly used organic bases were carried out on the model reagent system shown in Table 1.

**Table 1:**
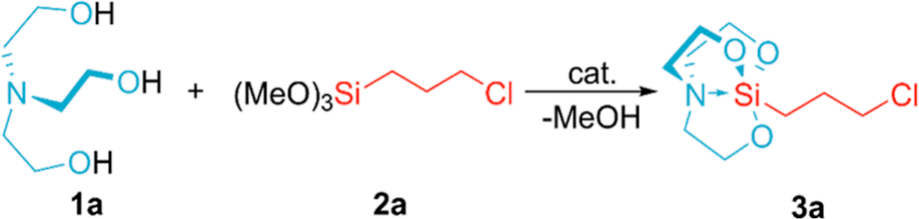
Screening of Organic Base Catalytic Activity in the Model Reaction^a^

entry	catalyst	*T* [°C]	reaction time [h]	yield of **3a** [%]^b^
1		r.t.	1	<1
2	NEt_3_	r.t.	1	<1
3	DIPEA	r.t.	1	<1
4	DABCO	r.t.	1	<1
5	DMAP	r.t.	1	<1
6	DBU	r.t.	1	>99

a Reaction conditions: [**1a**]/[**2a**]/[cat.] = 1:1.03:0.01, neat.

b Yield of the isolated compound.

**Figure 2:**
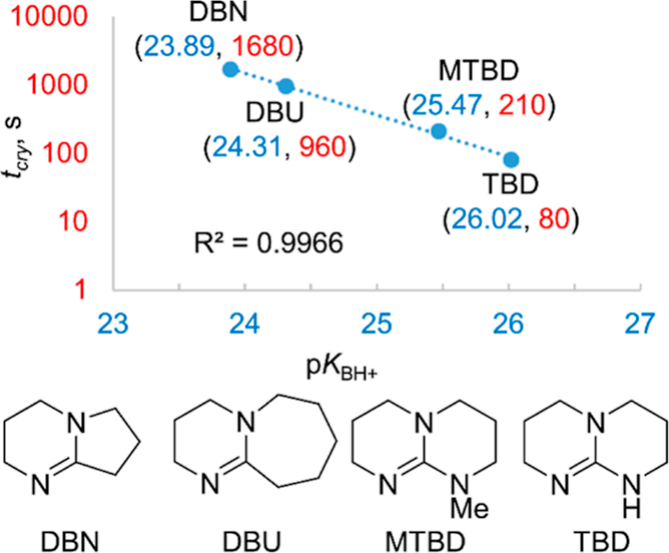
Dependence of the time needed to start the crystal formation (*t*_cry_) on the value of p*K*_BH+_ in MeCN (retrieved from the literature^51^). Average *t*_cry_ values from three measurements are shown for each catalyst. Reaction conditions: [**1a**]/[**2a**]/[cat.] = 1:1:0.01, r.t., neat.

The compiled results clearly indicate that under the given reaction conditions, the process does not occur without a catalyst or in the presence of typical bases such as trialkyl amines or a pyridine derivative. Only 1,8-diazabicyclo[5.4.0]undec-7-ene (DBU) was efficient in this process and permitted attaining a quantitative yield of the corresponding silatrane (**3a**). We observed that the introduction of DBU into the two-phase reagent system caused its homogenization after a few minutes, and then, precipitation of a white crystalline product was observed during the course of the reaction. The addition of a small excess of silane allowed complete conversion of the highly polar TEOA (**1a**) and easy isolation of the resulting silatrane through washing of the polar precipitate with three small portions of hexane (the initial silicon reagent and catalyst were removed). At this point, it should be emphasized that the mentioned compounds present in the collected solution can be recycled in the process after evaporation of volatile ingredients, which is also in accordance with the principles of green chemistry. As a result, this straightforward purification protocol allowed obtaining a spectroscopically pure derivative **3a** (see the Supporting Information). Considering that among the tested organic bases, only DBU turned out to be an active organocatalyst of the studied reaction, we decided to perform a screening of the efficiencies of other bases with a bicyclic structure based on the amidine core, such as 1,5,7-triazabicyclo[4.4.0]dec-5-ene (TBD), 7-methyl-1,5,7-triazabicyclo[4.4.0]dec-5-ene (MTBD), and 1,5-diazabicyclo[4.3.0]non-5-ene (DBN). Due to the heterogeneity of the model reaction mixture (TEOA + **2a**) both before (liquid/liquid) and after (solid/liquid) the reaction, monitoring the reaction progress with real-time spectroscopic techniques did not provide repeatable results. Hence, we observed the time at which precipitation of product **3a** crystals from the reaction mixture began (*t*_cry_), as an alternative measure of catalytic activity. The obtained *t*_cry_ values exhibited an excellent correlation with the basicity (p*K*_BH+_) of the amidines, as presented in Figure 2. The process of product crystal formation takes place in the shortest time when the TBD catalyst is used; thus, it can be assumed that the catalytic activity of this amine is the highest. Unlike the aforementioned base, DBN was characterized by the lowest activity. On the other hand, it can be assumed that DBU is a moderately active catalyst.

Taking into account the structures of the tested catalysts, a correlation between the catalytic activity and the number of nitrogen atoms present in the catalyst molecule appears. Comparison of the performance of DBN and DBU to that of TBD and MTBD reveals that the presence of an additional nitrogen atom bonded to the amidine system in the latter increases the p*K*_BH+_ value, which has a direct impact on the catalytic activity of these bases.

Versatility of the methodology based on organocatalysis for obtaining silatranes is illustrated by a series of derivatives **3a**–**3s**, presented in Table 2. These silatranes were synthesized by a combination of TEOA and its derivatives (**1a**–**1f**) with organotrialkoxysilanes containing both inert and reactive groups (**2b**–**2n**) bonded to the silicon atom. In this synthetic work, DBU was used as a catalyst. In our opinion, the choice of this base perfectly illustrates the presented idea of using the catalytic properties of bicyclic amidine compounds in creation of silatranyl cages by a sequence of hydrolysis–condensation reactions.^52^ However, it is also a compromise between a sufficient catalytic activity of DBU and a high price of TBD and MTBD, which is crucial from a practical point of view. The data summarized in Table 2 show that the use of DBU as a catalyst allowed most products to be obtained in good to very good yields at room temperature. However, in some cases, heating up to 60 °C combined with a prolonged reaction time was necessary to ensure full conversion of the substrates.

**Table 2:**
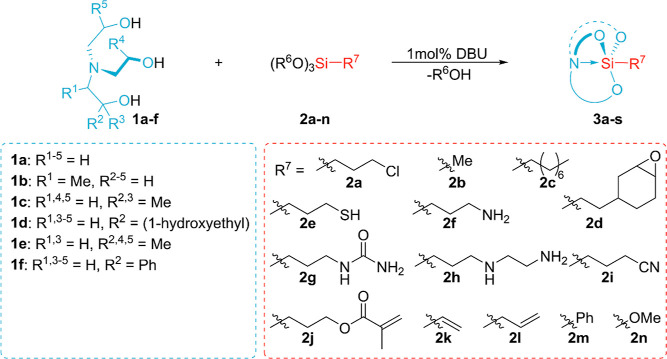
Synthesis of Organofunctionalized Silatranes^a^

entry	aminotriol	trialkoxysilane		*T* [°C]	*t* [h]	yield^b^ [%]	product
		R^6^	R^7^				
1	**1a**	–Me	**2a**	r.t.	1	99	**3a**
2	**1a**	–Me	**2b**	r.t.	6	98	**3b**
3	**1a**	–Me	**2c**	60	48	88	**3c**
4	**1a**	–Me	**2d**	r.t.	24	92	**3d**
5	**1a**	–Me	**2e**	50	12	95	**3e**
6	**1a**	–Et	**2f**	r.t.	24	84	**3f**
7	**1a**	–Me	**2g**	r.t.	1	96	**3g**
8	**1a**	–Me	**2h**	r.t.	24	90	**3h**
9	**1a**	–Et	**2i**	60	24	70	**3i**
10	**1a**	–Me	**2j**	r.t.	12	94^c^	**3j**
11	**1a**	–Et	**2k**	r.t.	6	90	**3k**
12	**1a**	–Me	**2l**	r.t.	1	94	**3l**
13	**1a**	–Me	**2m**	r.t.	0.5	95	**3m**
14	**1a**	–Me	**2n**	r.t.	18	99	**3n**
15	**1b**	–Me	**2a**	60	24	90	**3o**
16	**1c**	–Me	**2a**	60	24	75	**3p**
17	**1d**	–Me	**2a**	60	48	81	**3q**
18	**1e**	–Me	**2a**	60	12	70	**3r**
19	**1f**	–Me	**2a**	r.t.	24	98	**3s**

a Reaction conditions: [**1**]/[**2**]/[cat.] = 1:1.03:0.01, neat. A 12 mmol portion of **1** was used.

b Yields of isolated compounds.

c 0.1% of butylated hydroxytoluene was added.

To begin with, **3c** required a long time for homogenization of the reaction mixture due to a large difference in polarity between **2c** and TEOA. Additionally, increased steric hindrance either on a trialkoxysilane molecule, where EtO– groups are attached instead of MeO– (entries 6, 9, and 11 in Table 2), or on TEOA derivative (entries 15–18 in Table 2) significantly slowed down the reaction rate too. An exception is **3s** since the electron-withdrawing phenyl group enhanced the nucleophilic nature of the adjacent hydroxyl moiety. Thus, in this case, the reaction was conducted without heating. Despite a full conversion rate confirmed by gas chromatography (GC), isolation yields of some products were below 90% due to their partial solubility in hexane (**3c**, **3f**, **3i**, and **3p**–**3r**). Particularly, **3r** was easily soluble in *n*-hexane at room temperature; therefore, the washing process was performed with a reduced amount of a chilled solvent. Eventually, all compounds shown in Table 2 were isolated according to the method described above with yields over 70% and characterized spectroscopically. Moreover, for five of them (**3c**, **3g**, **3p**, **3q**, and **3s**), the structures were determined by X-ray analysis (see the Supporting Information).

**Figure 3:**
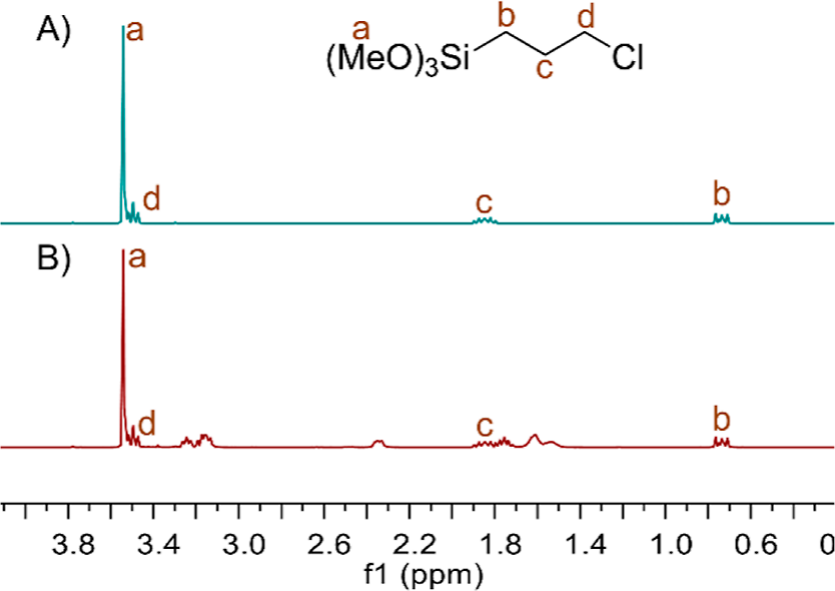
^1^H NMR spectra of (A) **2a** and (B) **2a** + DBU (CDCl_3_).

**Figure 4:**
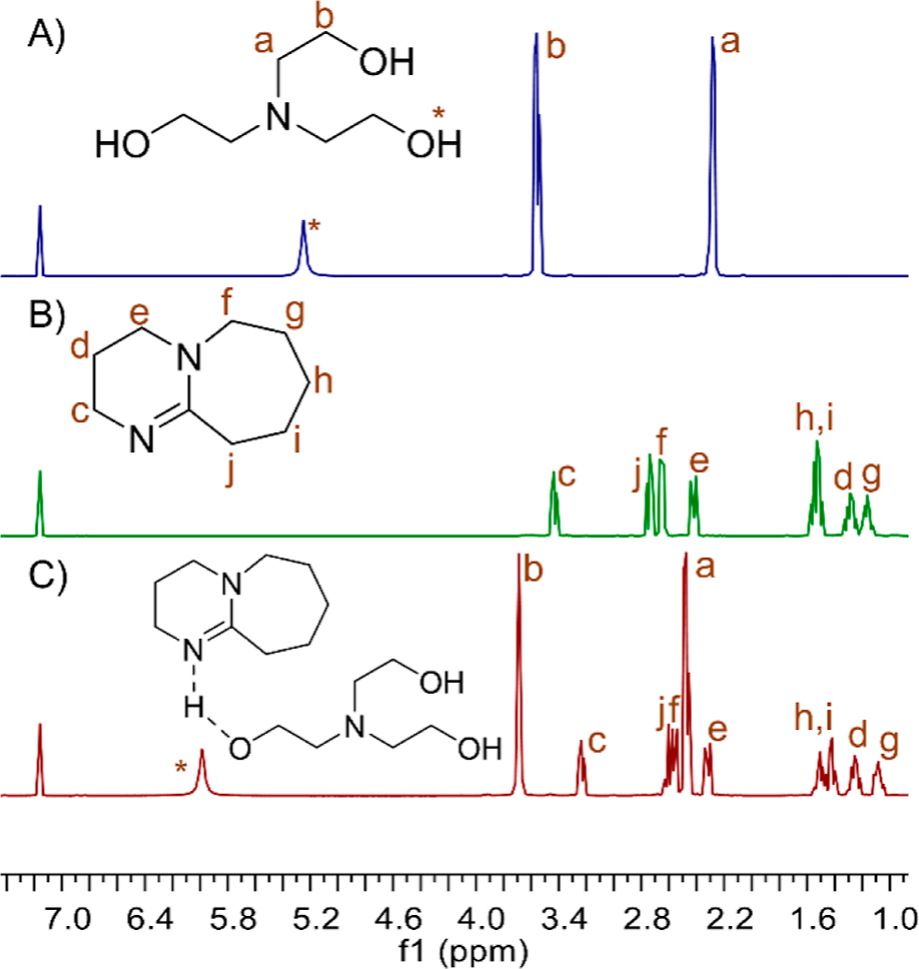
^1^H NMR spectra of (A) TEOA, (B) DBU, and (C) equimolar TEOA + DBU (C_6_D_6_). The peaks labeled with an asterisk refer to labile protons in hydrogen bonding.

**Figure 5:**
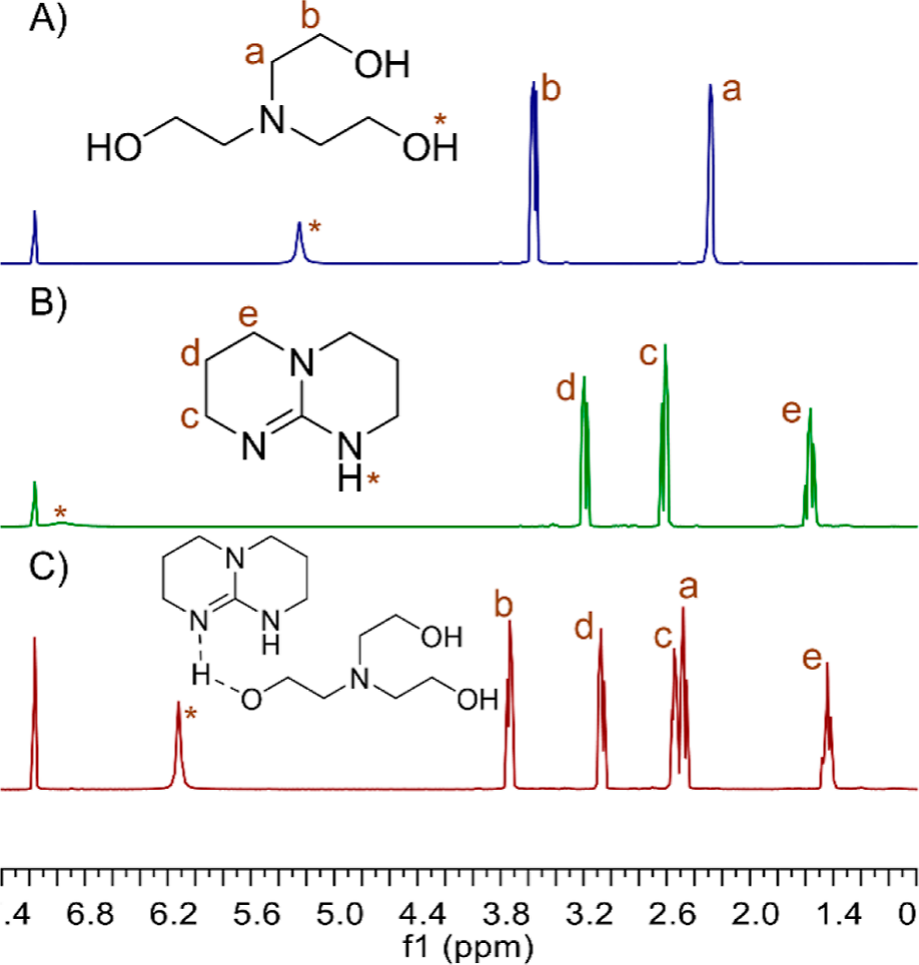
^1^H NMR spectra of (A) TEOA, (B) TBD, and (C) equimolar TEOA + TBD (C_6_D_6_). The peaks labeled with an asterisk refer to labile protons in hydrogen bonding.

To understand the catalytic role of organic amines based on the amidine system, a series of equimolar reactions between DBU and each reagent, namely, **2a** and TEOA, and additionally TBD and TEOA, were carried out followed by ^1^H NMR spectroscopy. The spectra are shown in Figures 3, 4, and 5.

Analysis of the recorded NMR spectra showed no interactions between DBU and **2a** used (Figure 3). However, in the case of the TEOA equimolar system with DBU (Figure 4), a clear shift of the resonance line originating from TEOA hydroxyl hydrogen atoms (5.98 ppm) was observed, compared to the position of these protons in the spectrum of the initial TEOA (5.25 ppm). Also, the lines originating from the ethylene moiety were shifted from 3.56 and 2.29 ppm for TEOA to 3.69 and 2.47 ppm for the TEOA + DBU system. This demonstrates the negative polarity of the oxygen atom and its surroundings. In addition, a change in the chemical shift and fine structure of all proton resonances was also observed in the DBU molecule. An analogous phenomenon was observed in the reaction of TEOA with TBD (Figure 5). The difference was that the hydroxyl protons experienced an even greater shift toward the lower field, up to 6.12 ppm. This may explain the even better activity of TBD in the studied process.

**Figure 6:**
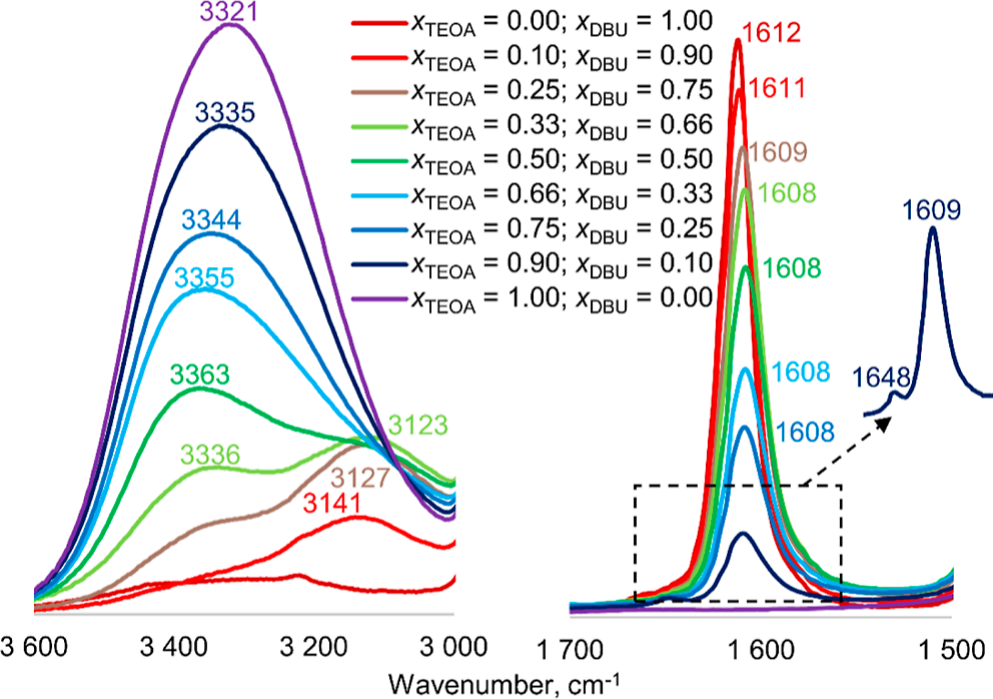
FTIR spectra of TEOA/DBU mixtures with different molar ratios in the selected regions.

Furthermore, for Fourier transform infrared (FTIR) spectroscopy, mixtures of TEOA and DBU were prepared in different ratios, and then, the positions of the characteristic absorption bands were compared (Figure 6). In the OH stretching region, pure TEOA shows a broad band at 3321 cm^–1^ corresponding to the O···H···O hydrogen bonding. The gradual addition of DBU causes a shift of the absorption band toward higher values of inverse centimeters, up to 3363 cm^–1^. When the molar ratio of DBU (*x*_DBU_) is increased to 0.66, a new vibration band from the O···H···N bonds system becomes predominant with a maximum at 3123 cm^–1^. DBU shows a sharp strong band at 1612 cm^–1^ attributed to the C=N ring stretching vibrations. Along with decrease of *x*_DBU_, a subtle red shift of the band maximum to 1608 cm^–1^ was observed. However, when *x*_DBU_ is as low as 0.1, an additional band is noticeable at 1648 cm^–1^, corresponding to the protonated imine group.^53,54^

**Figure 7:**
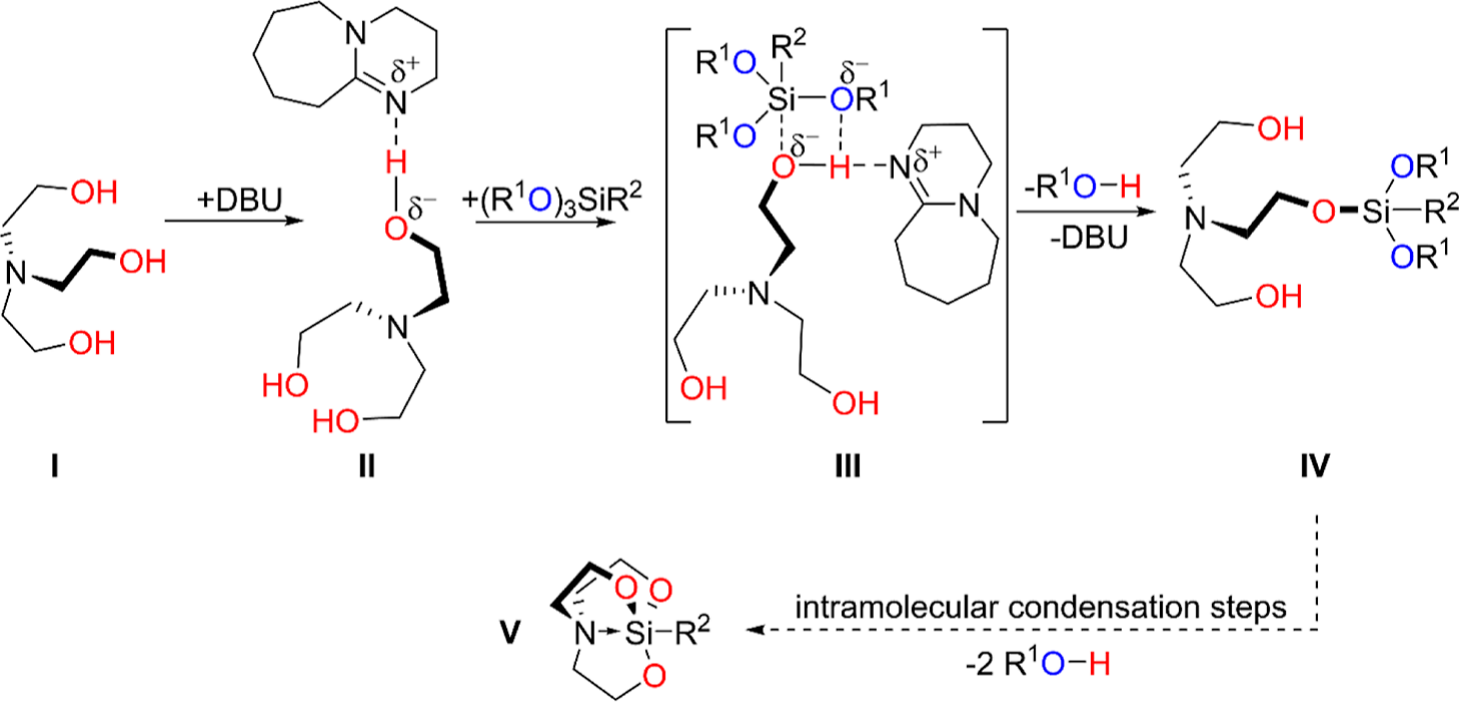
Proposed sequence of reactions illustrating the transesterification process.

The results of these studies allowed us to propose a diagram of the sequence of elementary reactions occurring during the transesterification process catalyzed by bicyclic amidine bases on the example of DBU (Figure 7). In the first step, TEOA (**I**) is activated by forming a relatively stable hydrogen bond with DBU (**II**), which is consistent with the earlier reports on similar subjects.^55^ We postulate that in the next step, the oxygen atom with increased negative polarization nucleophilically attacks the trialkoxysilane. Further, a four-centered transition state is formed, stabilized with the hydrogen bond O···H···N (**III**). In the next phase, metathesis of single bonds takes place, leading to the transesterification product (**IV**) with the evolution of an appropriate alcohol from the starting alkoxysilane and the release of the catalyst molecule (DBU). Finally, the remaining two hydroxyethyl arms undergo intramolecular condensation with the silicon center in the same manner as described above, affording the silatranyl cage (**V**).

A simple transesterification mechanism involving only acid–base interactions between DBU and the substrates enabled a high reaction rate and excellent selectivity. A full conversion rate was achieved for each of 19 silatrane derivatives (**3a**–**3s**) without the necessity to remove the generated alcohol under reduced pressure or using the Dean–Stark apparatus, which was common practice.^56−60^ Furthermore, all products were spectroscopically pure after washing with hexane without any additional purification step such as vacuum distillation, sublimation, recrystallization, or reprecipitation. In view of above, the developed reaction protocol is highly energy-efficient, sustainable, time-saving, and readily scalable, as indicated by a very high EcoScale score, losing only 10 penalty points from the hydrocarbon solvent safety in the case of most derivatives.^61^ In this protocol, hexane can be obviously replaced by more environmentally friendly hydrocarbons such as cyclohexane, *n*-heptane, *etc.* Silatrane derivatives equipped with highly polar substituents could also be washed with more sustainable solvents, such as ketones, esters, or ethers on the condition that the solubility is not significant. A detailed solubility study would certainly aid in determining the best workup solvent for each silatrane compound, contributing to the sustainability of the process even more.

**Figure 8:**
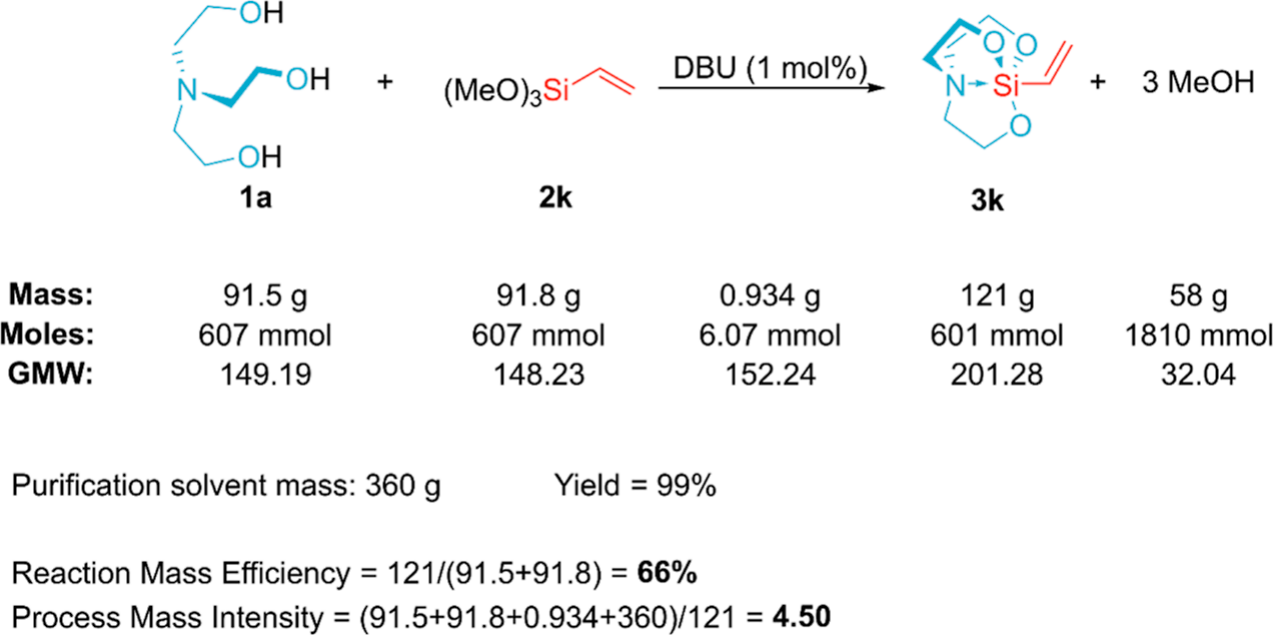
Green chemistry metrics of the scaled-up synthesis of **3k**.

In order to further validate this methodology, we attempted to scale-up the synthesis of 1-vinylsilatrane (**3k**, CAS: 2097-18-9) by 50-fold. We chose the vinyl derivative for this experiment because vinyltriethoxysilane is one of the key components in the manufacturing of glass fibers, which is industrially produced on a large scale.^62^ Besides, it is an all-round coupling agent used for versatile applications, including rubber, ceramics, drug carrier, catalyst, enzyme fixation, metal adhesion, *etc.*(63,64) Since silatrane derivatives exhibit superior surface modifying properties compared to the corresponding organotriethoxysilanes, as discussed in the Introduction section, we expect that 1-vinylsilatrane will meet a particularly high industrial demand. The reaction presented in Figure 8 was complete in 15 min. After the usual workup, 121 g of the target product was obtained, which is 99% of the theoretical yield. Furthermore, methanol was collected in a near-quantitative amount (58 g) by distillation into a cold trap. The portion of hexane used to wash the crude product was also fully recovered by distillation. The residue after concentration of the organic phase consisted of 79 mol % of DBU, 7 mol % of the target silatrane, residual solvents, and impurities already contained in the starting materials, according to the ^1^H NMR analysis. After replenishing the lost DBU, this mixture could be reused for the next batch of the reaction, facilitating cost efficiency even more in mass production. A substantial amount of methanol liberated and used hexane had a negative influence on some green chemistry metrics, such as the reaction mass efficiency (66%) and process mass intensity (4.50) of this reaction (Figure 8).^65^ However, these volatile substances are easy to recover by distillation, as mentioned earlier. Ultimately, 121 g of 1-vinylsilatrane was afforded using raw materials that cost 26.85 EUR in total, which gives 2.22 EUR per 10 g (calculation provided in the Supporting Information), whereas a 10 g package of 1-vinylsilatrane is sold on the retail market for ∼200 EUR, as of October 2023.^66^

**Figure 9:**
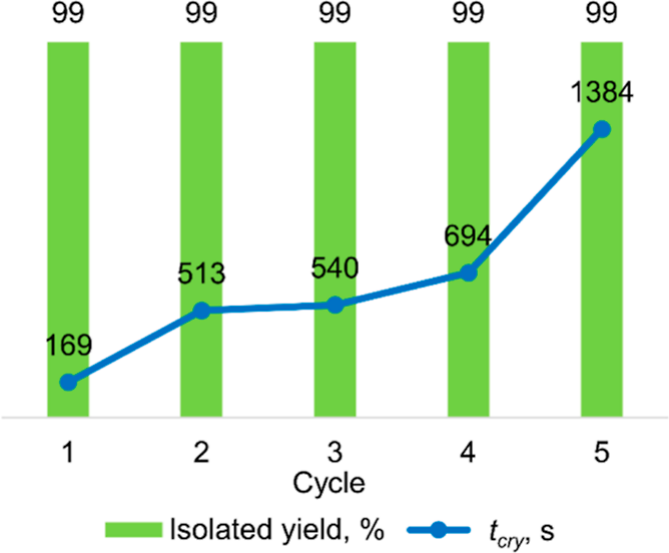
Recycling experiment of DBU in the synthesis of **3k**. Reaction conditions: [**1a**]/[**2k**]/[DBU] = 1:1.03:0.01, neat, r.t. A 12 mmol portion of **1a** was used per batch.

Finally, we performed DBU catalyst recycling tests in small-scale batch synthesis of the **3k** derivative (see the Supporting Information). We measured *t*_cry_ for five cycles, starting from a batch with pure DBU (cycle 1), and each consecutive cycle was catalyzed with the catalyst recovered from the previous batch. As can be deduced from the gathered *t*_cry_ values in Figure 9, the catalytic activity of the recycled catalyst decreases with each successive cycle. Particularly high escalation of *t*_cry_ was observed after cycles 1 and 4. Nevertheless, all batches afforded the desired product in near-quantitative yields within 40 min of the reaction, after a simple wash with hexane (see Figure 9). The decrease in the catalytic activity is primarily attributed to the mechanical loss of DBU and the accumulation of impurities. The matter of chemical deactivation of DBU can be neglected since amidines are not prone to hydrolysis at a low water content (<0.4%).^67^ The result of this experiment clearly proves the rationality and cost efficiency of the catalyst recycling process even without replenishing the lost DBU for up to four cycles.

**Scheme 1:**
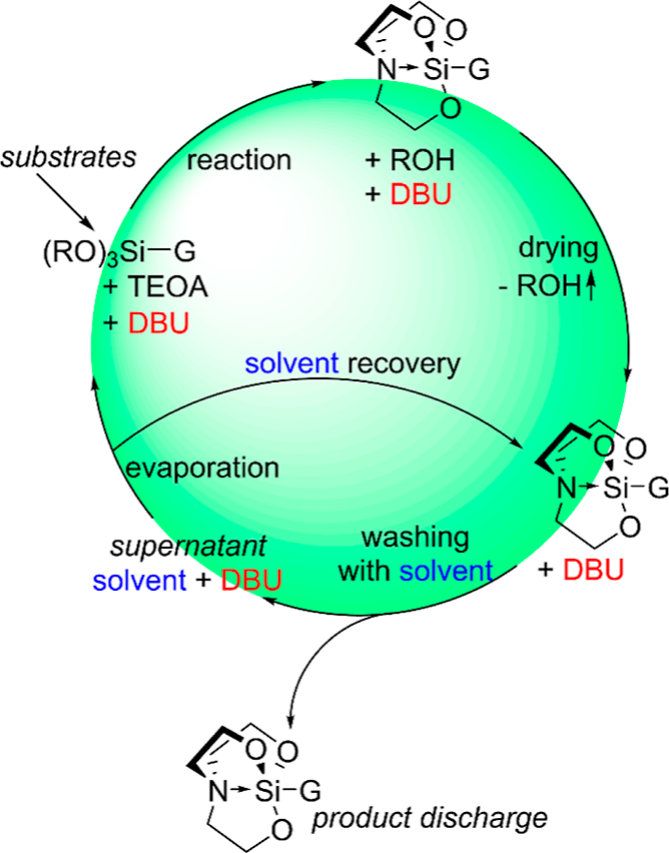
Proposed Industrial Production of Silatrane Derivatives with Regeneration of DBU and the Solvent R = Me or Et, G = general organic group.

Bearing in mind the successfully scaled-up synthesis of **3k** along with regeneration of DBU, we propose a scheme for the batch production process of silatrane derivatives for industrial application (Scheme 1). The presented idea assumes recycling of a single portion of the catalyst and hexane (or another appropriate solvent) through multiple batches, which contributes to the environmental friendliness of the entire reaction protocol even more. It should be emphasized that such catalyst regeneration is not possible with hitherto used inorganic catalysts, such as KOH,^23^ which are not soluble in hydrocarbon solvents. Additionally, the alcohol byproduct can be utilized in other processes held in the production facility or can be reused in the production of alkoxysilanes.

## Conclusions

In summary, here, we have reported an efficient methodology for transformation of trialkoxysilanes to the corresponding silatranes *via* the amidine-catalyzed process. Without a doubt, the great advantage of the developed protocol is that it turned out to be very versatile, in relation to both the triethanolamine derivatives used and silicon precursors equipped with various functional groups. The use of a relatively simple and cheap organocatalyst enabled the synthesis of 19 examples of silatranes with excellent yields. This proves the remarkable potential of the described method in the synthesis of new silatranyl equivalents of SCA on an industrial scale. Moreover, the reported protocol is in line with the principles of green chemistry. Furthermore, we have found a relation between the basicity of amidine derivatives and their catalytic activity. The NMR and FTIR spectroscopy studies of equimolar reactions of selected substrates with DBU and TBD revealed that in the reaction system studied, bases activate aminotriols, enabling a rapid transesterification process, leading to the rapid formation of a silatranyl cage. Finally, large-scale (0.6 mol) synthesis of the selected derivative (**3k**) was demonstrated in an efficiency equally high as that of the initial small-scale reaction, proving readiness of the methodology to be adopted in the industry. Furthermore, we confirmed the recycling ability of DBU in the batch synthesis of **3k**, which is a rare practice for this type of homogeneous organocatalytic system. We believe that this methodology will lower down the cost barrier for silatranes in both laboratory research and industrial application.

## Supporting Information Available

Materials, methods, procedures, spectroscopic data, and XRD (PDF)

## References

[ref1] Verkade J. G. Main Group Atranes: Chemical and Structural Features. Coord. Chem. Rev. 1994, 137, 233–295. 10.1016/0010-8545(94)03007-D.

[ref2] Puri J. K.; Singh R.; Chahal V. K. Silatranes: A Review on Their Synthesis, Structure, Reactivity and Applications. Chem. Soc. Rev. 2011, 40 (3), 1791–1840. 10.1039/B925899J. 21170448

[ref3] Frye C. L.; Vincent G. A.; Finzel W. A. Pentacoordinate Silicon Compounds. V. Novel Silatrane Chemistry. J. Am. Chem. Soc. 1971, 93 (25), 6805–6811. 10.1021/ja00754a017.

[ref4] Akiba K.-Y. Atrane and Transannular Interaction: Formation of Hypervalent Bond. In Organo Main Group Chemistry; John Wiley & Sons, Ltd, 2011; pp 201–211.

[ref5] Voronkov M. G.; Belyaeva V. V.; Abzaeva K. A. Basicity of Silatranes (Review). Chem. Heterocycl. Compd. 2012, 47 (11), 1330–1338. 10.1007/s10593-012-0918-1.

[ref6] Sok S.; Gordon M. S. A Dash of Protons: A Theoretical Study on the Hydrolysis Mechanism of 1-Substituted Silatranes and Their Protonated Analogs. Comput. Theor. Chem. 2012, 987, 2–15. 10.1016/j.comptc.2011.08.011.

[ref7] Ignatyev I. S.; Samokhin G. S.; Kochina T. A.; Belyaeva V. V.; Khaikin S. Ya.; Montejo M.; González J. L.; Voronkov M. G. Vibrational spectra and electronic structure of germatranols (HO)4–n Ge(OCH2CH2)nNR3–n (R = H; n = 1–3) with transannular Ge···N bonding. J. Organomet. Chem. 2013, 747, 62–68. 10.1016/j.jorganchem.2012.12.036.

[ref8] Marín-Luna M.; Alkorta I.; Elguero J. Theoretical Study of the Geometrical, Energetic and NMR Properties of Atranes. J. Organomet. Chem. 2015, 794, 206–215. 10.1016/j.jorganchem.2015.07.013.

[ref9] Singh R.; Mutneja R.; Kaur V.; Wagler J.; Kroke E. Derivatization of 3-Aminopropylsilatrane to Introduce Azomethine Linkage in the Axial Chain: Synthesis, Characterization and Structural Studies. J. Organomet. Chem. 2013, 724, 186–191. 10.1016/j.jorganchem.2012.11.009.

[ref10] Brennan B. J.; Gust D.; Brudvig G. W. Organosilatrane Building Blocks. Tetrahedron Lett. 2014, 55 (5), 1062–1064. 10.1016/j.tetlet.2013.12.082.

[ref11] Singh G.; Saroa A.; Girdhar S.; Rani S.; Choquesillo-Lazarte D.; Sahoo S. C. Incorporation of Azo Group at Axial Position of Silatranes: Synthesis, Characterization and Antimicrobial Activity. Appl. Organomet. Chem. 2015, 29 (8), 549–555. 10.1002/aoc.3330.

[ref12] Mutneja R.; Singh R.; Kaur V.; Wagler J.; Fels S.; Kroke E. Schiff Base Tailed Silatranes for the Fabrication of Functionalized Silica Based Magnetic Nano-Cores Possessing Active Sites for the Adsorption of Copper Ions. New J. Chem. 2016, 40 (2), 1640–1648. 10.1039/C5NJ02287H.

[ref13] Singh G.; Saroa A.; Rani S.; Promila; Girdhar S.; Sahoo S.; Choquesillo-Lazarte D. Substituted Phenyl Urea and Thiourea Silatranes: Synthesis, Characterization and Anion Recognition Properties by Photophysical and Theoretical Studies. Polyhedron 2016, 112, 51–60. 10.1016/j.poly.2016.03.036.

[ref14] Singh G.; Arora A.; Rani S.; Kalra P.; Aulakh D.; Wriedt M. A Family of Silatrane-Armed Triazole-Encapped Salicylaldehyde-Derived Schiff Bases: Synthesis, Spectral Analysis, and Antimicrobial and Quantum Chemical Evaluation. Appl. Organomet. Chem. 2017, 31 (10), e3728 10.1002/aoc.3728.

[ref15] Jain N.; Tripathi S. K.; Nasim M. Preparation and Characterization of Aminopropylsilatrane Endcapped Polyimide Films. Int. J. Polym. Mater. 2014, 63 (4), 178–184. 10.1080/00914037.2013.812089.

[ref16] Materna K. L.; Brennan B. J.; Brudvig G. W. Silatranes for Binding Inorganic Complexes to Metal Oxide Surfaces. Dalton Trans. 2015, 44 (47), 20312–20315. 10.1039/C5DT03463A. 26506505

[ref17] Singh G.; Rani S.; Saroa A.; Promila; Arora A.; Choquesillo-Lazarte D. Amide-Tethered Organosilatranes: Syntheses, Structural Characterization and Photophysical Properties. Inorg. Chim. Acta 2015, 433, 78–91. 10.1016/j.ica.2015.04.034.

[ref18] Voronkov M. G.; Baryshok V. P. Antitumor Activity of Silatranes (A Review). Pharm. Chem. J. 2004, 38 (1), 3–9. 10.1023/B:PHAC.0000027635.41154.0d.

[ref19] Rowe F. P.; Swinney T.; Bradfield A. Field trials of the rodenticide 5-*p*-chlorophenyl silatrane against wild house mice (*Mus musculus* L.). J. Hyg. 1974, 73 (1), 49–52. 10.1017/S0022172400023834. 4529040 PMC2130559

[ref20] Schröpl E.; Pohloude R. Acid isothiocyanates. 2. Reaction of acid isothiocyanates with aromatic amines yielding non-symmetrical disubstituted acylthioureas (I). Pharmazie 1968, 23 (9), 484–490. 5717186

[ref21] Sculimbrene B. R.; Decanio R. E.; Peterson B. W.; Muntel E. E.; Fenlon E. E. Silatranyl-Nucleosides: Transition State Analogues for Phosphoryl Transfer Reactions. Tetrahedron Lett. 2001, 42 (30), 4979–4982. 10.1016/S0040-4039(01)00942-X.

[ref22] Ye F.; Song X.; Liu J.; Xu X.; Wang Y.; Hu L.; Wang Y.; Liang G.; Guo P.; Xie Z. Design, Synthesis, and Biological Evaluation of γ-Aminopropyl Silatrane-Acyclovir Hybrids with Immunomodulatory Effects. Chem. Biol. Drug Des. 2015, 86 (4), 905–910. 10.1111/cbdd.12519. 25599975

[ref23] Han A.; Li L.; Qing K.; Qi X.; Hou L.; Luo X.; Shi S.; Ye F. Synthesis and Biological Evaluation of Nucleoside Analogues than Contain Silatrane on the Basis of the Structure of Acyclovir (ACV) as Novel Inhibitors of Hepatitis B Virus (HBV). Bioorg. Med. Chem. Lett. 2013, 23 (5), 1310–1314. 10.1016/j.bmcl.2012.12.097. 23369536

[ref24] Chen L.; Xie Q.; Sun L.; Wang H. Synthesis and Characterization of 1-Ferrocenecarboxysilatranes and Crystal Structures of FcC(CH_3_)=CHCOOSi(OCH_2_CH_2_)_3_N and *p*-FcC_6_H_4_COOSi(OCH_2_CH_2_)_3_N. J. Organomet. Chem. 2003, 678 (1–2), 90–94. 10.1016/S0022-328X(03)00449-2.

[ref25] Li Z.; Song X.; Su H.; Chen J. Synthesis of 1-substituted benzoyl aminopropyl silatranes and their biological activities. Heterocycl. Commun. 2005, 11 (6), 475–478. 10.1515/HC.2005.11.6.475.

[ref26] Singh G.; Arora A.; Mangat S. S.; Rani S.; Kaur H.; Goyal K.; Sehgal R.; Maurya I. K.; Tewari R.; Choquesillo-Lazarte D.; Sahoo S.; Kaur N. Design, Synthesis and Biological Evaluation of Chalconyl Blended Triazole Allied Organosilatranes as Giardicidal and Trichomonacidal Agents. Eur. J. Med. Chem. 2016, 108, 287–300. 10.1016/j.ejmech.2015.11.029. 26695730

[ref27] Singh G.; Saroa A.; Girdhar S.; Rani S.; Sahoo S.; Choquesillo-Lazarte D. Synthesis, Characterization, Electronic Absorption and Antimicrobial Studies of N-(Silatranylpropyl)Phthalimide Derived from Phthalic Anhydride. Inorg. Chim. Acta 2015, 427, 232–239. 10.1016/j.ica.2015.01.011.

[ref28] Yao N. I. E.; Dong-Hai H.; Fa-Song W.; You-Meng D. a. N.; Jin-Shun Z.; Xin-Jian S. Synthesis, Crystal Structures, and Antitumor Activity of Two 2-Trifluoromethyl-5,6,7,8-tetrahydrobenzo[4,5]thieno[2,3-d]pyrimidin-4-amine Derivatives. Chin. J. Struct. Chem. 2014, 33 (12), 1789–1795. 10.14102/j.cnki.0254-5861.2011-0478.

[ref29] Huang C.-J.; Zheng Y.-Y. Controlled Silanization Using Functional Silatrane for Thin and Homogeneous Antifouling Coatings. Langmuir 2019, 35 (5), 1662–1671. 10.1021/acs.langmuir.8b01981. 30086630

[ref30] Heinig M. F.; Bastos da Silva Fanta A.; Wagner J. B.; Kadkhodazadeh S. Aminopropylsilatrane Linkers for Easy and Fast Fabrication of High-Quality 10 Nm Thick Gold Films on SiO2 Substrates. ACS Appl. Nano Mater. 2020, 3 (5), 4418–4427. 10.1021/acsanm.0c00531.

[ref31] Lee T.-J.; Chau L.-K.; Huang C.-J. Controlled Silanization: High Molecular Regularity of Functional Thiol Groups on Siloxane Coatings. Langmuir 2020, 36 (21), 5935–5943. 10.1021/acs.langmuir.0c00745. 32388989

[ref32] Materna K. L.; Rudshteyn B.; Brennan B. J.; Kane M. H.; Bloomfield A. J.; Huang D. L.; Shopov D. Y.; Batista V. S.; Crabtree R. H.; Brudvig G. W. Heterogenized Iridium Water-Oxidation Catalyst from a Silatrane Precursor. ACS Catal. 2016, 6 (8), 5371–5377. 10.1021/acscatal.6b01101.

[ref33] Bajada M. A.; Roy S.; Warnan J.; Abdiaziz K.; Wagner A.; Roessler M. M.; Reisner E. A Precious-Metal-Free Hybrid Electrolyzer for Alcohol Oxidation Coupled to CO2-to-Syngas Conversion. Angew. Chem., Int. Ed. 2020, 59 (36), 15633–15641. 10.1002/anie.202002680. PMC7496929 32250531

[ref34] Hasse A.; Korth K.; Kiefer I.; Witzsche S.; Albert P.; Klockmann O. Rubber Mixtures. U.S. Patent 8,252,863 B2, August 28, 2012.

[ref35] Saiki T.; Iwai M.; Tomar A. K. Method for Manufacturing a Bis(Silatranylalkyl) Polysulfide, Method for Manufacturing a Mixture of Bis(Silatranylalkyl) Polysulfide Etc., a Mixture of Bis(Silatranylalkyl) Polysulfide Etc., and Rubber Composition. U.S. Patent 8,093,323 B2, January 10, 2012.

[ref36] Korth K.; Hasse A.; Witzsche S.; Klockmann O.; Albert P. Organosilicon Compounds Their Preparation and Their Use. U.S. Patent 20,070,066,760 A1, March 22, 2007.

[ref37] Ma L.; Vielhaber M. M. Functionalized Polymer, Rubber Composition, and Pneumatic Tire. U.S. Patent 9,790,289 B1, October 17, 2017.

[ref38] Cheng H.; Laine R. M. Simple, Low-Cost Synthetic Route to Potentially Polymerizable Silatranes. New J. Chem. 1999, 23 (12), 1181–1186. 10.1039/a908291c.

[ref39] Nasim M.; Tharmaraj P.; Venkataramani P. S. Heterocyclic Substituted Silatranes. Part I. Synthesis and Characterization of Pyrazolyl Substituted Aminoalkylsilatranes. Synth. React. Inorg. Met.-Org. Chem. 1999, 29 (7), 1249–1263. 10.1080/00945719909349526.

[ref40] Kovács I.; Matern E.; Sattler E.; Anson C. E.; Párkányi L. The Synthesis, Crystal Structures and NMR Spectroscopic Investigation of 3,7,10-Trimethylsilatranes and Carbasilatranes. J. Organomet. Chem. 2009, 694 (1), 14–20. 10.1016/j.jorganchem.2008.09.055.

[ref41] Verma S. C.; Nasim M.; Venkataramani P. S. Synthesis and Characterization of 3-[2-(2-Aminoethylamino)Ethylamino]Propyl-1-Silatranes. Indian J. Chem., Sect B: Org. Med. Chem. 2004, 43 (8), 1737–1742.

[ref42] Voronkov M. G.; Zel’bst E. A.; Kashaev A. A.; Katkevich Yu. V.; Fundamenskii V. S.; Bolgova Yu. I.; Trofimova O. M.; Belyaeva V. V.; Chernov N. F. Crystal and Molecular Structure of N-(1-Silatranylmethyl)Carbazole. Dokl. Chem. 2003, 389 (4/6), 69–72. 10.1023/A:1023432322387.

[ref43] Voronkov M. G.; Kuznetsova G. A.; Baryshok V. P. A New Route to 1-Chlorosilatrane. Russ. J. Gen. Chem. 2010, 80 (10), 1926–1928. 10.1134/S1070363210100075.

[ref44] Voronkov M. G.; Kuznetsova G. A. Direct Synthesis of 1-Organylsilatranes from Organyltrichlorosilanes and Tris(2-Hydroxyethyl)Amine. Russ. J. Gen. Chem. 2009, 79 (5), 925–927. 10.1134/S1070363209050107.

[ref45] Gevorgyan V.; Borisova L.; Lukevics E. Organylsilatranes from the Reaction of Tetraorganylsilanes with Triethanolamine. J. Organomet. Chem. 1997, 527 (1–2), 295–296. 10.1016/S0022-328X(96)06652-1.

[ref46] Gevorgyan V.; Borisova L.; Vyater A.; Ryabova V.; Lukevics E. A Novel Route to Pentacoordinated Organylsilanes and -Germanes. J. Organomet. Chem. 1997, 548 (2), 149–155. 10.1016/S0022-328X(97)00454-3.

[ref47] Pedersen B.; Wagner G.; Herrmann R.; Scherer W.; Meerholz K.; Schmälzlin E.; Bräuchle C. Ferrocenylethenylsilatranes and a Cymantrenylsilatrane. J. Organomet. Chem. 1999, 590 (2), 129–137. 10.1016/S0022-328X(99)00440-4.

[ref48] Junker C. S.; Welker M. E.; Day C. S. Synthesis of 4-Aryl- and 4-Alkyl-2-Silyl-1,3-Butadienes and Their Diels-Alder/Cross-Coupling Reactions. J. Org. Chem. 2010, 75 (23), 8155–8165. 10.1021/jo1017734. 21069964

[ref49] Varjosaari S. E.; Skrypai V.; Suating P.; Hurley J. J. M.; Gilbert T. M.; Adler M. J. 1-Hydrosilatrane: A Locomotive for Efficient Ketone Reductions. Eur. J. Org Chem. 2017, 2017 (2), 229–232. 10.1002/ejoc.201601256.

[ref50] Anastas P.; Warner J.; Anastas P.; Warner J. Green Chemistry: Theory and Practice; Oxford University Press: Oxford, NY, 2000.

[ref51] Tshepelevitsh S.; Kütt A.; Lõkov M.; Kaljurand I.; Saame J.; Heering A.; Plieger P. G.; Vianello R.; Leito I. On the Basicity of Organic Bases in Different Media. Eur. J. Org Chem. 2019, 2019 (40), 6735–6748. 10.1002/ejoc.201900956.

[ref52] Kownacki I.; Oh M. J. New Organofunctional Silatranes and a New Catalytic Method for Synthesis of Novel and Known Organofunctional Silatranes, as well as Their Application as Silane Coupling Agents for Preparation of Rubber Compounds, April 18, 2023; p 444474.

[ref53] Heldebrant D. J.; Jessop P. G.; Thomas C. A.; Eckert C. A.; Liotta C. L. The Reaction of 1,8-Diazabicyclo[5.4.0]Undec-7-Ene (DBU) with Carbon Dioxide. J. Org. Chem. 2005, 70 (13), 5335–5338. 10.1021/jo0503759. 15960544

[ref54] Anugwom I.; Mäki-Arvela P.; Virtanen P.; Damlin P.; Sjöholm R.; Mikkola J.-P. Switchable Ionic Liquids (SILs) Based on Glycerol and Acid Gases. RSC Adv. 2011, 1 (3), 452–457. 10.1039/c1ra00154j.

[ref55] Wang B.; Luo Z.; Elageed E. H. M.; Wu S.; Zhang Y.; Wu X.; Xia F.; Zhang G.; Gao G. DBU and DBU-Derived Ionic Liquid Synergistic Catalysts for the Conversion of Carbon Dioxide/Carbon Disulfide to 3-Aryl-2-Oxazolidinones/[1,3]Dithiolan-2-Ylidenephenyl- Amine. ChemCatChem 2016, 8 (4), 830–838. 10.1002/cctc.201500928.

[ref56] Mizumo T.; Nakashima M.; Ohshita J. Oligosiloxanes with Silatrane Moieties for Use in Lithium-Ion Conductive Matrices. Silicon 2017, 9 (1), 85–96. 10.1007/s12633-014-9187-1.

[ref57] Mizumo T.; Kajihara T.; Yamada T.; Ohshita J. Preparation and Utilization of Poly(Methacryloylsilatrane) as a Salt-Dissociation Enhancer in PEO-Based Polymer Electrolytes. Polym. Adv. Technol. 2013, 24 (8), 705–714. 10.1002/pat.3134.

[ref58] Kovács I.; Matern E.; Sattler E.; Anson C. E.; Párkányi L. The Synthesis, Crystal Structures and NMR Spectroscopic Investigation of 3,7,10-Trimethylsilatranes and Carbasilatranes. J. Organomet. Chem. 2009, 694 (1), 14–20. 10.1016/j.jorganchem.2008.09.055.

[ref59] Singh G.; Sushma; Priyanka; Suman; Diksha; Kaur J. D.; Saini A.; Devi A.; Satija P. Synthesis, Characterization and UV-Visible Study of Schiff Base-Acetylene Functionalized Organosilatrane Receptor for the Dual Detection of Zn2+ and Co2+ Ions. Inorg. Chim. Acta 2021, 525, 120465 10.1016/j.ica.2021.120465.

[ref60] Singh G.; Rani S.; Arora A.; Aulakh D.; Wriedt M. Thioester-Appended Organosilatranes: Synthetic Investigations and Application in the Modification of Magnetic Silica Surfaces. New J. Chem. 2016, 40 (7), 6200–6213. 10.1039/C6NJ00011H.

[ref61] Van Aken K.; Strekowski L.; Patiny L. EcoScale, a Semi-Quantitative Tool to Select an Organic Preparation Based on Economical and Ecological Parameters. Beilstein J. Org. Chem. 2006, 2 (1), 3 10.1186/1860-5397-2-3. 16542013 PMC1409775

[ref62] Thomason J. L. Glass Fibre Sizing: A Review. Composites, Part A 2019, 127, 105619 10.1016/j.compositesa.2019.105619.

[ref63] Picard L.; Phalip P.; Fleury E.; Ganachaud F. Chemical Adhesion of Silicone Elastomers on Primed Metal Surfaces: A Comprehensive Survey of Open and Patent Literature. Prog. Org. Coat. 2015, 80, 120–141. 10.1016/j.porgcoat.2014.11.022.

[ref64] Zhang L.; Chang Z. X.; Li D. L. The Surface Modification of Silica with Vinyltriethoxysilane. Adv. Mater. Res. 2011, 399–401, 1123–1130. 10.4028/www.scientific.net/AMR.399-401.1123.

[ref65] Constable D. J. C.; Curzons A. D.; Cunningham V. L. Metrics to ‘Green’ Chemistry—Which Are the Best?. Green Chem. 2002, 4 (6), 521–527. 10.1039/B206169B.

[ref66] Price retrieved from the CHEMAT online catalogue (chemat.com.pl).

[ref67] Hyde A. M.; Calabria R.; Arvary R.; Wang X.; Klapars A. Investigating the Underappreciated Hydrolytic Instability of 1,8-Diazabicyclo[5.4.0]Undec-7-Ene and Related Unsaturated Nitrogenous Bases. Org. Process Res. Dev. 2019, 23 (9), 1860–1871. 10.1021/acs.oprd.9b00187.

