# Supplementary material for: Solvent-Free and Efficient Synthesis of Silatranes via an Organocatalytic Protocol under Mild Conditions
Source: ACS Sustain Chem Eng. 2024 Jan 10;12(5):2049–57. doi: 10.1021/acssuschemeng.3c07293 (PMC10848291; doi:10.1021/acssuschemeng.3c07293)
Supplement: Supplementary file 1 — sc3c07293_si_001.pdf [file sc3c07293_si_001.pdf]

A solvent-free and efficient synthesis of silatranes *via* organocatalytic protocol under mild conditions

Myong Joon Oh[a,b], Ireneusz Kownacki*[a,b], Maciej Kubicki[a]

[a]Faculty of Chemistry, Adam Mickiewicz University in Poznań, St. Uniwersytetu Poznańskiego 8, 61-614 Poznań, Poland.

[b]Center for Advanced Technology, Adam Mickiewicz University in Poznań, St. Uniwersytetu Poznańskiego 10, 61-614 Poznań, Poland.

*Corresponding Author. e-mail: ireneusz.kownacki@amu.edu.pl

Table of contents

1. Materials S2
2. Methods S2
3. Procedure for catalyst screening S2
4. General synthesis of silatranes **3a** – **3s** S2
5. Large-scale synthesis of **3k** S7
6. Catalyst regeneration S8
7. NMR spectra S9
8. HRMS-ESI Spectra S38
9. Single crystal X-ray crystallography S47
10. References S52

Number of pages: 52

Number of figures: 82

Number of tables: 3

1. Materials

Triethanolamine, triisopropanolamine, hexane (fraction from petroleum), triethylamine, *N*,*N*-diisopropylethylamine, 4-(dimethylamino)pyridine, 1,4-diazabicyclo[2.2.2]octane, 1,5-diazabicyclo[4.3.0]non-5-ene, 1,8-diazabicyclo[5.4.0]undec-7-ene, 1,5,7-triazabicyclo[4.4.0]dec-5-ene and 7-methyl-1,5,7-triazabicyclo[4.4.0]dec-5-ene were purchased from Sigma Aldrich. (3-chloropropyl)trimethoxysilane, methyltrimethoxysilane, [2-(3,4-epoxycyclohexyl)ethyl]trimethoxysilane, (3-mercaptopropyl)trimethoxysilane, (3-aminopropyl)triethoxysilane, (3-ureidopropyl)trimethoxysilane, [3-(2-aminoethylamino)propyl]trimethoxysilane, [3-(methacryloyloxy)propyl]trimethoxysilane, (3-cyanopropyl)triethoxysilane, vinyltriethoxysilane, vinyltrimethoxysilane, allyltrimethoxysilane, phenyltrimethoxysilane and tetramethoxysilane were purchased from ABCR. 2,2'-((1-hydroxypropan-2-yl)azanediyl)bis(ethan-1-ol), 2,2'-((2-hydroxy-2-methylpropyl)azanediyl)bis(ethan-1-ol), 1-(bis(2-hydroxyethyl)amino)butane-2,3-diol and 2,2'-((2-hydroxy-2-phenylethyl)azanediyl)bis(ethan-1-ol) were delivered by Trimen Chemicals. 2,6-Di-*tert*-butyl-4-methylphenol was purchased from TCI Chemicals. All chemicals were used as received.

2. Methods

All reactions were conducted in argon atmosphere. Standard Schlenk techniques were used unless specified otherwise. The reaction progress was monitored by means of gas chromatograph Bruker Scion 436-GC. ^{1}H NMR, ^{13}C NMR and ^{29}Si NMR spectra were recorded with either a Bruker Ultrashield 300 MHz spectrometer or a Bruker Ascend NanoBay 400 MHz spectrometer. $CDCl_3$, $(CD_3)_2CO$, CD_3OD, CD_3CN and C_6D_6 were used as solvents for NMR analysis with residual solvent peak as the internal standard ($CDCl_3$: ^{1}H δ = 7.26 ppm, ^{13}C δ = 77.16 ppm; $(CD_3)_2CO$: ^{1}H δ = 2.05 ppm, ^{13}C δ = 29.84 ppm; CD_3OD: ^{1}H δ = 3.31 ppm, ^{13}C δ = 49.00 ppm; CD_3CN: ^{1}H δ = 1.94 ppm, ^{13}C δ = 1.32 ppm; C_6D_6: ^{1}H δ = 7.16 ppm). High-resolution mass spectra were acquired in electrospray ionization mode (ESI) with time of flight (TOF) mass detector on an Impact HD Bruker Daltonics QTOF spectrometer or an AB Sciex 5600 QTOF spectrometer.

3. Procedure for catalyst screening

To a 50 ml single-neck round-bottom flask (Duran®) equipped with a 13 x 8 mm octahedral PTFE stirring bar (Cowie Technology™), ca. 1.4 g of triethanolamine (**1a**) and stoichiometric amount of 3-chloropropyltrimethoxysilane (**2a**) were introduced. The mixture was stirred on a magnetic stirrer with rotation speed of 500 rpm at room temperature. Then, a catalyst was added to the mixture and t_{cry} was measured, mixing the mixture continuously. The experiment was repeated three times and average t_{cry} values are shown in the main article text. All reactions for the catalyst screening were conducted using exactly the same equipment in order to maintain the same crystallization condition.

4. General synthesis of silatranes 3a – 3s

In an inert atmosphere, triethanolamine or its derivative (**1a** – **1f**) (1.00 eq., 12 mmol), trialkoxysilane (**2a** – **2n**) (1.03 eq.) and DBU (0.01 eq.) were placed in a dry round-bottomed flask (see Table 2 in the main text). The mixture was stirred vigorously at r.t. – 60 °C for 0.5 – 48 h until full conversion of the substrates was reached, monitored by GC. Then, the reaction was kept under vacuum to remove the volatile alcohol side product. The remaining precipitate was collected and washed with three portions of hexane (ca. 15 ml in total) to afford a spectroscopically pure, white crystalline solid.

1-(3-chloropropyl)silatrane (3a)

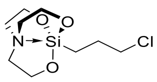

Reaction conditions: r.t., 2h. 99 % yield. ^{1}H NMR (400 MHz, Methanol-d_4) δ 3.76 (t, J = 5.9 Hz, 6H), 3.47 (t, J = 7.4 Hz, 2H), 2.90 (t, J = 5.9 Hz, 6H), 1.92 – 1.67 (m, 2H), 0.50 – 0.27 (m, 2H). ^{13}C NMR (101 MHz, Methanol-d_4) δ 58.46, 51.69, 49.19, 30.46, 15.56. ^{29}Si NMR (79 MHz, Methanol-d_4) δ -71.12. HRMS (ESI): m/z calcd for $C_9H_{18}ClNO_3Si^+$, [M+H]$^+$: 252.0817. Found: 252.0821.

1-methylsilatrane (3b)

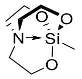

Reaction conditions: r.t., 6h. 98 % yield. ^{1}H NMR (400 MHz, Chloroform-d) δ 3.76 (t, J = 5.8 Hz, 6H), 2.79 (t, J = 5.8 Hz, 6H), -0.08 – -0.12 (m, 3H). ^{13}C NMR (101 MHz, Chloroform-d) δ 57.86, 51.10, -1.48. ^{29}Si NMR (79 MHz, Chloroform-d) δ -64.64. HRMS (ESI): m/z calcd for $C_7H_{15}NO_3Si^+$, [M+Na]$^+$: 212.0718. Found: 212.0717.

1-octylsilatrane (3c)

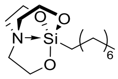

Reaction conditions: 60 °C, 48h. 88 % yield. ^{1}H NMR (400 MHz, Chloroform-d) δ 3.74 (t, J = 5.8 Hz, 6H), 2.77 (t, J = 5.8 Hz, 6H), 1.48 – 1.09 (m, 12H), 0.84 (t, J = 6.8 Hz, 3H), 0.48 – 0.31 (m, 2H). ^{13}C NMR (101 MHz, Chloroform-d) δ 58.08, 51.30, 34.16, 32.12, 29.65, 29.53, 25.21, 22.81, 16.58, 14.26. ^{29}Si NMR (79 MHz, Chloroform-d) δ -64.76. HRMS (ESI): m/z calcd for $C_{14}H_{29}NO_3Si^+$, [M+H]$^+$: 288.1989. Found: 288.1975.

1-[2-(3,4-epoxycyclohexyl)ethyl]silatrane (3d), mixture of diastereoisomers

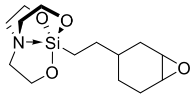

Reaction conditions: r.t., 24h. 92 % yield. ^{1}H NMR (400 MHz, Chloroform-d) δ 3.73 (t, J = 5.8 Hz, 6H), 3.16 – 3.02 (m, 2H), 2.77 (t, J = 5.8 Hz, 6H), 2.22 – 1.87 (m, 2H), 1.84 – 1.57 (m, 1H), 1.54 – 0.76 (m, 6H), 0.42 – 0.28 (m, 2H). ^{13}C NMR (101 MHz, Chloroform-d) δ 57.89, 57.86, 53.45, 52.90, 52.32, 52.20, 51.13, 51.11, 36.09, 32.49, 32.02, 31.39, 31.27, 30.55, 26.56, 25.60, 24.25, 23.57, 13.37, 13.15. ^{29}Si NMR (79 MHz, Chloroform-d) δ -65.22, -65.46. HRMS (ESI): m/z calcd for $C_{14}H_{25}NO_4Si^+$, [M+H]$^+$: 300.1626. Found: 300.1613.

1-(3-mercaptopropyl)silatrane (3e)

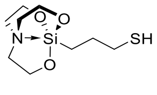

Reaction conditions: 50 °C, 12h. 95 % yield. ^{1}H NMR (400 MHz, Methanol-d_4) δ 3.76 (t, J = 5.9 Hz, 6H), 2.90 (t, J = 5.9 Hz, 6H), 2.45 (t, J = 7.3 Hz, 2H), 1.79 – 1.58 (m, 2H), 0.55 – 0.30 (m, 2H). ^{13}C NMR (101 MHz, Methanol-d_4) δ 58.50, 51.70, 31.56, 28.83, 17.22. ^{29}Si NMR (79 MHz, Methanol-d_4) δ -70.40. HRMS (ESI): m/z calcd for $C_9H_{19}NO_3SSi$+, [M+H]$^+$: 250.0928. Found: 250.0928.

1-(3-aminopropyl)silatrane (3f)

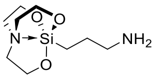

Reaction conditions: r.t., 24h. 84 % yield. ^{1}H NMR (400 MHz, Chloroform-*d*) δ 3.73 (t, *J* = 5.8 Hz, 6H), 2.77 (t, *J* = 5.8 Hz, 6H), 2.59 (t, *J* = 7.0 Hz, 2H), 1.60 – 1.41 (m, 2H), 1.01 (s, 2H), 0.48 – 0.30 (m, 2H). ^{13}C NMR (101 MHz, Chloroform-*d*) δ 57.91, 51.22, 45.90, 30.00, 13.09. ^{29}Si NMR (79 MHz, Chloroform-*d*) δ -66.00. HRMS (ESI): m/z calcd for $C_9H_{18}NO_3Si^+$, $[M+H]^+$: 233.1316. Found: 233.1308.

1-(3-ureidopropyl)silatrane (3g)

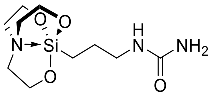

Reaction conditions: r.t., 1h. 96 % yield. The crude product was washed with ethyl acetate instead of hexane. ^{1}H NMR (400 MHz, Methanol-d_4) δ 3.75 (t, *J* = 5.9 Hz, 6H), 3.03 (t, *J* = 7.1 Hz, 2H), 2.89 (t, *J* = 5.9 Hz, 6H), 1,62 – 1,48 (m, 2H), 0.41 – 0.26 (m, 2H). ^{13}C NMR (101 MHz, Methanol-d_4) δ 162.39, 58.50, 51.70, 44.45, 26.62, 14.77. ^{29}Si NMR (79 MHz, Methanol-d_4) δ -70.15. HRMS (ESI): m/z calcd for $C_{10}H_{21}N_3O_4Si^+$, $[M+H]^+$: 276.1374. Found: 276.1365.

1-[3-(2-aminoethylamino)propyl]silatrane (3h)

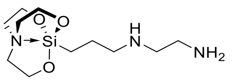

Reaction conditions: r.t., 24h. 90 % yield. ^{1}H NMR (400 MHz, Chloroform-*d*) δ 3.72 (t, *J* = 5.8 Hz, 6H), 2.80 – 2.70 (m, 8H), 2.65 – 2.59 (m, 2H), 2.55 (t, *J* = 7.2 Hz, 2H), 1.65 – 1.50 (m, 2H), 1.08 (s, 3H), 0.45 – 0.32 (m, 2H). ^{13}C NMR (101 MHz, Chloroform-*d*) δ 57.91, 53.21, 52.68, 51.24, 42.26, 25.44, 13.53. ^{29}Si NMR (79 MHz, Chloroform-*d*) δ -65.75. HRMS (ESI): m/z calcd for $C_{11}H_{25}N_3O_3Si^+$, $[M+H]^+$: 276.1738. Found: 276.1730.

1-[3-(methacryloyloxy)propyl]silatrane (3i)

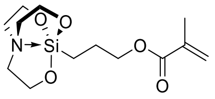

Reaction conditions: r.t., 12h, with addition of 0.001 eq. of BHT. 94 % yield. ^{1}H NMR (400 MHz, Acetone-d_6) δ 6.03 (dq, *J* = 2.0, 1.0 Hz, 1H), 5.57 (p, *J* = 1.7 Hz, 1H), 4.01 (t, *J* = 7.3 Hz, 2H), 3.66 (t, *J* = 5.9 Hz, 6H), 2.85 (t, *J* = 5.8 Hz, 6H), 1.90 (t, *J* = 1.3 Hz, 3H), 1.78 – 1.64 (m, 2H), 0.39 – 0.20 (m, 2H). ^{13}C NMR (101 MHz, Acetone-d_6) δ 167.54, 137.94, 124.83, 68.58, 58.04, 51.48, 25.58, 18.47, 13.46. ^{29}Si NMR (79 MHz, Acetone-d_6) δ -69.57. HRMS (ESI): m/z calcd for $C_{13}H_{23}NO_5Si^+$, $[M+H]^+$: 302.1418. Found: 302.1405.

1-(3-cyanopropyl)silatrane (3j)

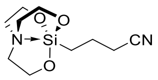

Reaction conditions: 60 °C, 24h. 70 % yield. ^{1}H NMR (400 MHz, Chloroform-*d*) δ 3.74 (t, *J* = 5.9 Hz, 6H), 2.80 (t, *J* = 5.8 Hz, 6H), 2.30 (t, *J* = 7.5 Hz, 2H), 1.82 – 1.69 (m, 2H), 0.53 – 0.44 (m, 2H). ^{13}C NMR (101 MHz, Chloroform-*d*) δ 120.98, 57.62, 51.08, 22.02, 20.25, 16.22. ^{29}Si NMR (79 MHz, Chloroform-*d*) δ -69.15. HRMS (ESI): m/z calcd for $C_{10}H_{18}N_2O_3Si^+$, $[M+H]^+$: 243.1159. Found: 243.1163.

1-vinylsilatrane (3k)

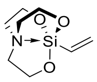

Reaction conditions: r.t., 6h. 90 % yield. ^{1}H NMR (400 MHz, Acetone-d_6) δ 5.85 (dd, *J* = 20.2, 14.3 Hz, 1H), 5.68 – 5.40 (m, 2H), 3.68 (t, *J* = 5.9 Hz, 6H), 2.88 (t, *J* = 5.9 Hz, 6H). ^{13}C NMR (101 MHz, Acetone-d_6) δ 143.29, 126.91, 57.92, 51.47. ^{29}Si NMR (79 MHz, Acetone-d_6) δ -82.82. HRMS (ESI): m/z calcd for $C_8H_{15}NO_3Si^+$, $[M+H]^+$: 202.0894. Found: 202.0886.

1-allylsilatrane (3l)

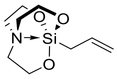

Reaction conditions: r.t., 1h. 94 % yield. ^{1}H NMR (400 MHz, Methanol-d_4) δ 5.98 – 5.82 (m, 1H), 4.76 (ddt, *J* = 17.0, 2.9, 1.6 Hz, 1H), 4.65 (ddt, *J* = 10.0, 2.5, 1.2 Hz, 1H), 3.76 (t, *J* = 5.9 Hz, 6H), 2.91 (t, *J* = 5.9 Hz, 6H), 1.32 (dt, *J* = 7.9, 1.4 Hz, 2H). ^{13}C NMR (101 MHz, Methanol-d_4) δ 140.00, 111.09, 58.53, 51.70, 25.89. ^{29}Si NMR (79 MHz, Methanol-d_4) δ -74.52. HRMS (ESI): m/z calcd for $C_9H_{17}NO_3Si^+$, $[M+Na]^+$: 238.0870. Found: 238.0875.

1-phenylsilatrane (3m)

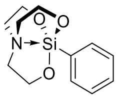

Reaction conditions: r.t., 0.5h. 95 % yield. ^{1}H NMR (400 MHz, Chloroform-*d*) δ 7.84 – 7.67 (m, 2H), 7.38 – 7.20 (m, 3H), 3.91 (t, *J* = 5.9 Hz, 6H), 2.90 (t, *J* = 5.9 Hz, 6H). ^{13}C NMR (101 MHz, Chloroform-*d*) δ 142.16, 134.20, 127.76, 127.33, 57.84, 51.12. ^{29}Si NMR (79 MHz, Chloroform-*d*) δ -80.46. HRMS (ESI): m/z calcd for $C_{12}H_{17}NO_3Si^+$, $[M+Na]^+$: 274.0870. Found: 274.0856.

1-methoxysilatrane (3n)

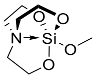

Reaction conditions: r.t., 18h. 99 % yield. ^{1}H NMR (400 MHz, Chloroform-*d*) δ 3.81 (t, *J* = 5.9 Hz, 6H), 3.45 (s, 3H), 2.83 (t, *J* = 5.9 Hz, 6H). ^{13}C NMR (101 MHz, Chloroform-*d*) δ 57.72, 51.29, 51.02. ^{29}Si NMR (79 MHz, Chloroform-*d*) δ -94.55. HRMS (ESI): m/z calcd for $C_7H_{15}NO_4Si^+$, $[M+H]^+$: 206.0843. Found: 206.0842.

1-(3-chloropropyl)-4-methyl-2,8,9-trioxa-5-aza-1-silabicyclo[3.3.3]undecane (3o)

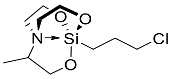

Reaction conditions: 60 °C, 24h. 90 % yield. ^{1}H NMR (400 MHz, Chloroform-*d*) δ 3.85 – 3.71 (m, 4H), 3.70 – 3.62 (m, 1H), 3.47 (t, *J* = 7.2 Hz, 2H), 3.38 (t, *J* = 11.1 Hz, 1H), 3.06 – 2.92 (m, 1H), 2.89 – 2.64 (m, 3H), 2.48 (dd, *J* = 12.3, 3.7 Hz, 1H), 1.91 – 1.80 (m, 2H), 1.08 (d, *J* = 6.6 Hz, 3H), 0.50 – 0.34 (m, 2H). ^{13}C NMR (101 MHz, Chloroform-*d*) δ 63.17, 57.51, 57.49, 53.73, 48.71, 48.09, 45.10, 29.34, 14.51, 10.14. ^{29}Si NMR (79 MHz, Chloroform-*d*) δ -67.89. HRMS (ESI): m/z calcd for $C_{10}H_{20}ClNO_3Si^+$, $[M+H]^+$: 266.0974. Found: 266.0969.

1-(3-chloropropyl)-3,3-dimethyl-2,8,9-trioxa-5-aza-1-silabicyclo[3.3.3]undecane (3p)

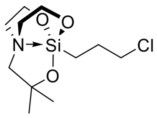

Reaction conditions, 60 °C, 24h. 75 % yield. ^{1}H NMR (400 MHz, Chloroform-*d*) δ 3.80 – 3.67 (m, 4H), 3.50 (t, *J* = 7.6 Hz, 2H), 2.97 – 2.76 (m, 4H), 2.67 (s, 2H), 1.97 – 1.78 (m, 2H), 1.26 (s, 6H), 0.54 – 0.33 (m, 2H). ^{13}C NMR (101 MHz, Chloroform-*d*) δ 70.35, 62.74, 58.28, 55.11, 48.71, 31.23, 29.41, 14.58. ^{29}Si NMR (79 MHz, Chloroform-*d*) δ -68.72. HRMS (ESI): m/z calcd for $C_{11}H_{22}ClNO_3Si^+$, $[M+H]^+$: 280.1130. Found: 280.1109.

1-(1-(3-chloropropyl)-2,8,9-trioxa-5-aza-1-silabicyclo[3.3.3]undecan-3-yl)ethan-1-ol (3q), mixture of regioisomers

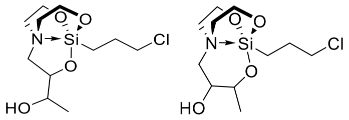

Reaction conditions: 60° C, 24h. 81 % yield. ^{13}C NMR (101 MHz, Chloroform-*d*) δ 71.78, 70.74, 69.11, 68.44, 57.66, 57.57, 57.55, 52.62, 52.28, 52.22, 51.51, 51.42, 50.16, 48.71, 29.41, 29.31, 19.35, 17.22, 14.45, 14.17. ^{29}Si NMR (79 MHz, Chloroform-*d*) δ -68.98, -69.95. HRMS (ESI): m/z calcd for $C_{11}H_{22}ClNO_4Si^+$, $[M+H]^+$: 296.1079. Found: 296.1066.

1-(3-chloropropyl)-3,7,10-trimethyl-2,8,9-trioxa-5-aza-1-silabicyclo[3.3.3]undecane (3r), mixture of diastereoisomers

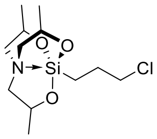

Reaction conditions: 60 °C, 12h. washing with hexane was performed at 0 °C. 70 % yield. ^{1}H NMR (400 MHz, Chloroform-*d*) δ 4.18 – 3.73 (m, 3H), 3.58 – 3.44 (m, 2H), 3.11 – 2.13 (m, 6H), 1.98 – 1.81 (m, 2H), 1.32 – 1.05 (m, 9H), 0.53 – 0.36 (m, 2H). ^{13}C NMR (101 MHz, Chloroform-*d*) δ 66.81, 65.25, 65.06, 64.96, 63.40, 61.81, 59.01, 48.76, 48.70, 29.55, 29.45, 23.37, 20.83, 20.57, 20.47, 14.52, 14.46. ^{29}Si NMR (79 MHz, Chloroform-*d*) δ -68.03, -70.43. HRMS (ESI): m/z calcd for $C_{12}H_{24}ClNO_3Si^+$, $[M+H]^+$: 294.1287. Found: 294.1286.

1-(3-chloropropyl)-3-phenyl-2,8,9-trioxa-5-aza-1-silabicyclo[3.3.3]undecane (3s)

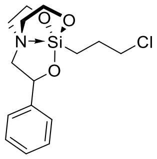

Reaction conditions: r.t., 24h. 98 % yield. ^{1}H NMR (400 MHz, Acetonitrile-d_3) δ 7.59 – 7.12 (m, 5H), 4.84 (dd, *J* = 10.9, 4.5 Hz, 1H), 3.79 – 3.67 (m, 4H), 3.53 (t, *J* = 7.2 Hz, 2H), 3.11 (dd, *J* = 12.5, 4.5 Hz, 1H), 3.07 – 2.88 (m, 2H), 2.81 (td, *J* = 6.7, 6.1, 1.6 Hz, 2H), 2.45 (dd, *J* = 12.5, 10.9 Hz, 1H), 1.89 – 1.78 (m, 2H), 0.45 – 0.33 (m, 2H). ^{13}C NMR (101 MHz, Acetonitrile-d_3) δ 143.43, 129.36, 128.45, 126.48, 70.00, 58.39, 58.09, 52.15, 51.65, 49.76, 30.47, 15.61. ^{29}Si NMR (79 MHz, Acetonitrile-d_3) δ -71.44. HRMS (ESI): m/z calcd for $C_{15}H_{22}ClNO_3Si^+$, $[M+H]^+$: 328.1130. Found: 328.1121.

5. Large-scale synthesis of 3k

The reaction was conducted following the general procedure with reagents shown in Table S1, but in aerobic conditions. The initial biphasic mixture became homogeneous during reaction (Figure S2 and Figure S3), and then copious amount of white crystalline product precipitated. A water bath is recommended to absorb the heat of crystallization during reaction. The isolation yield was 99 %. After washing the crude product, the organic phase was combined and concentrated to afford 0.925 g of recovered catalyst, whose ^{1}H NMR spectrum is presented in Figure S1.

Table S1. Production cost calculation of **3k**.

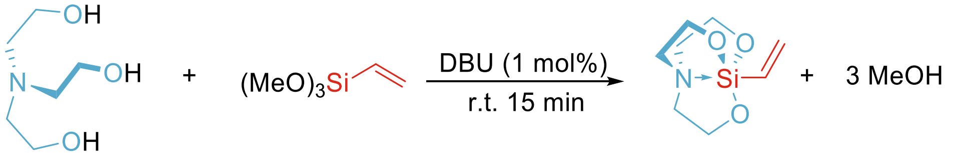

	1a	2k		3k	
**Mass:**	91.5 g	91.8 g	0.934 g	121 g	58 g
**Moles:**	607 mmol	607 mmol	6.07 mmol	601 mmol	1810 mmol
**GMW:**	149.19	148.23	152.24	201.28	32.04

**Material**	Price of package[a] (EUR)	Used for reaction	Price of portion (EUR)
**Triethanolamine, 99%**	46.11/1L	81 ml	3.73
**Vinyltrimethoxysilane, 98%**	195.52/1L	94.5 ml	18.47
**DBU, 99%**	211.40/500 g	0.934 g	0.39
**Hexane, fraction from petroleum**	8.53/1L	500 ml	4.26
		**Total:**	26.85
			(2.22 per 10 g)

[a]Prices retrieved from the CHEMAT online catalogue (chemat.com.pl) in November 2023.

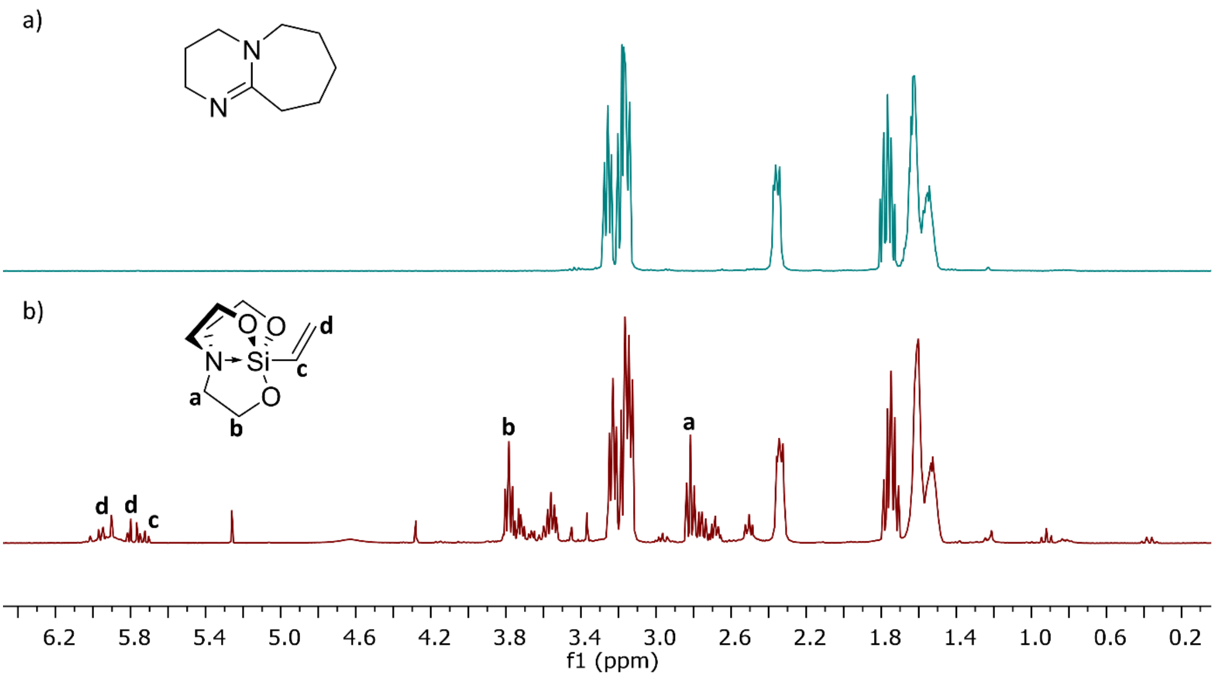

Figure S1. ^{1}H NMR spectrum of a) DBU, b) recovered catalyst (300 MHz, $CDCl_3$).

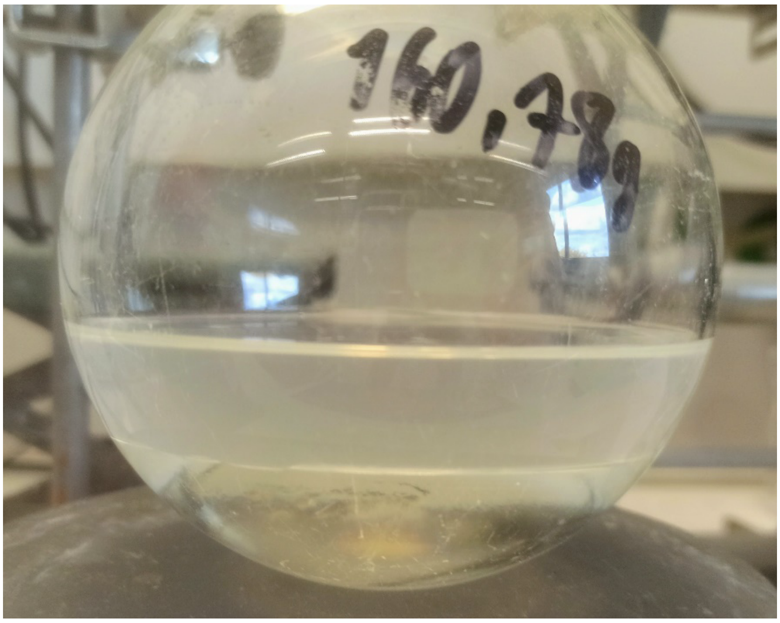

Figure S2. Immiscible mixture of triethanolamine with vinyltrimethoxysilane (before the addition of DBU).

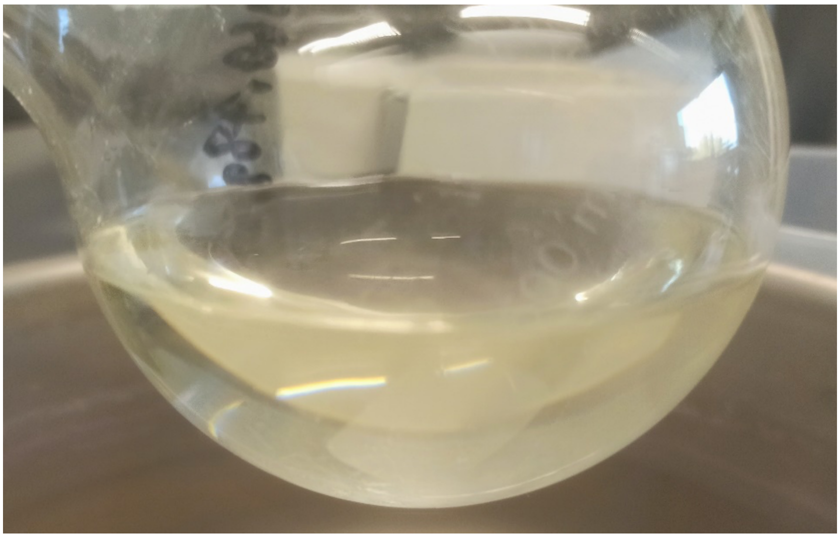

Figure S3. Homogeneous mid-reaction mixture of triethanolamine, vinyltrimethoxysilane and DBU (approximately after 8 minutes of mixing).

6. Catalyst regeneration

3k was synthesized by reaction of **1a** (1.00 eq., 12 mmol), vinyltrimethoxysilane (1.03 eq.) and DBU (0.01 eq.) in aerobic conditions (Cycle 1 in Figure 9). The liquid organic phase after the usual work-up of the product was placed in a new round-bottomed flask and then concentrated *in vacuo*. To this flask, a new portion of **1a** and vinyltrimethoxysilane was added and reaction was carried out (Cycle 2 in Figure 9). After full conversion of the substrates, confirmed by GC, dry, crude **3k** was washed with hexane and the whole process was repeated until finishing Cycle 5.

7. NMR spectra

Figure S4. ^{1}H NMR spectrum of **3a** (400 MHz, CD_3OD).

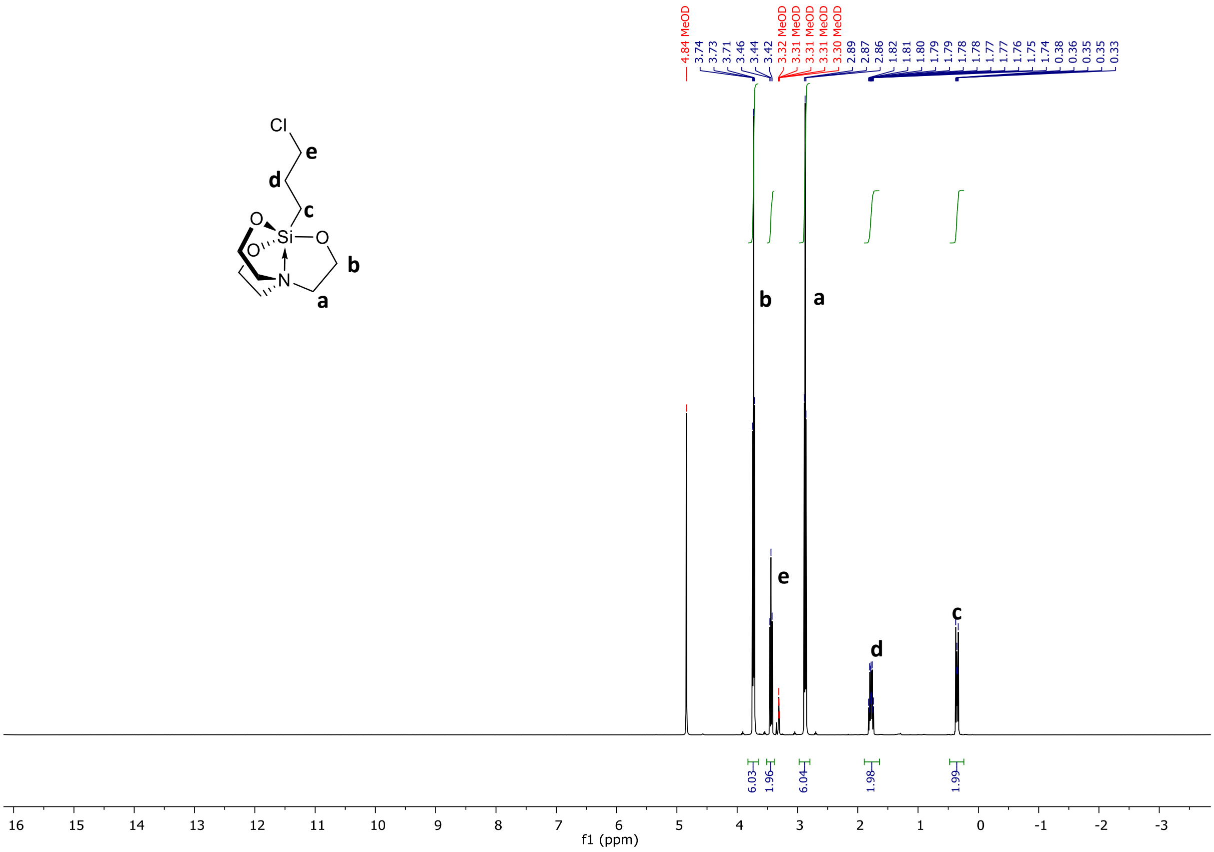

Figure S5. ^{13}C NMR spectrum of **3a** (101 MHz, CD_3OD).

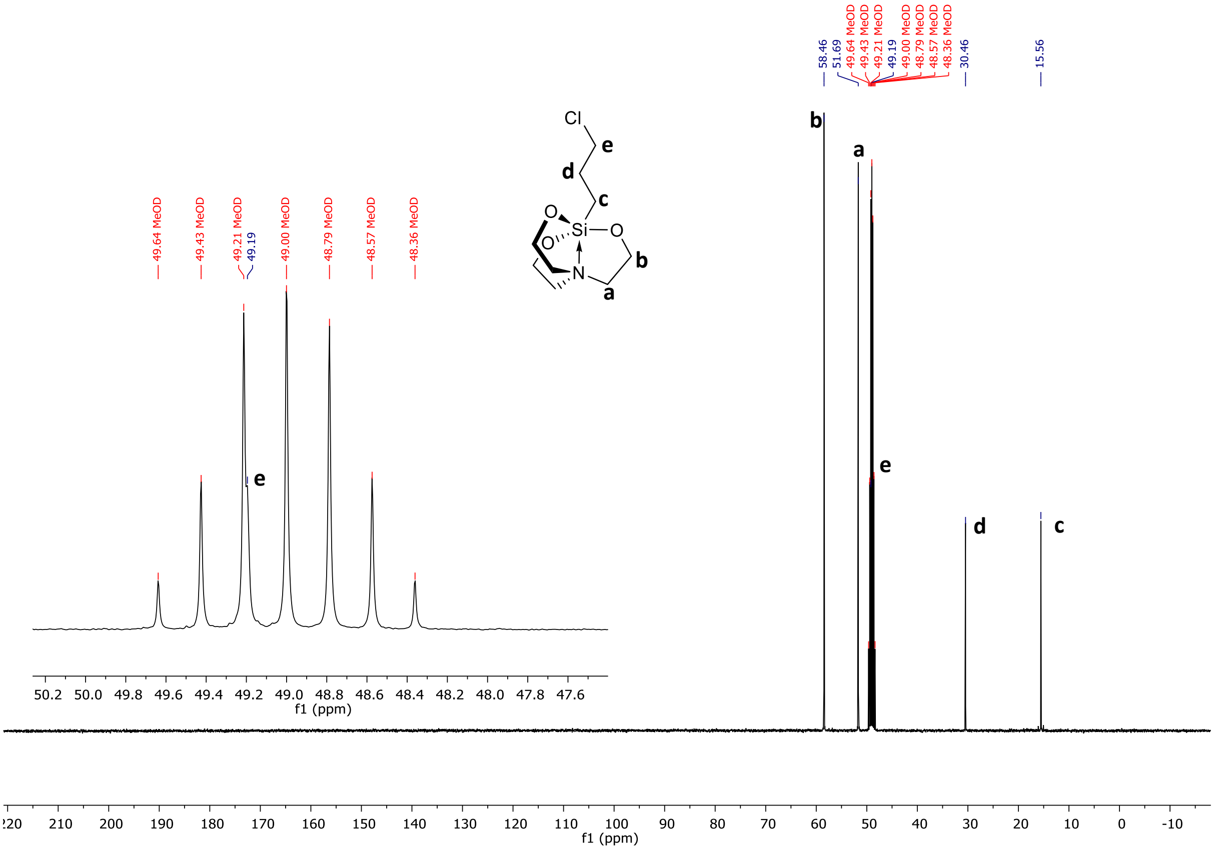

Figure S6. ^{29}Si NMR spectrum of **3a** (79 MHz, CD_3OD).

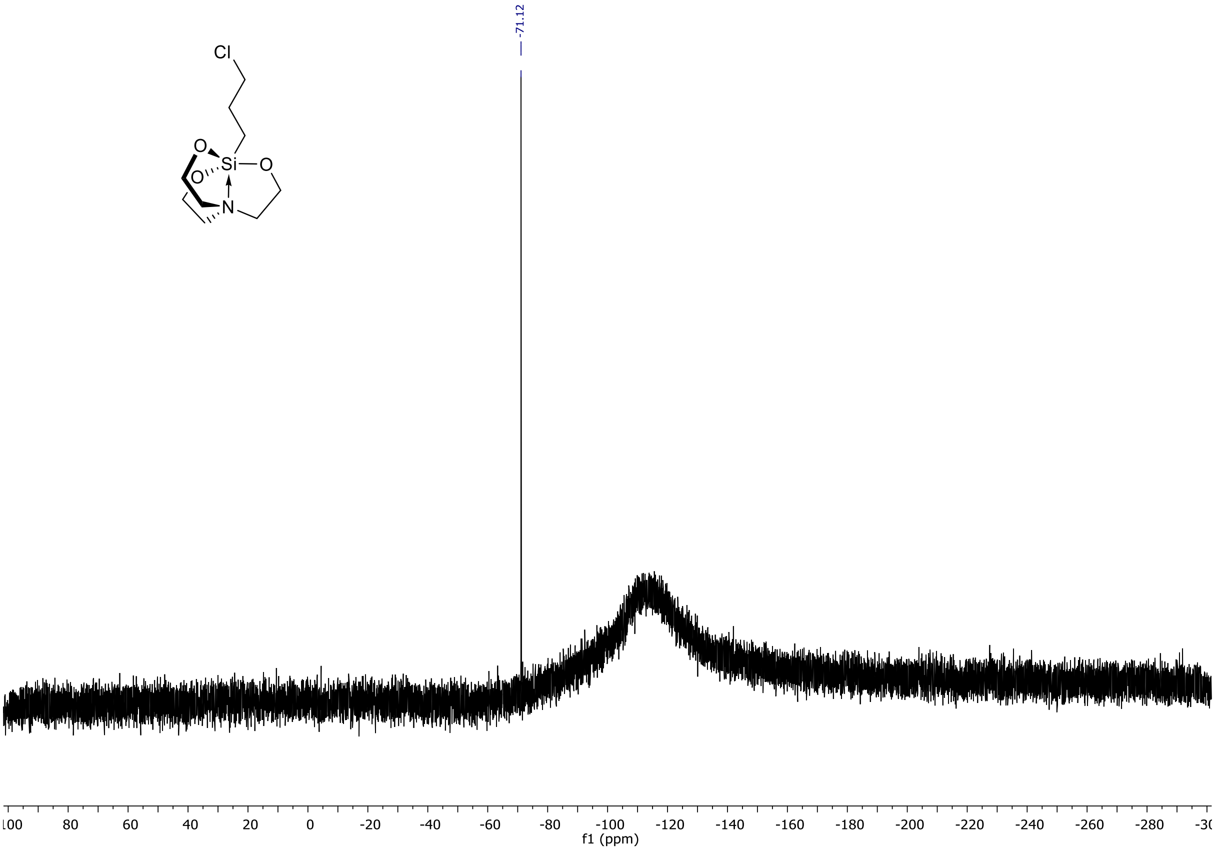

Figure S7. ^{1}H NMR spectrum of **3b** (400 MHz, $CDCl_3$).

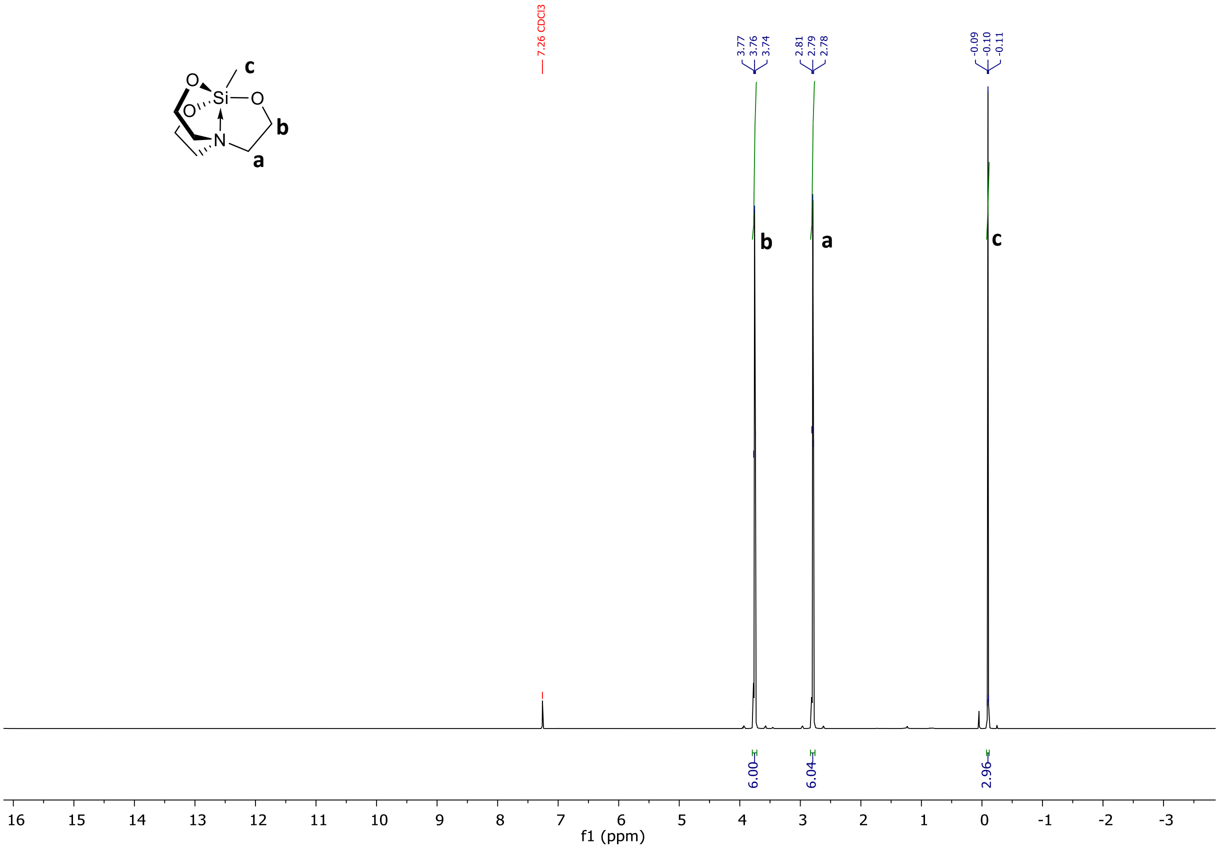

Figure S8. ^{13}C NMR spectrum of **3b** (101 MHz, $CDCl_3$).

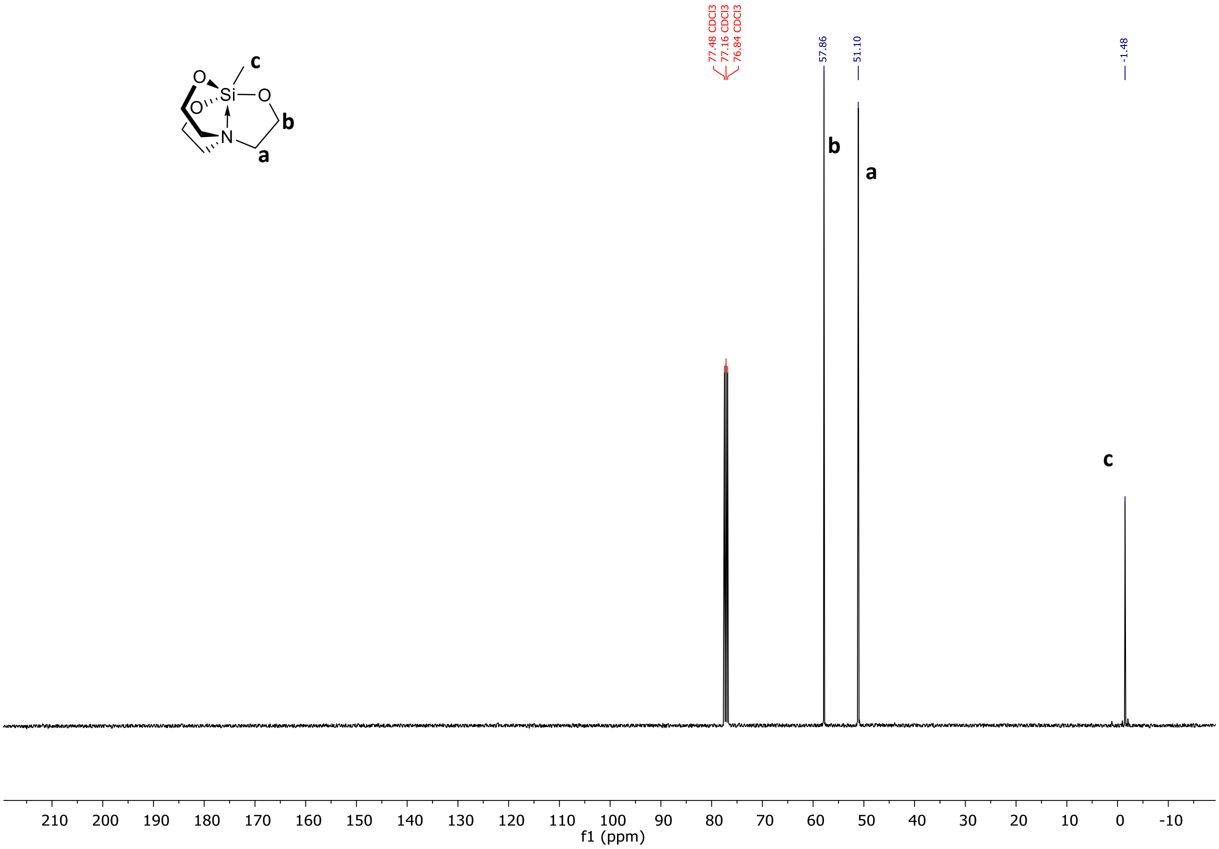

Figure S9. ^{29}Si NMR spectrum of **3b** (79 MHz, $CDCl_3$).

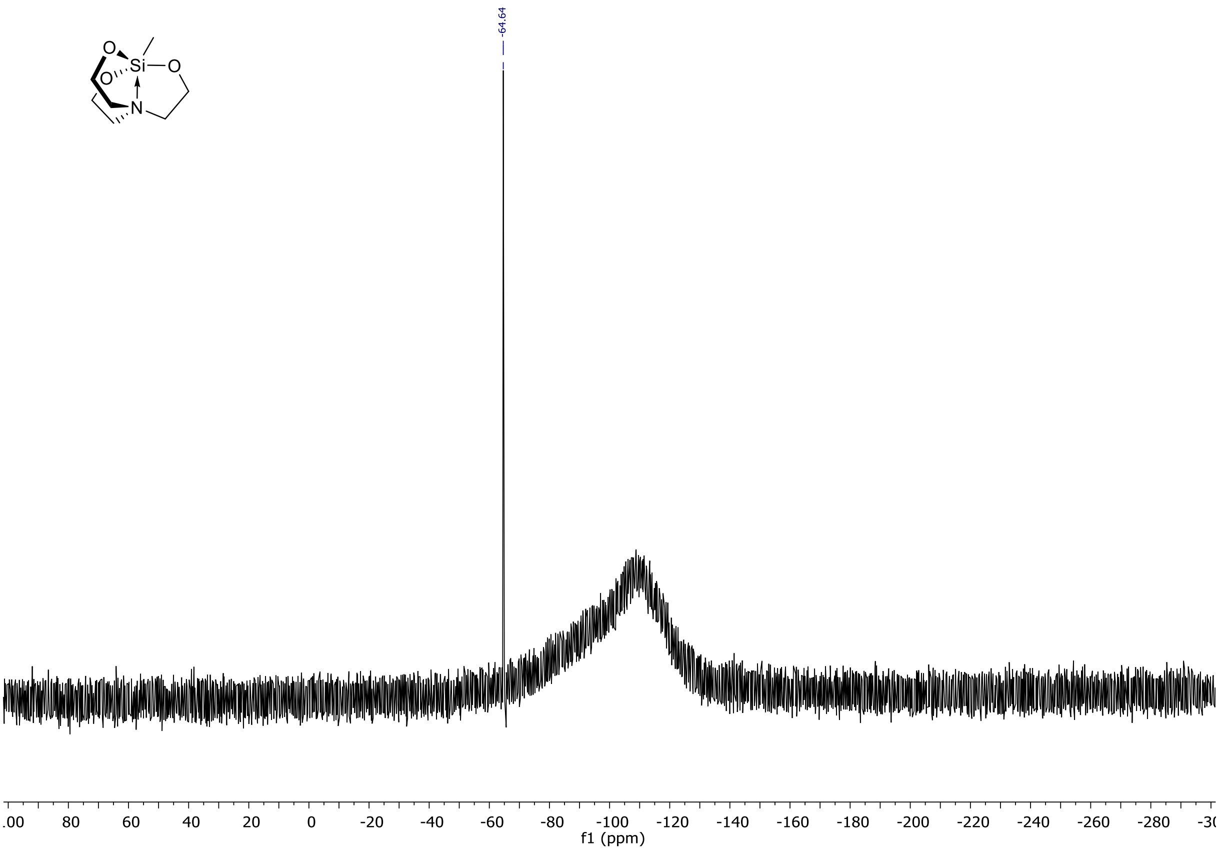

Figure S10. ^{1}H NMR spectrum of **3c** (400 MHz, $CDCl_3$).

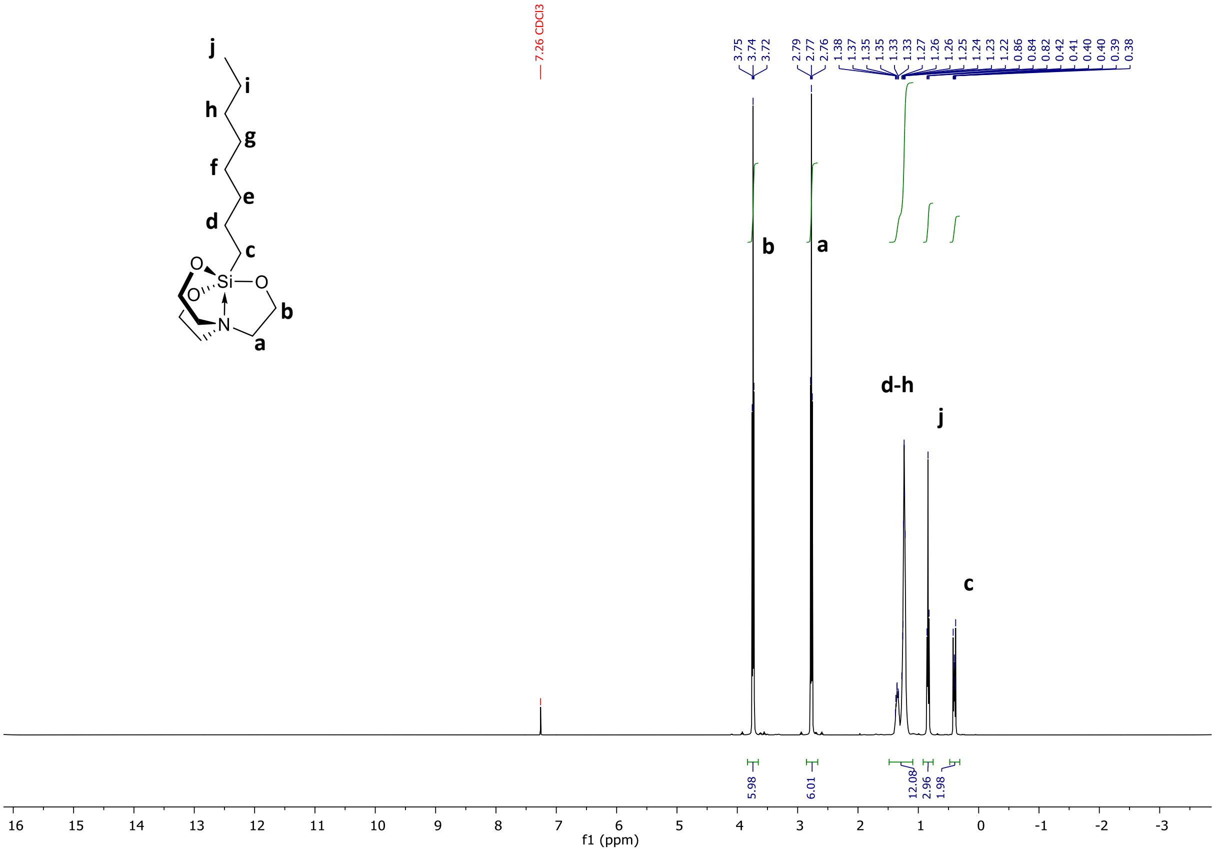

Figure S11. ^{13}C NMR spectrum of **3c** (101 MHz, $CDCl_3$).

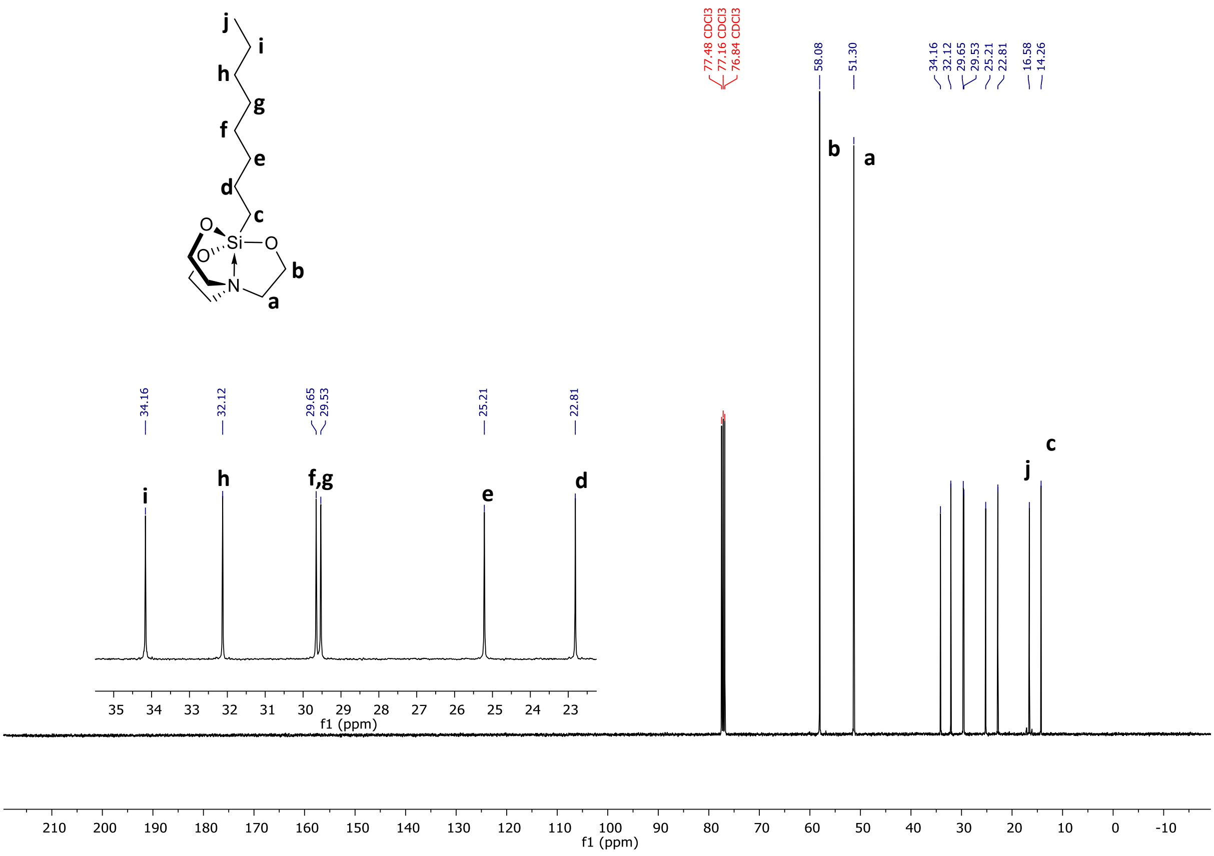

Figure S12. ^{29}Si NMR spectrum of **3c** (79 MHz, $CDCl_3$).

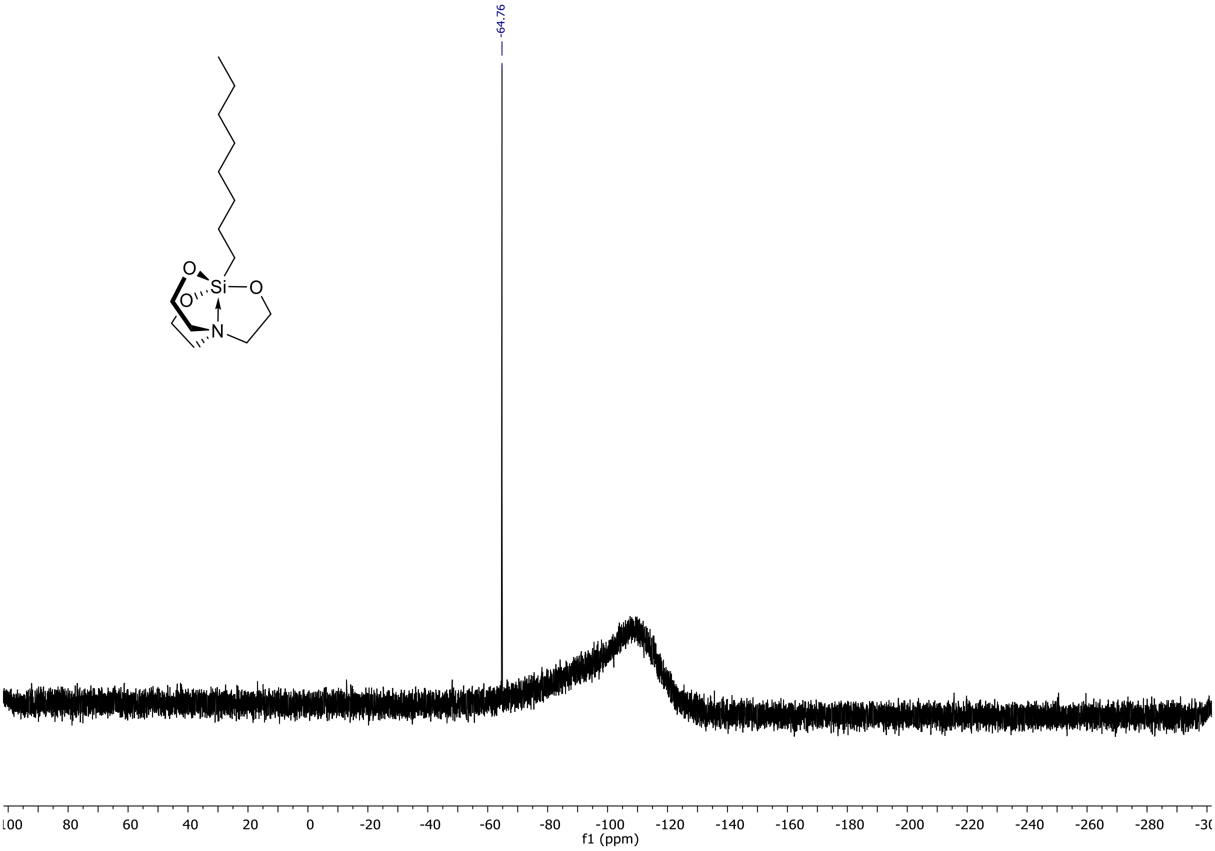

Figure S13. ^{1}H NMR spectrum of **3d** (400 MHz, $CDCl_3$).

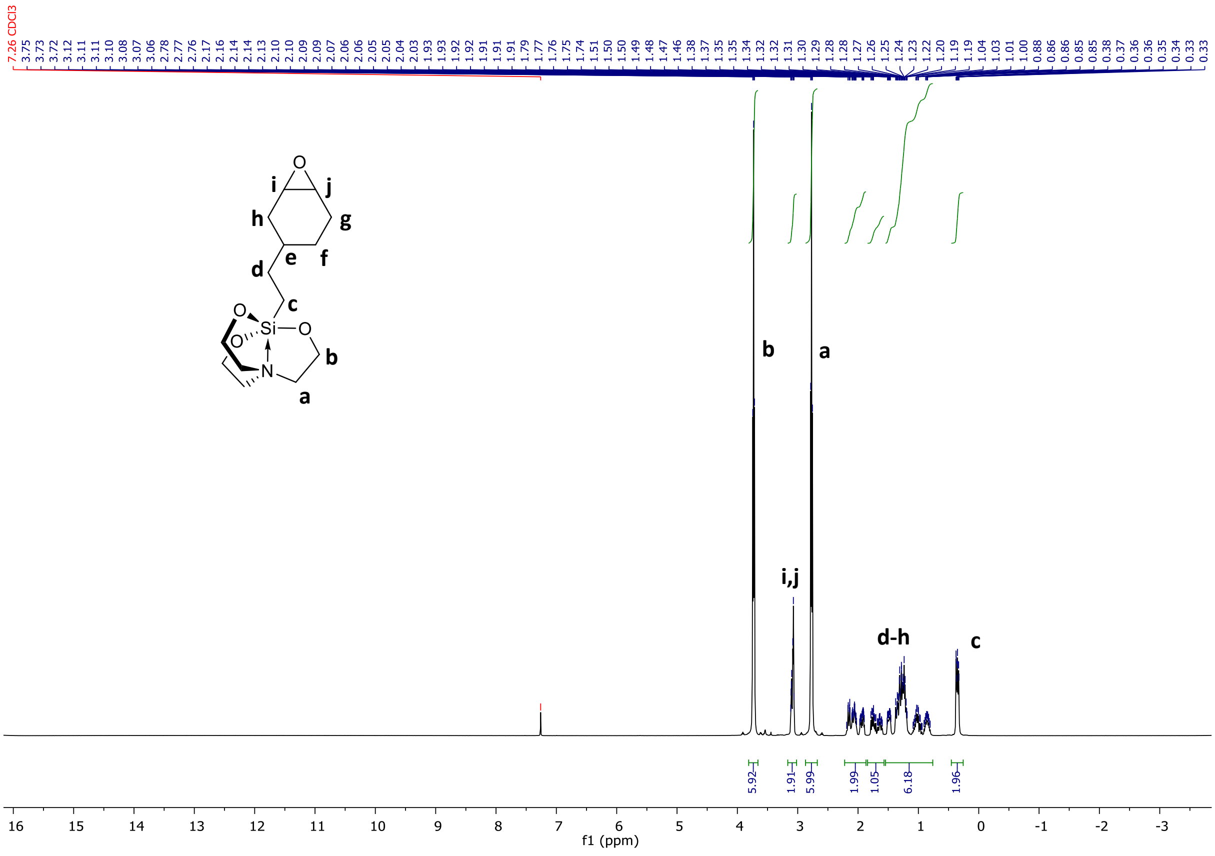

Figure S14. ^{13}C NMR spectrum of **3d** (101 MHz, $CDCl_3$).

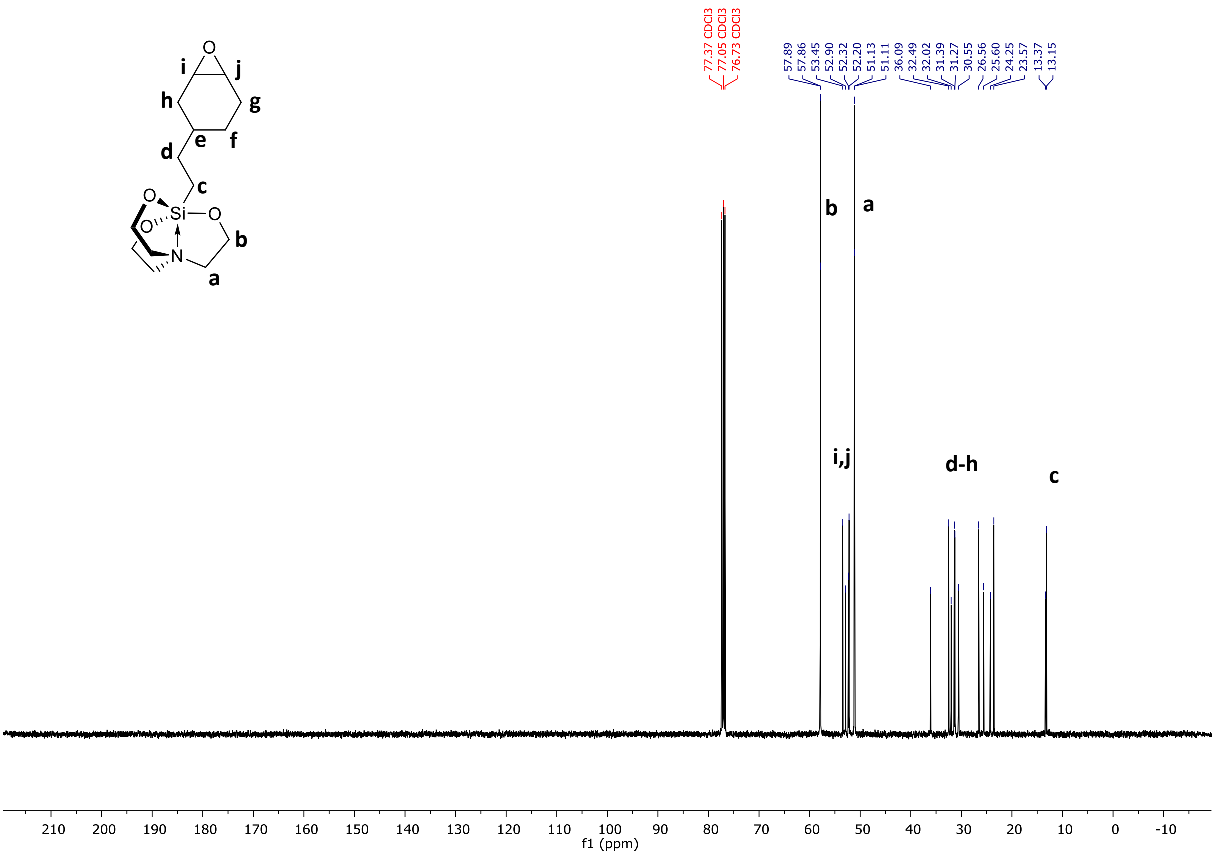

Figure S15. ^{29}Si NMR spectrum of **3d** (79 MHz, $CDCl_3$).

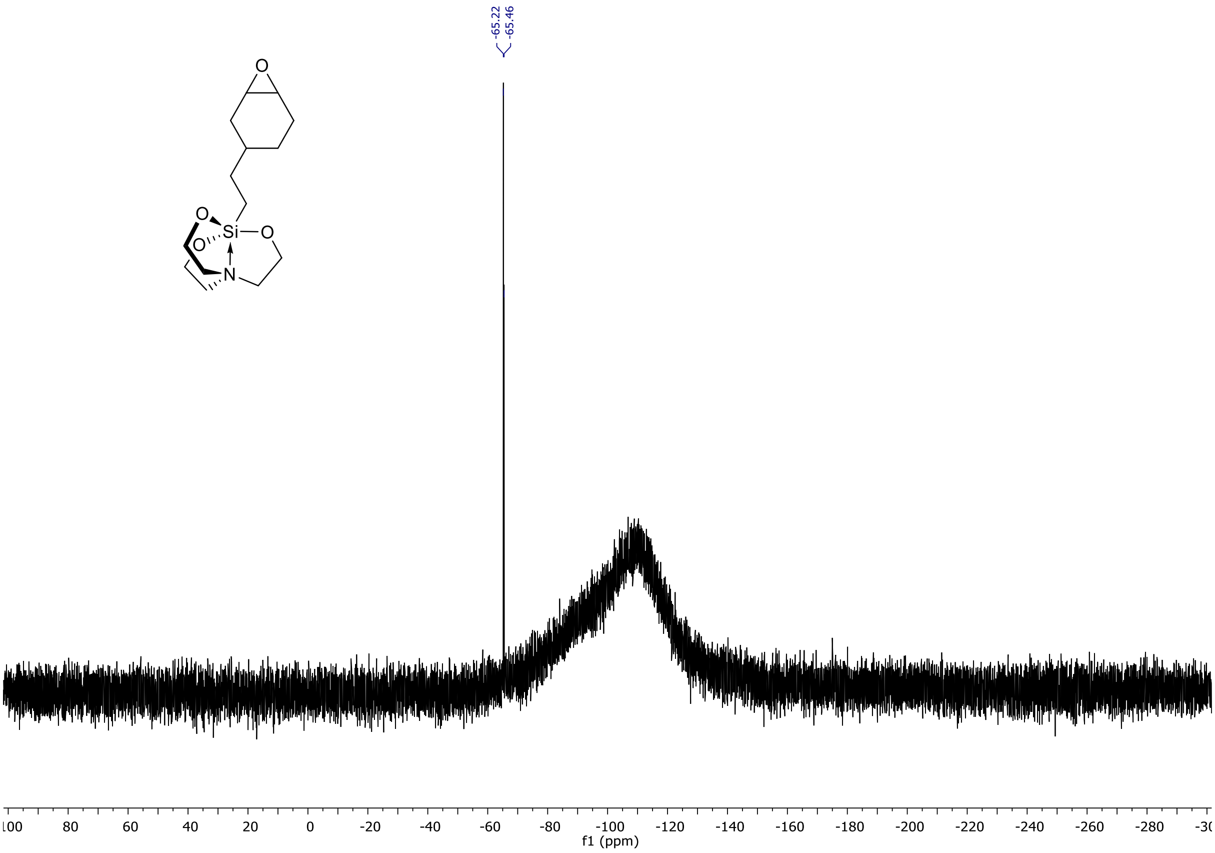

Figure S16. ^{1}H NMR spectrum of **3e** (400 MHz, CD_3OD).

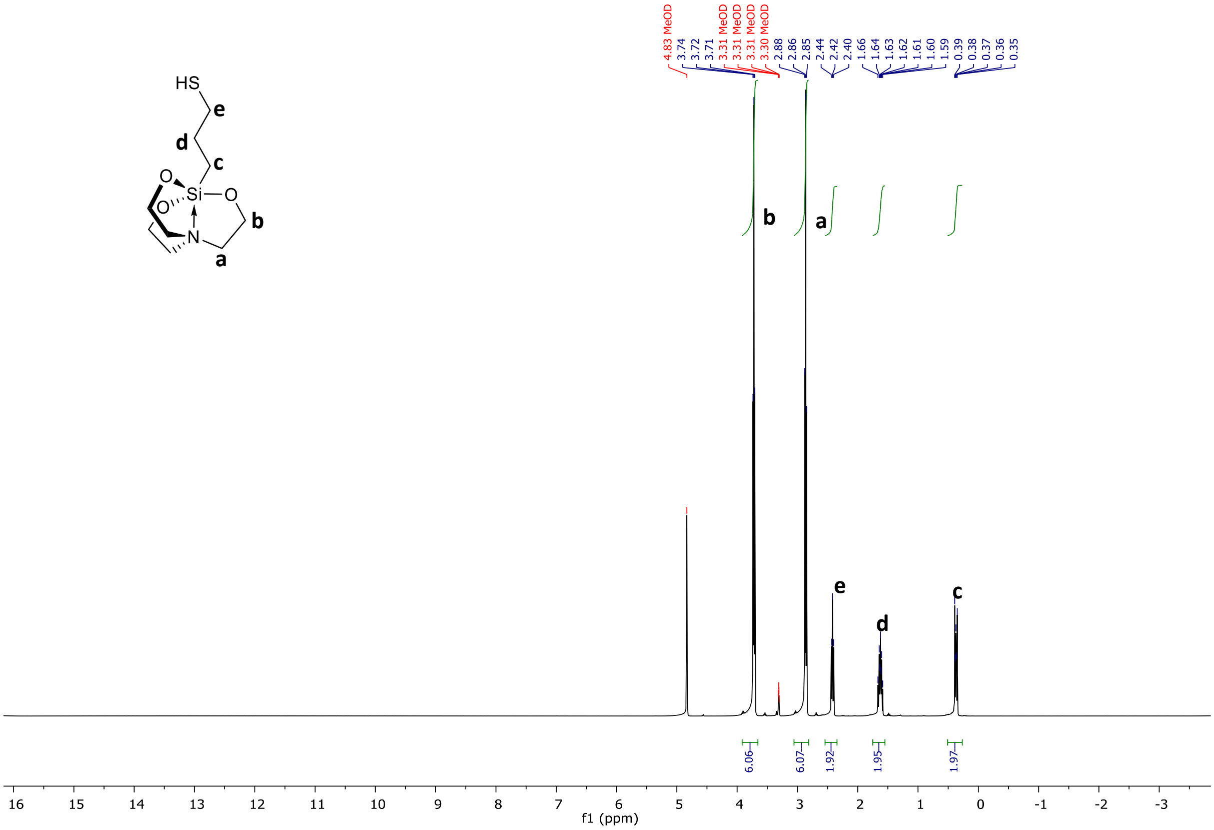

Figure S17. ^{13}C NMR spectrum of **3e** (101 MHz, CD_3OD).

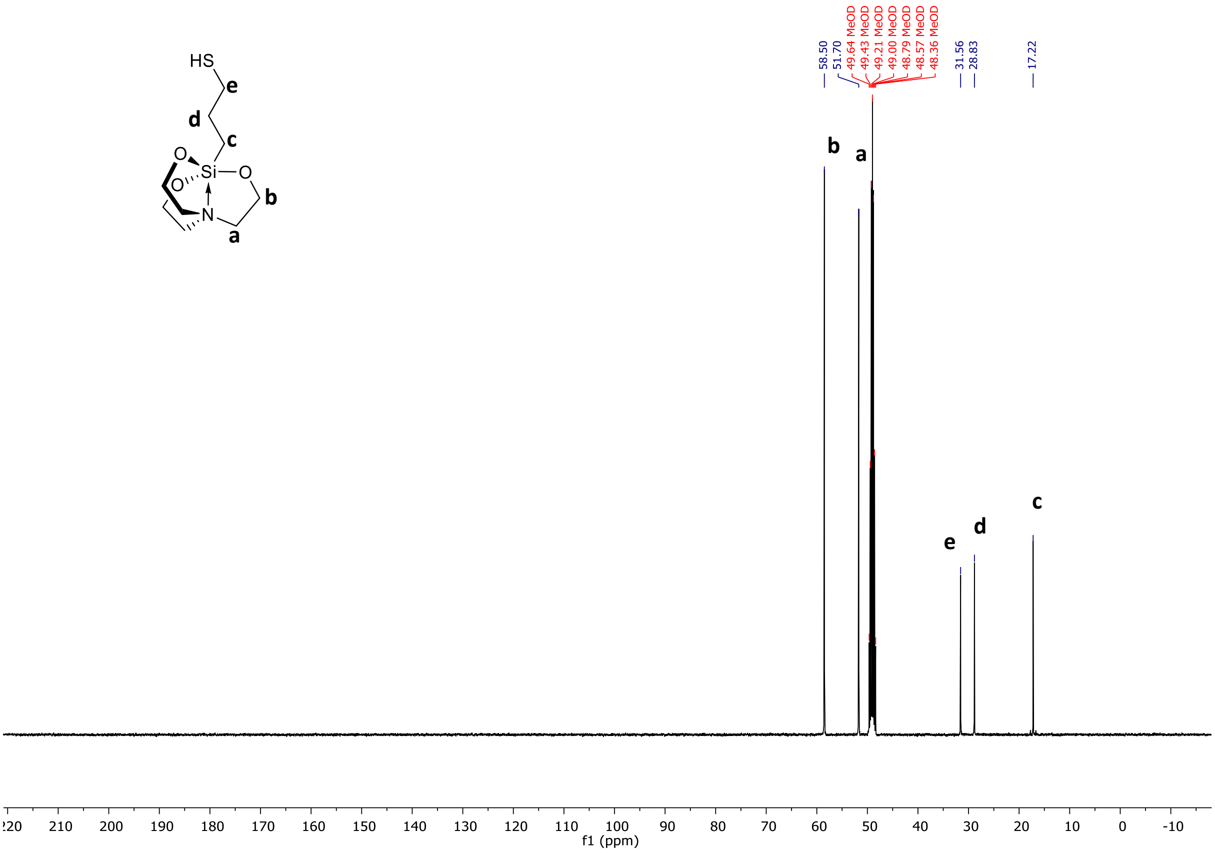

Figure S18. ^{29}Si NMR spectrum of **3e** (79 MHz, CD_3OD).

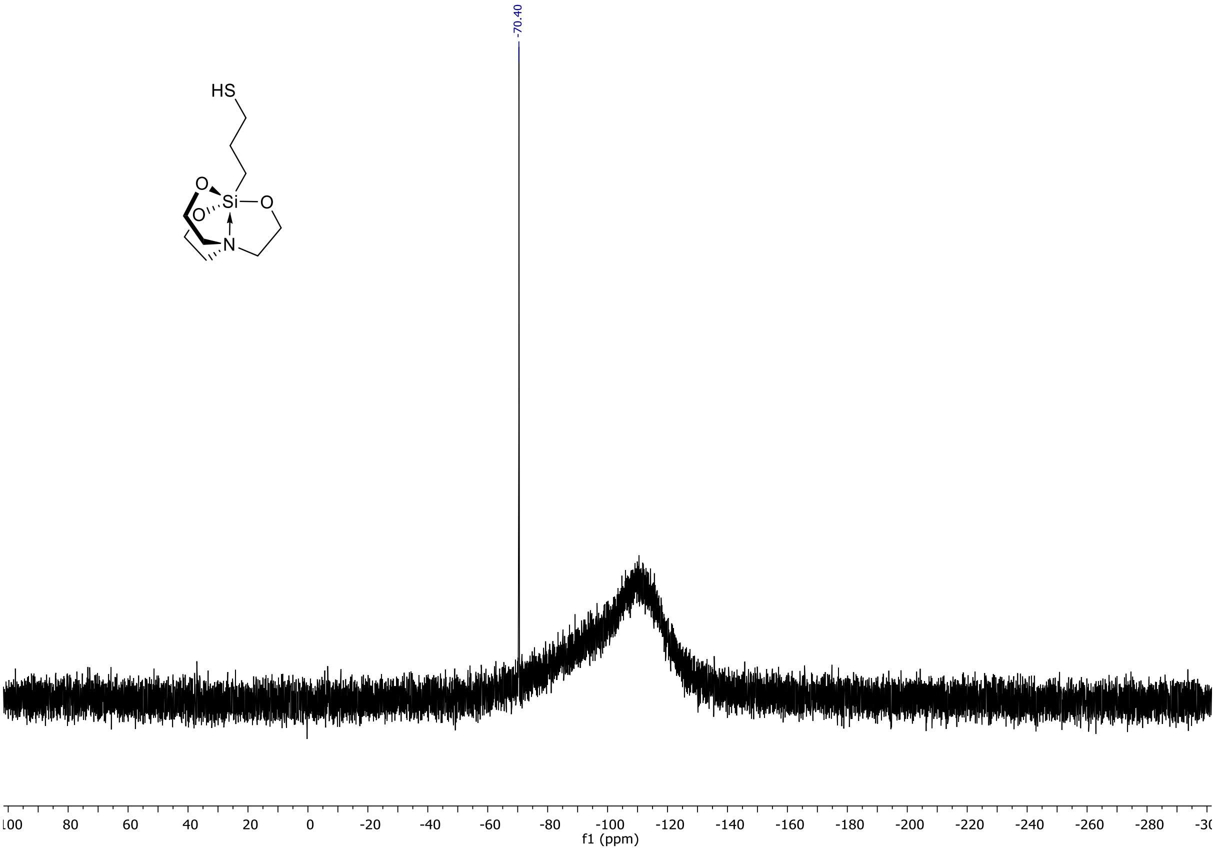

Figure S19. ^{1}H NMR spectrum of **3f** (400 MHz, $CDCl_3$).

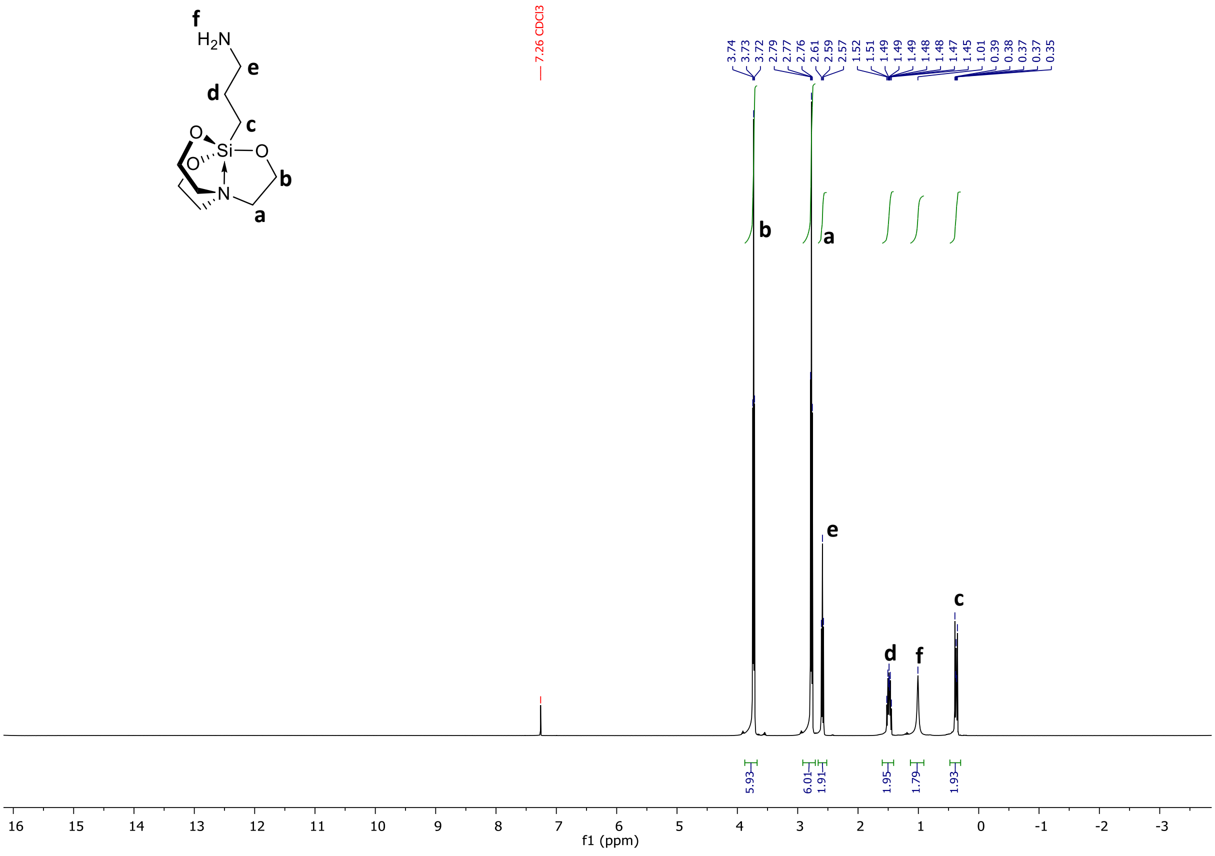

Figure S20. ^{13}C NMR spectrum of **3f** (101 MHz, $CDCl_3$).

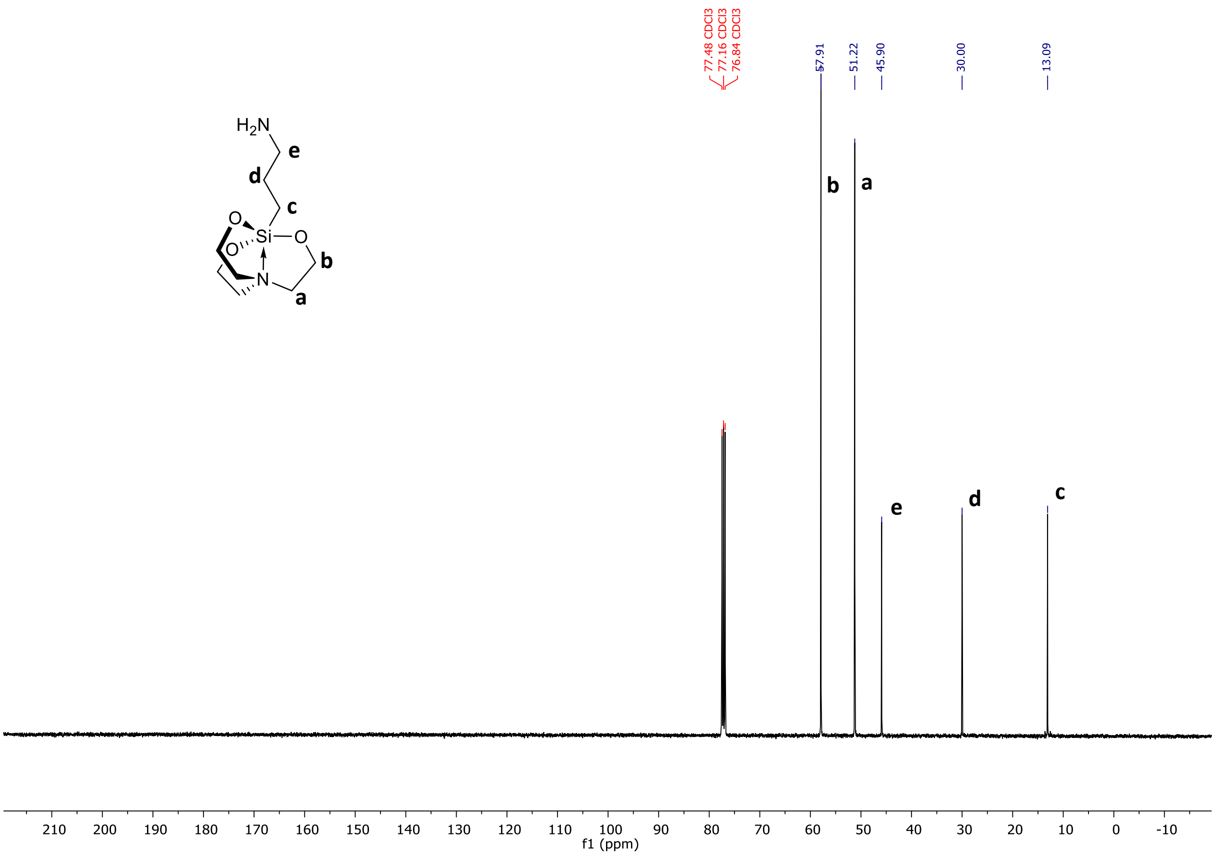

Figure S21. ^{29}Si NMR spectrum of **3f** (79 MHz, $CDCl_3$).

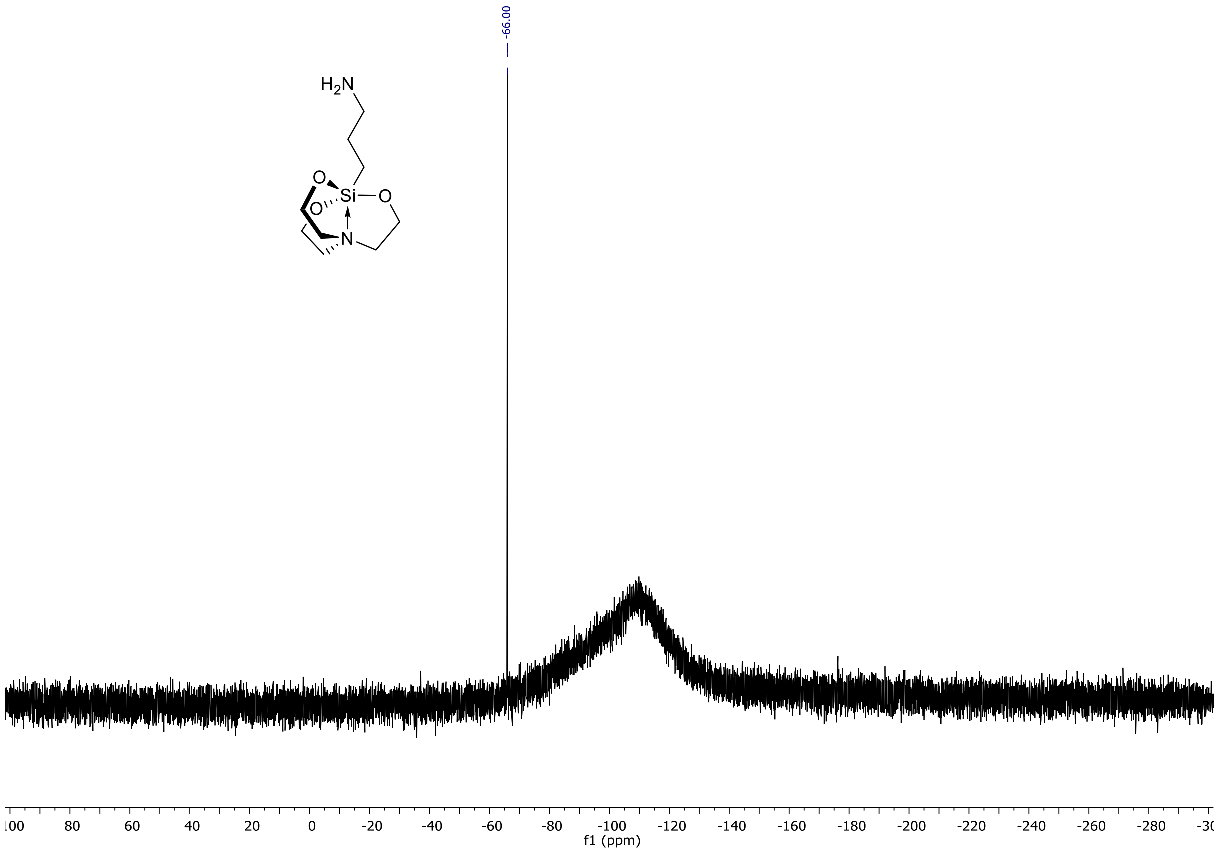

Figure S22. ^{1}H NMR spectrum of **3g** (400 MHz, CD_3OD).

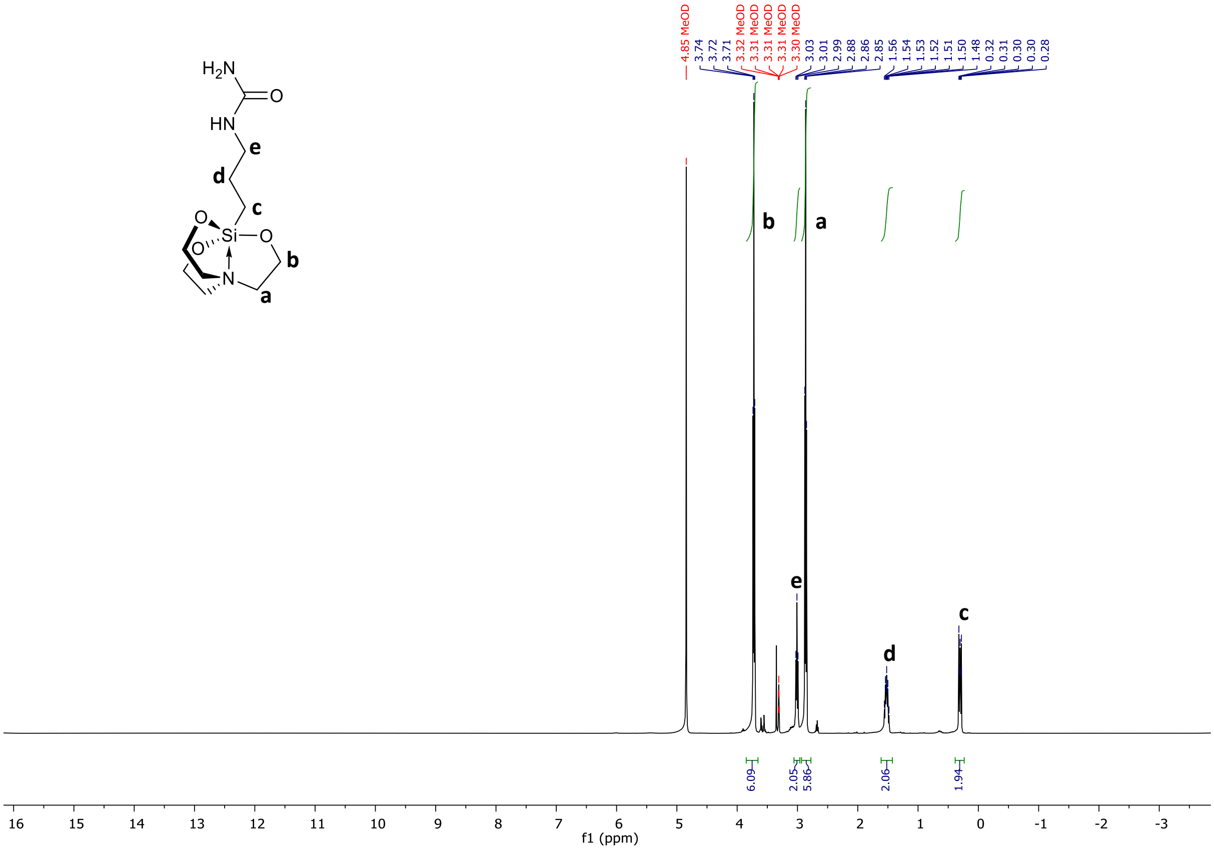

Figure S23. ^{13}C NMR spectrum of **3g** (101 MHz, CD_3OD).

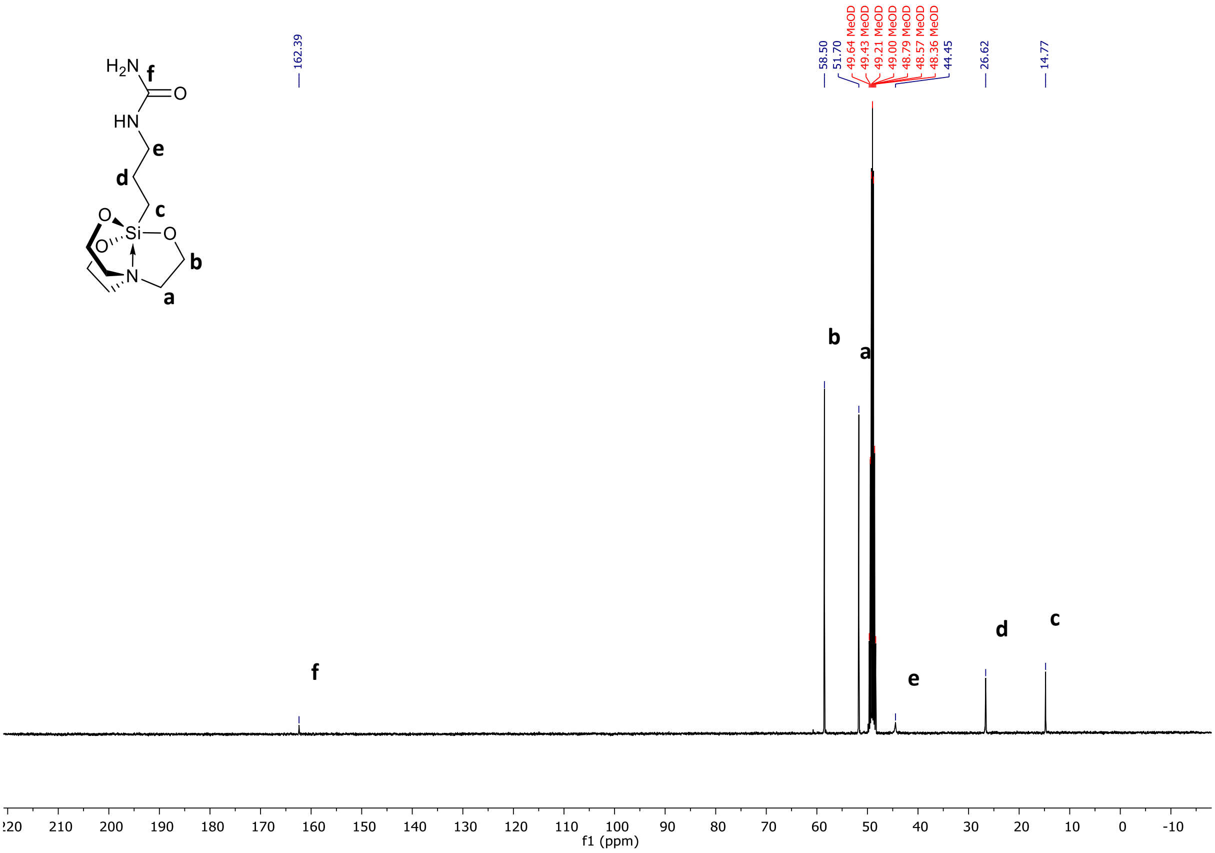

Figure S24. ^{29}Si NMR spectrum of **3g** (79 MHz, CD_3OD).

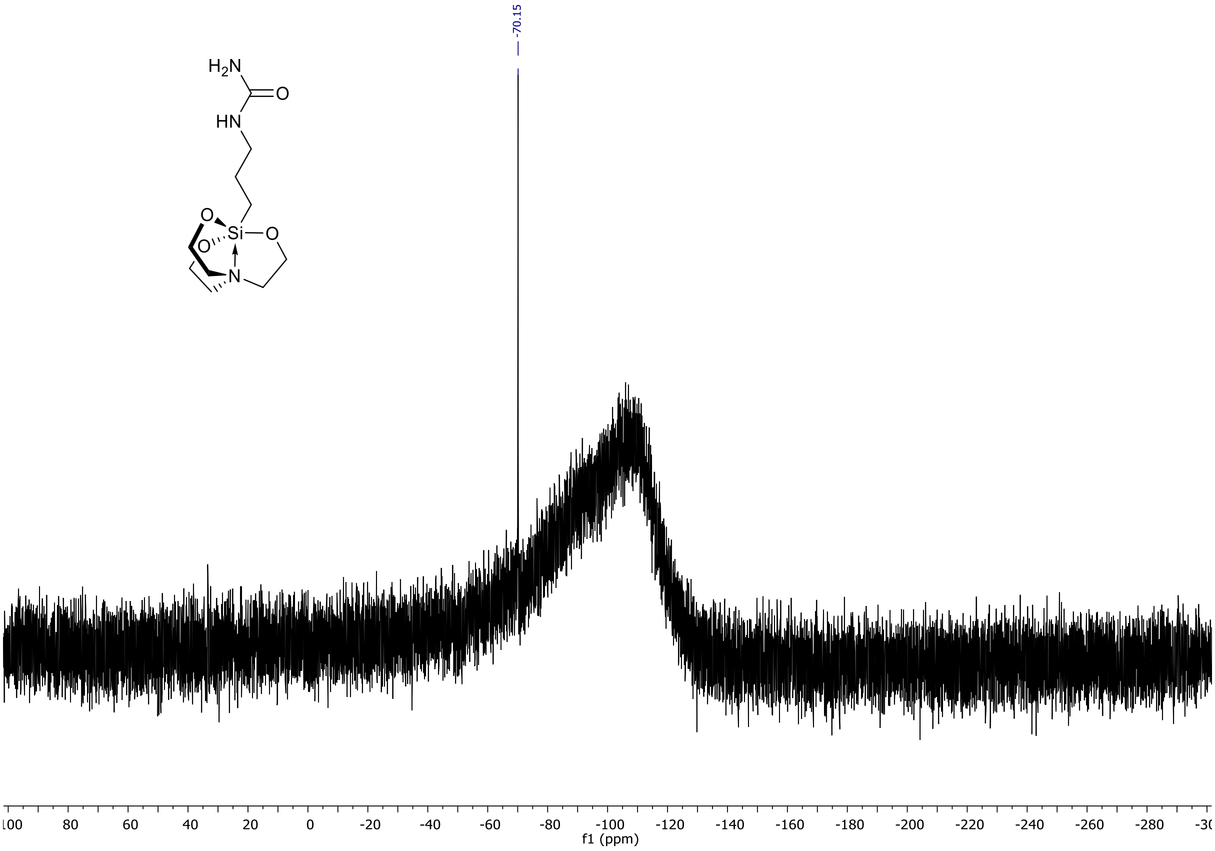

Figure S25. ^{1}H NMR spectrum of **3h** (400 MHz, $CDCl_3$).

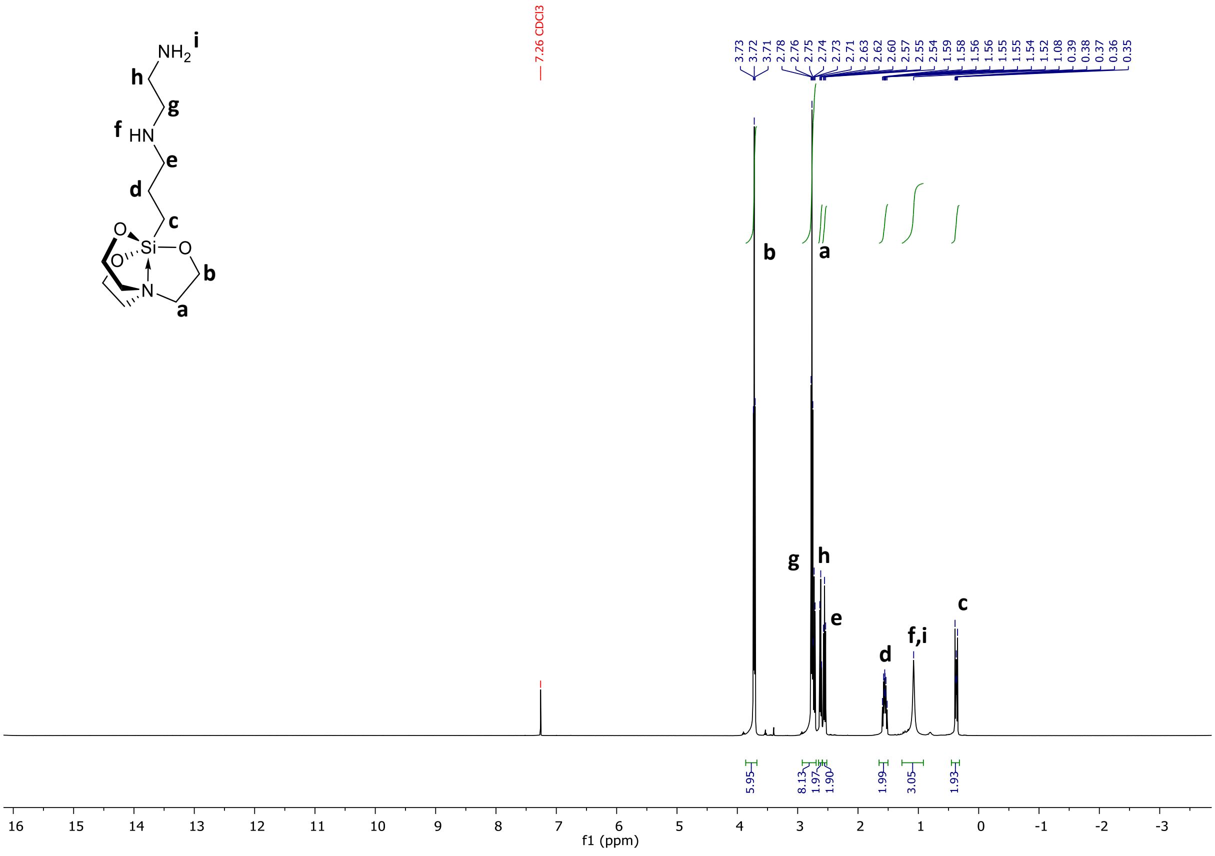

Figure S26. ^{13}C NMR spectrum of **3h** (101 MHz, $CDCl_3$).

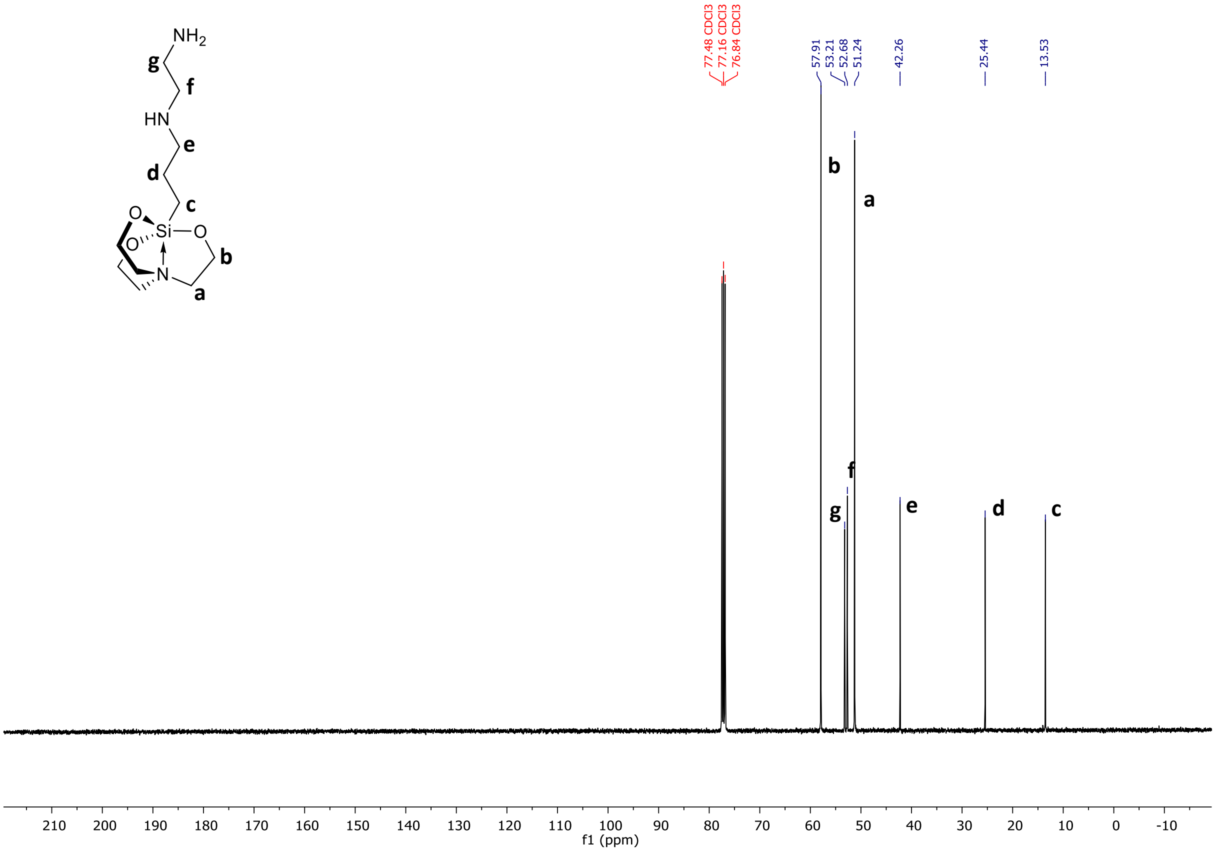

Figure S27. ^{29}Si NMR spectrum of **3h** (79 MHz, $CDCl_3$).

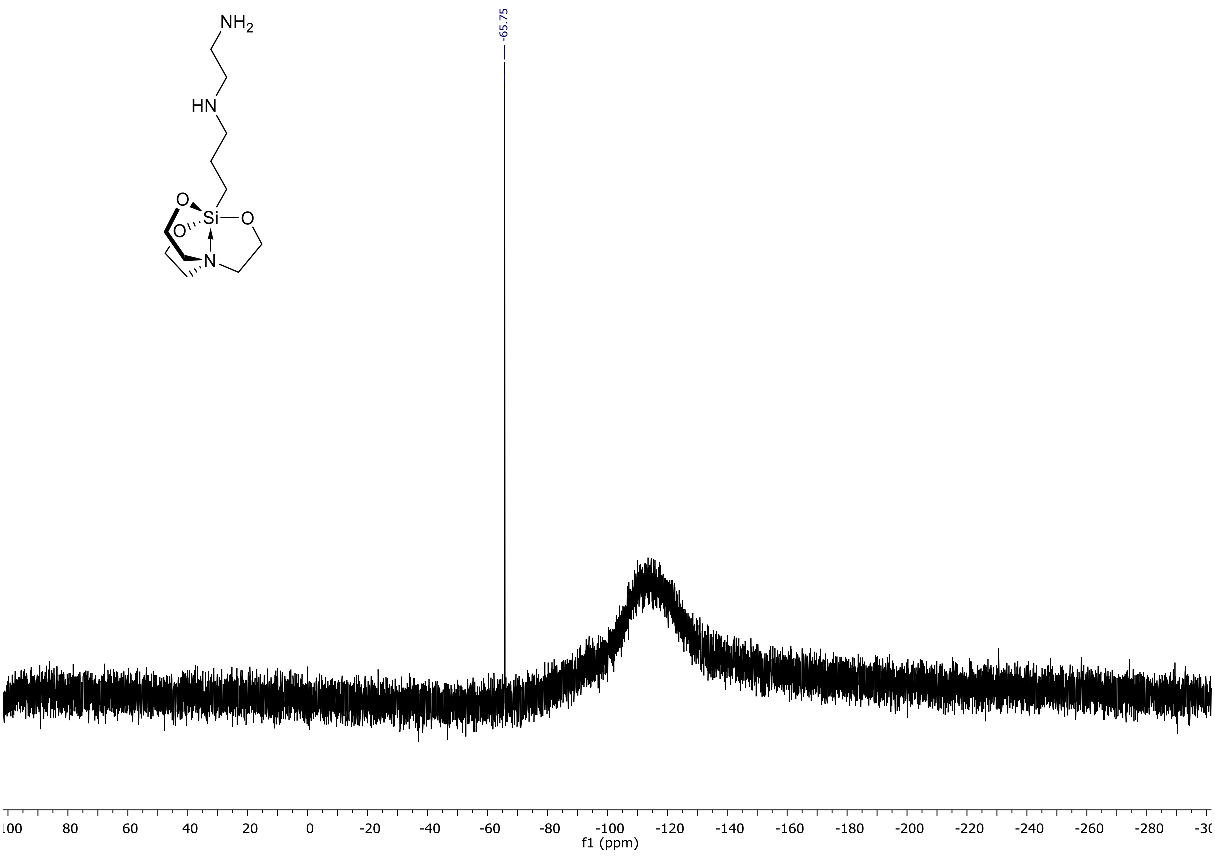

Figure S28. ^{1}H NMR spectrum of **3i** (400 MHz, $(CD_3)_2CO$).

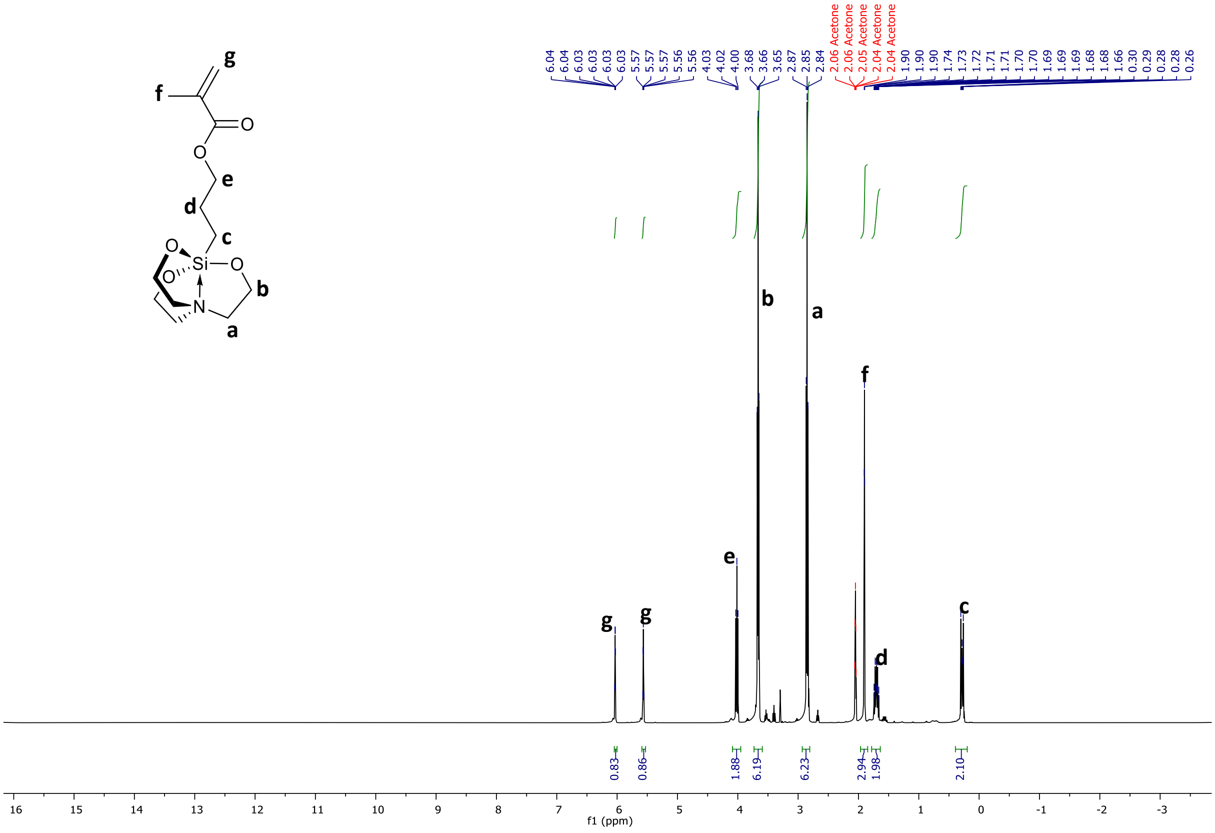

Figure S29. ^{13}C NMR spectrum of **3i** (101 MHz, $(CD_3)_2CO$).

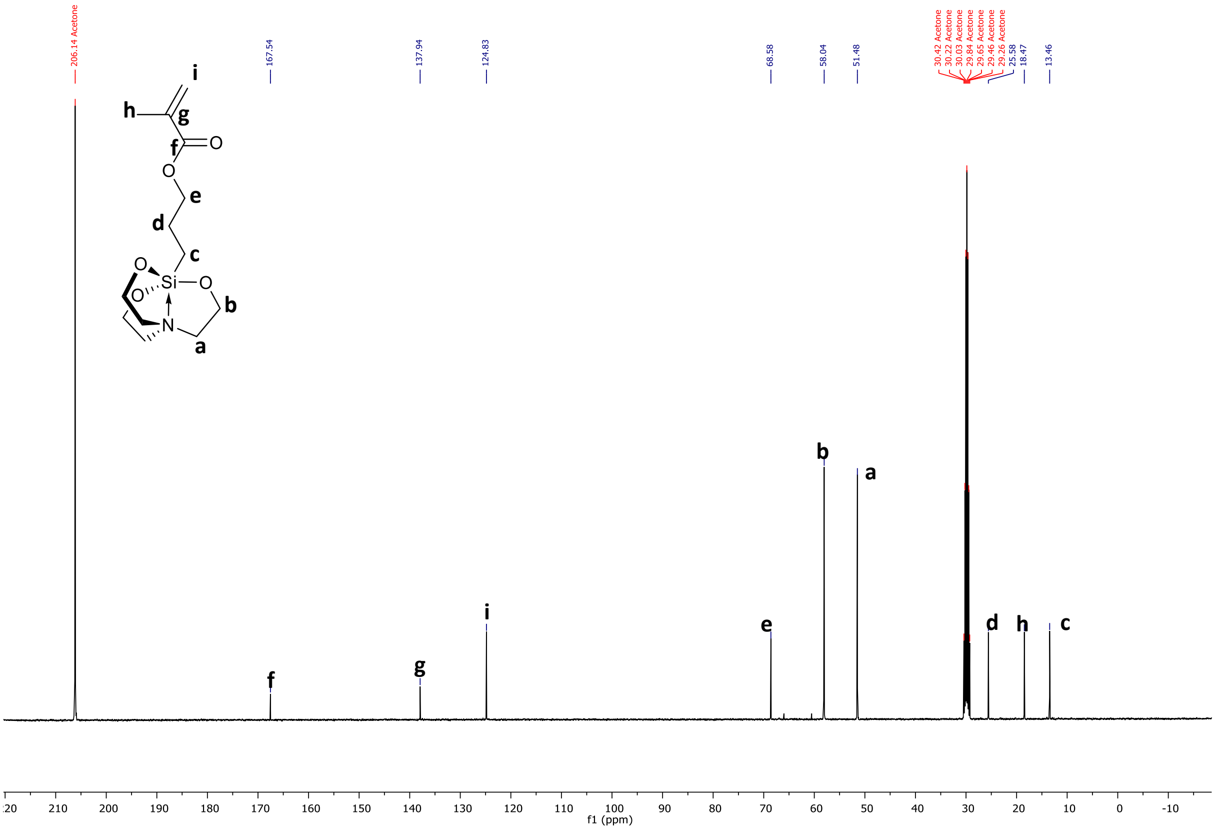

Figure S30. ^{29}Si NMR spectrum of **3i** (79 MHz, $(CD_3)_2CO$).

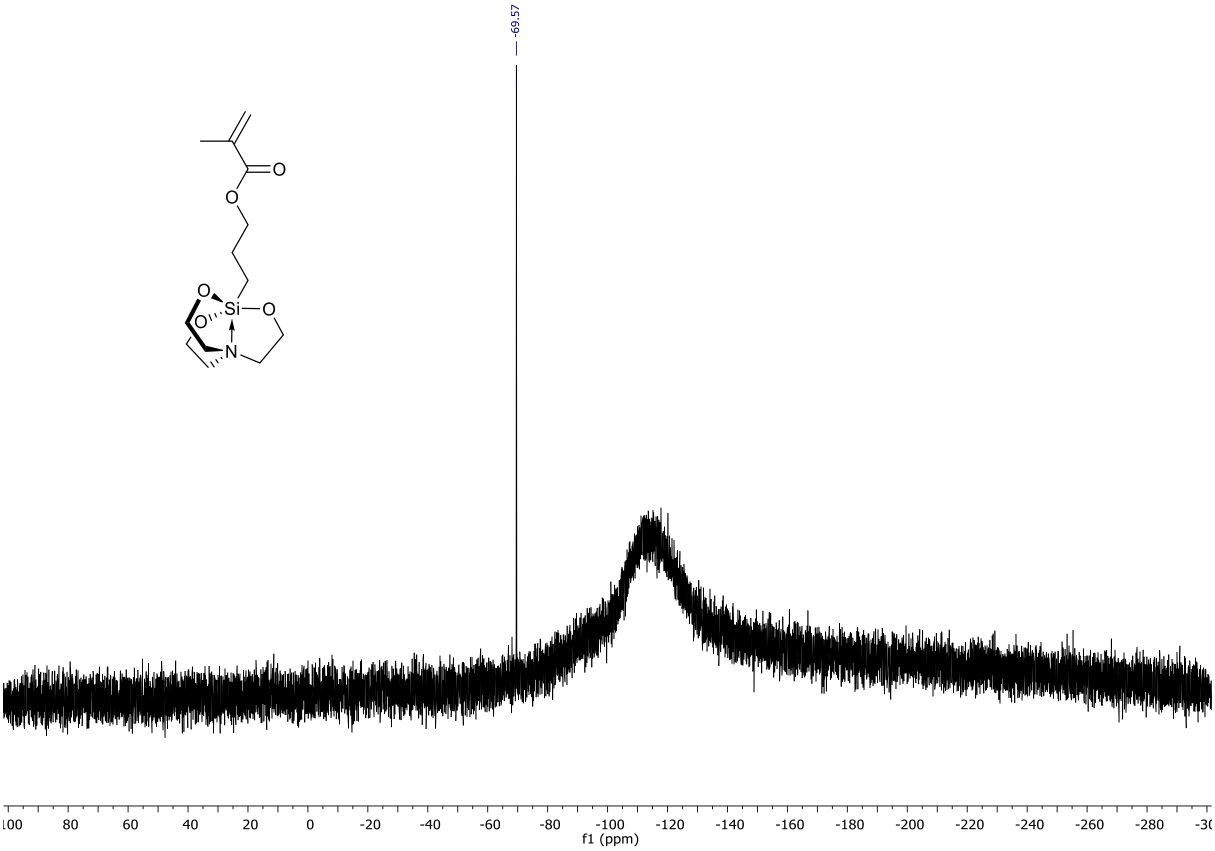

Figure S31. ^{1}H NMR spectrum of **3j** (400 MHz, $CDCl_3$).

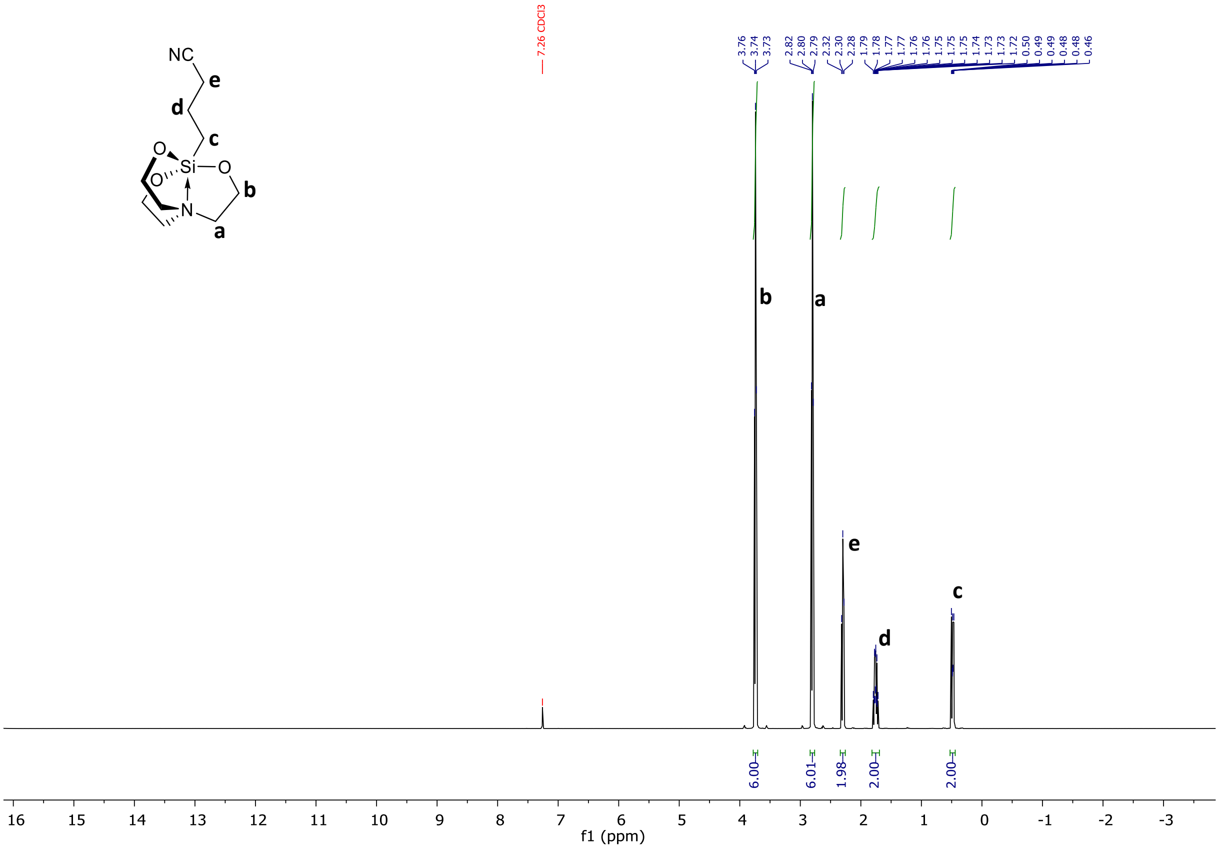

Figure S32. ^{13}C NMR spectrum of **3j** (101 MHz, $CDCl_3$).

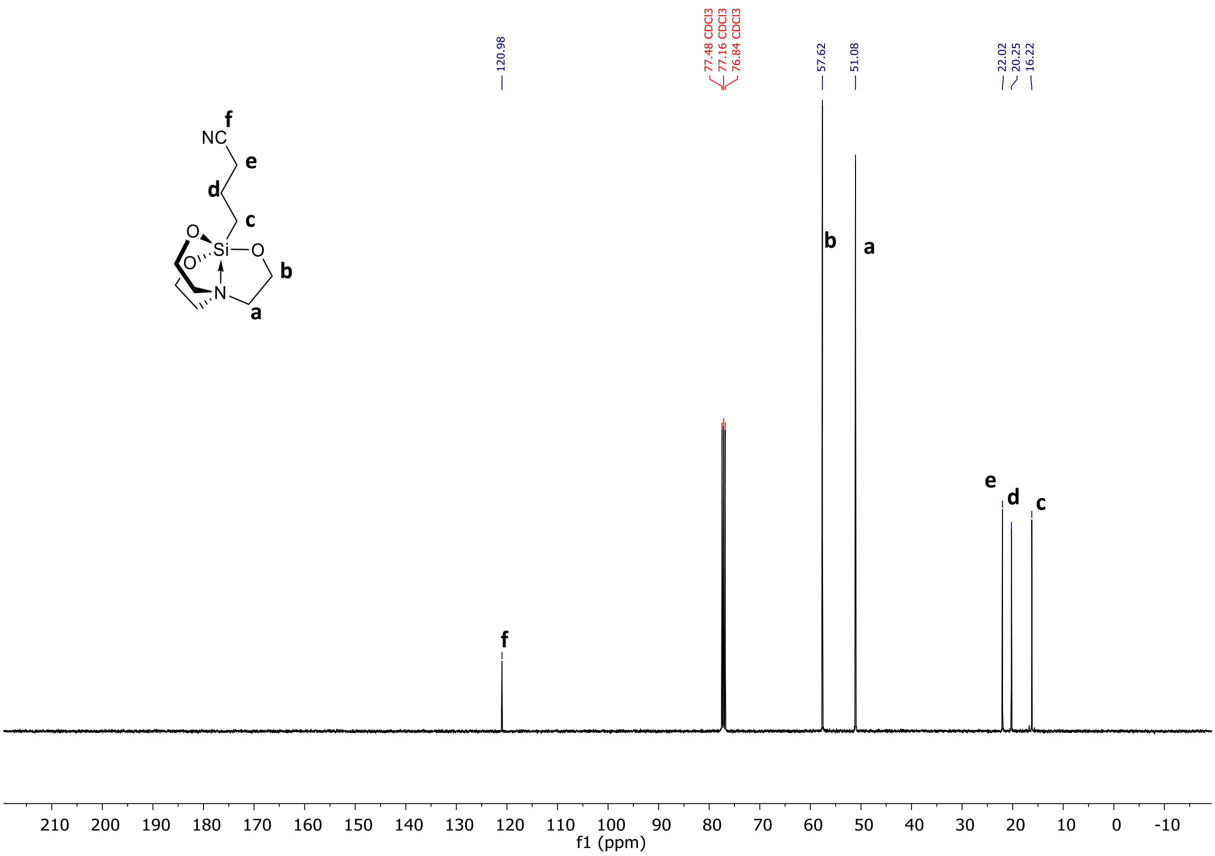

Figure S33. ^{29}Si NMR spectrum of **3j** (79 MHz, $CDCl_3$).

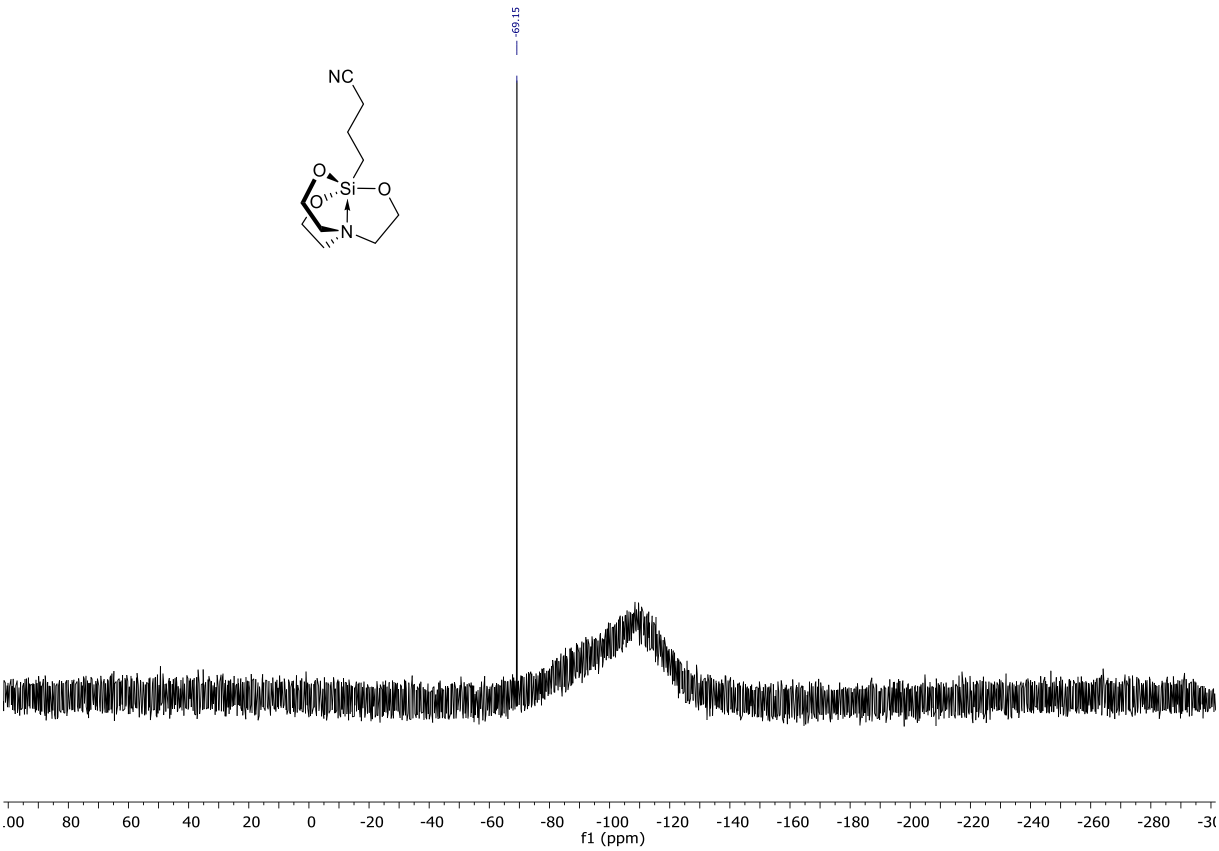

Figure S34. ^{1}H NMR spectrum of **3k** (400 MHz, $(CD_3)_2CO$).

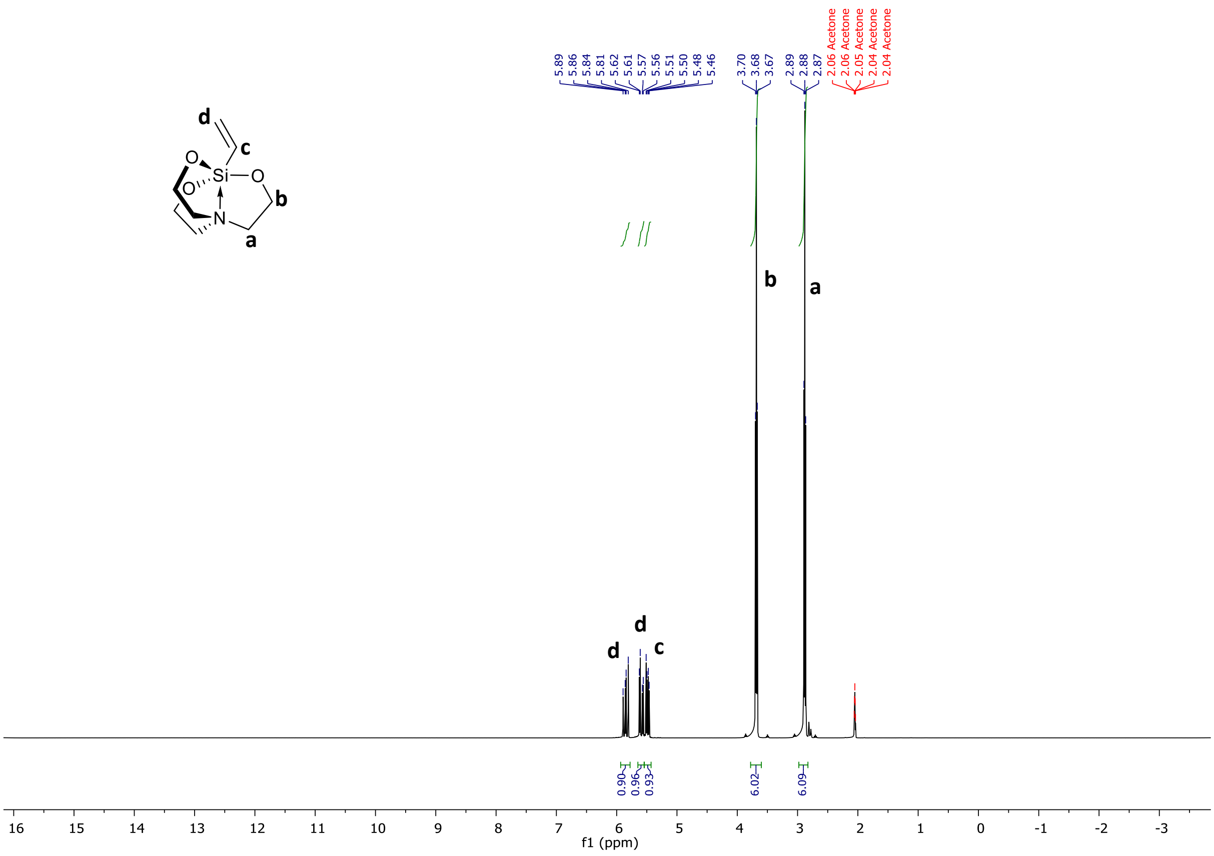

Figure S35. ^{13}C NMR spectrum of **3k** (101 MHz, $(CD_3)_2CO$).

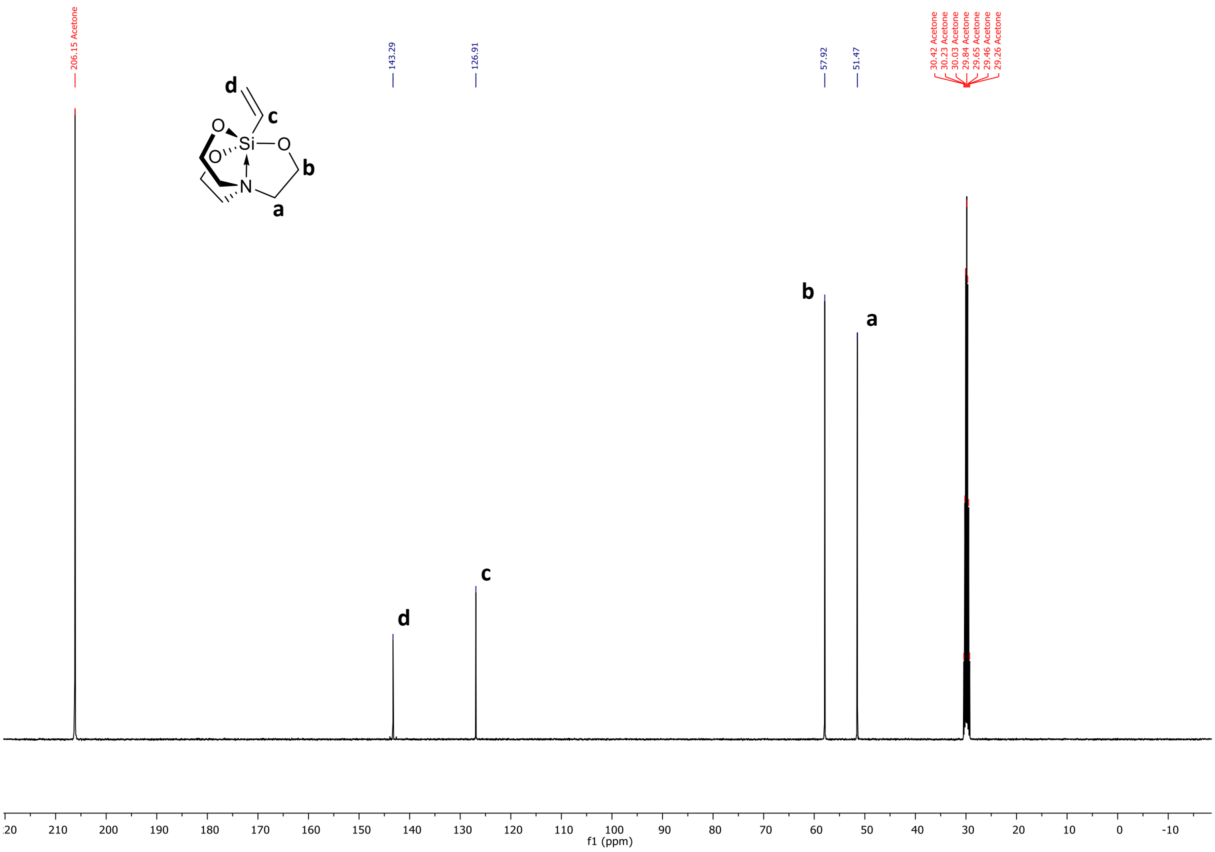

Figure S36. ^{29}Si NMR spectrum of **3k** (79 MHz, $(CD_3)_2CO$).

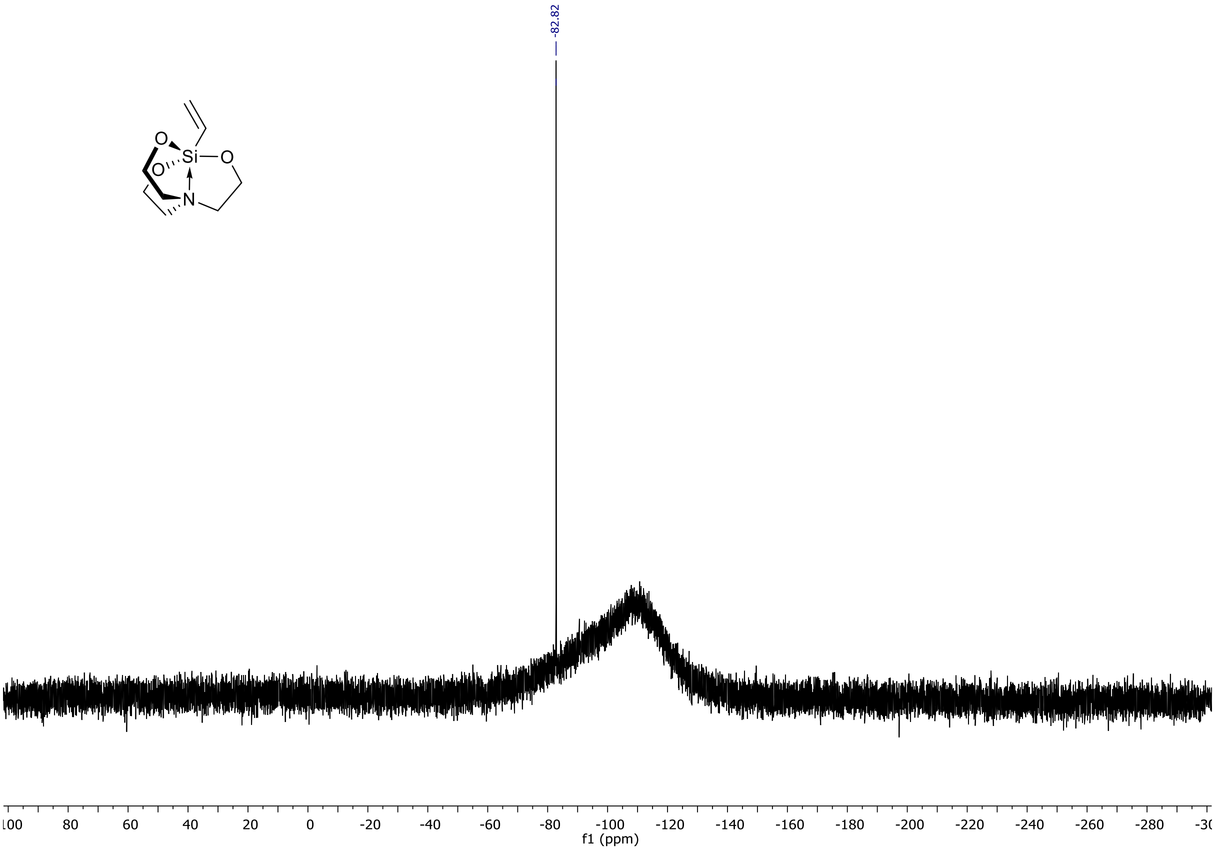

Figure S37. ^{1}H NMR spectrum of **3l** (400 MHz, CD_3OD)

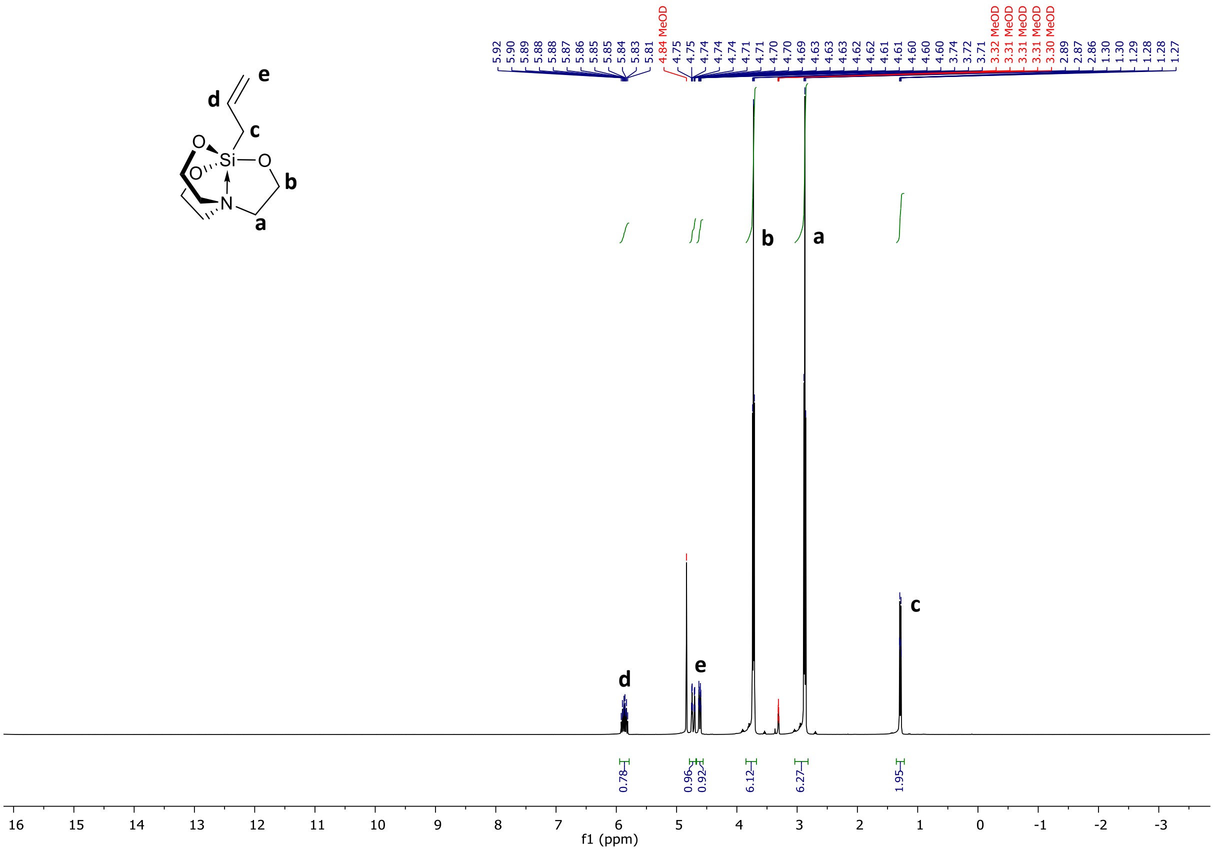

Figure S38. ^{13}C NMR spectrum of **3l** (101 MHz, CD_3OD).

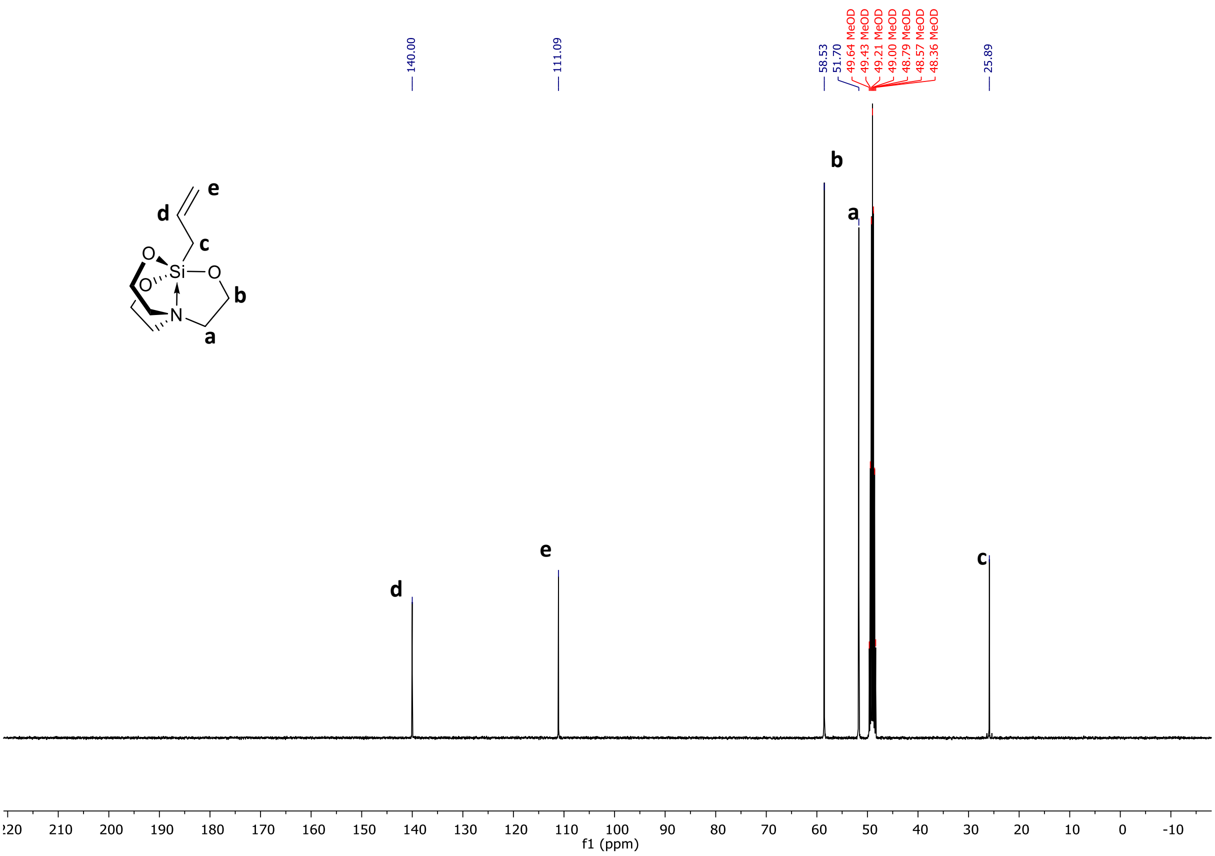

Figure S39. ^{29}Si NMR spectrum of **3l** (79 MHz, CD_3OD).

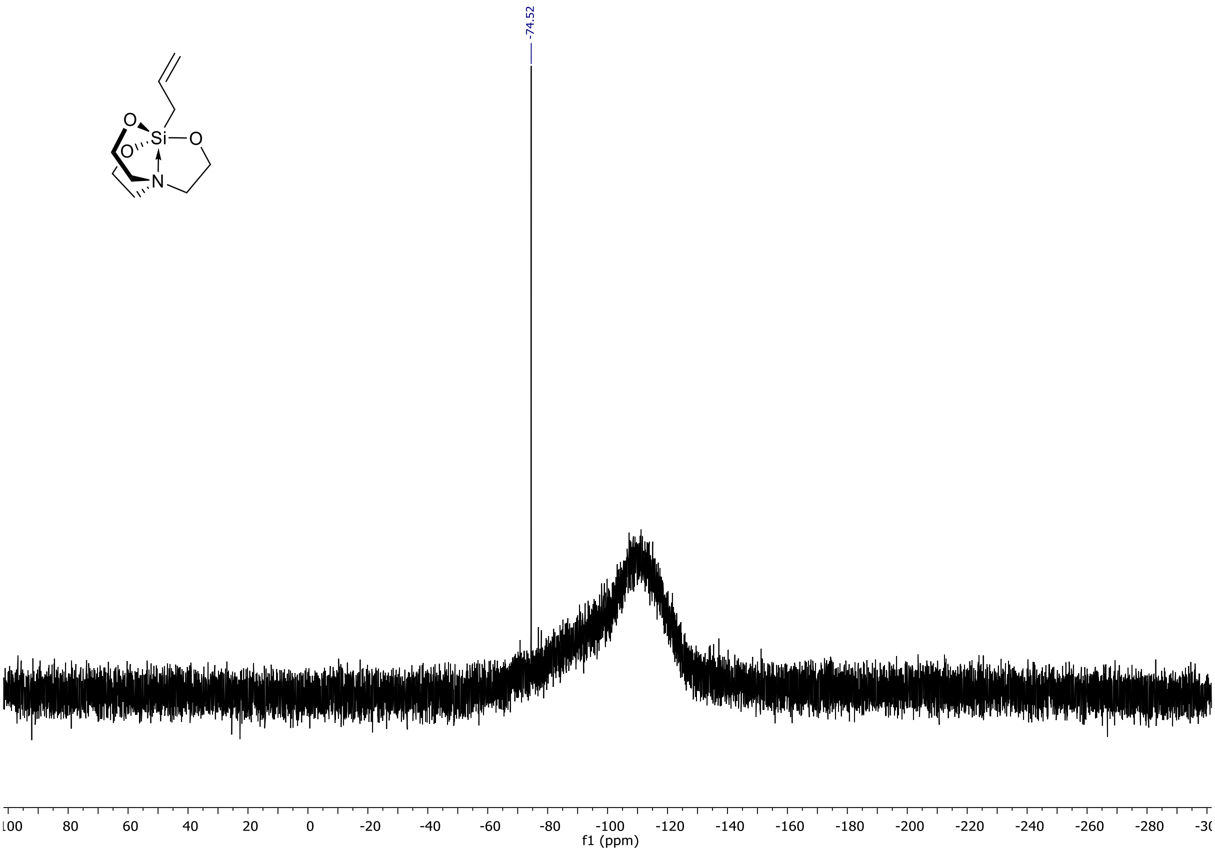

Figure S40. ^{1}H NMR spectrum of **3m** (400 MHz, $CDCl_3$).

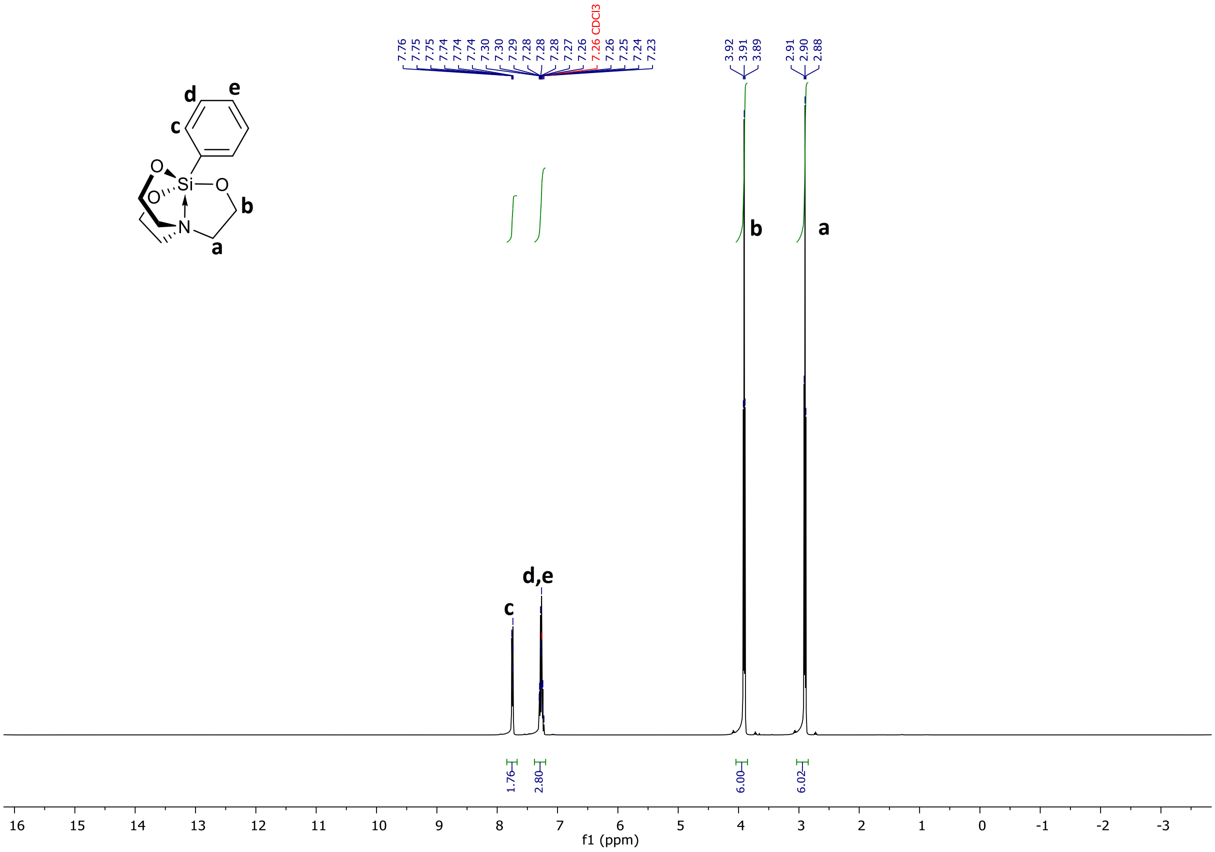

Figure S41. ^{13}C NMR spectrum of **3m** (101 MHz, $CDCl_3$).

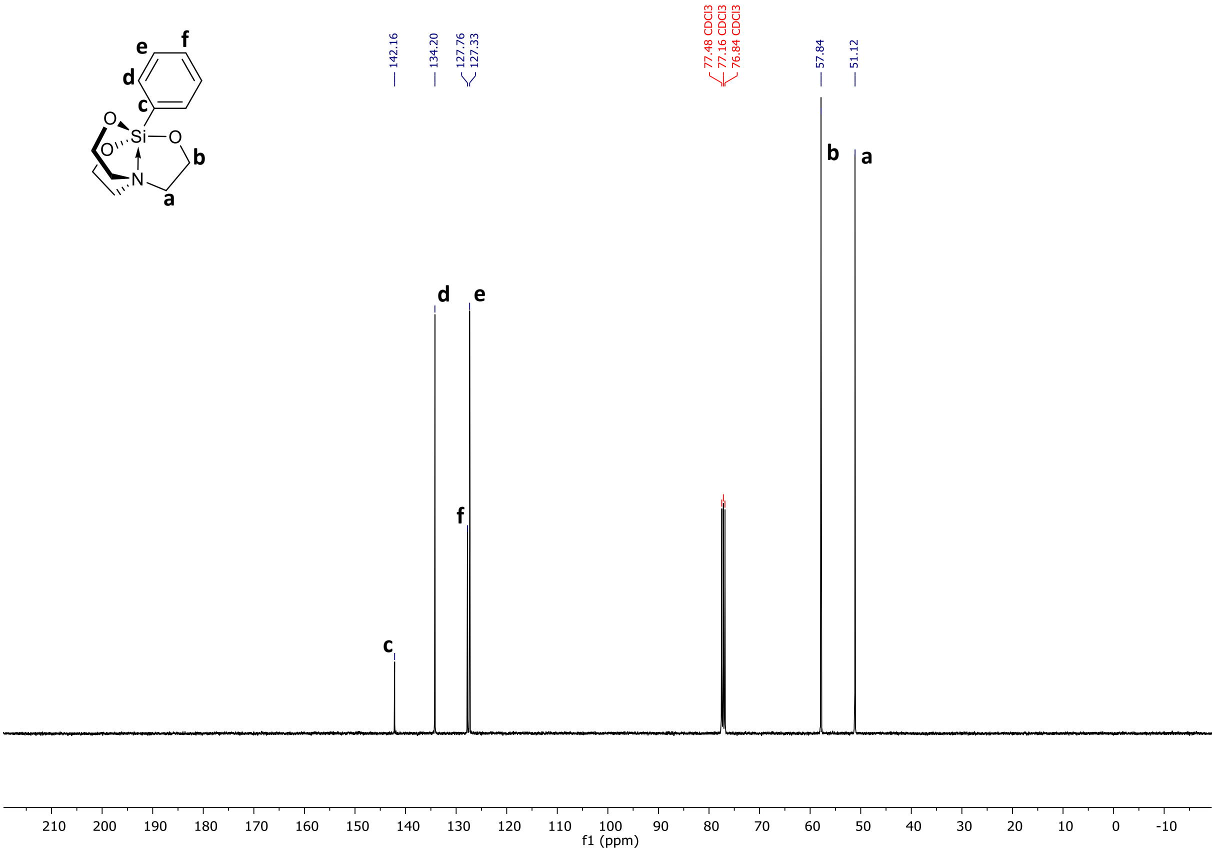

Figure S42. ^{29}Si NMR spectrum of **3m** (79 MHz, $CDCl_3$).

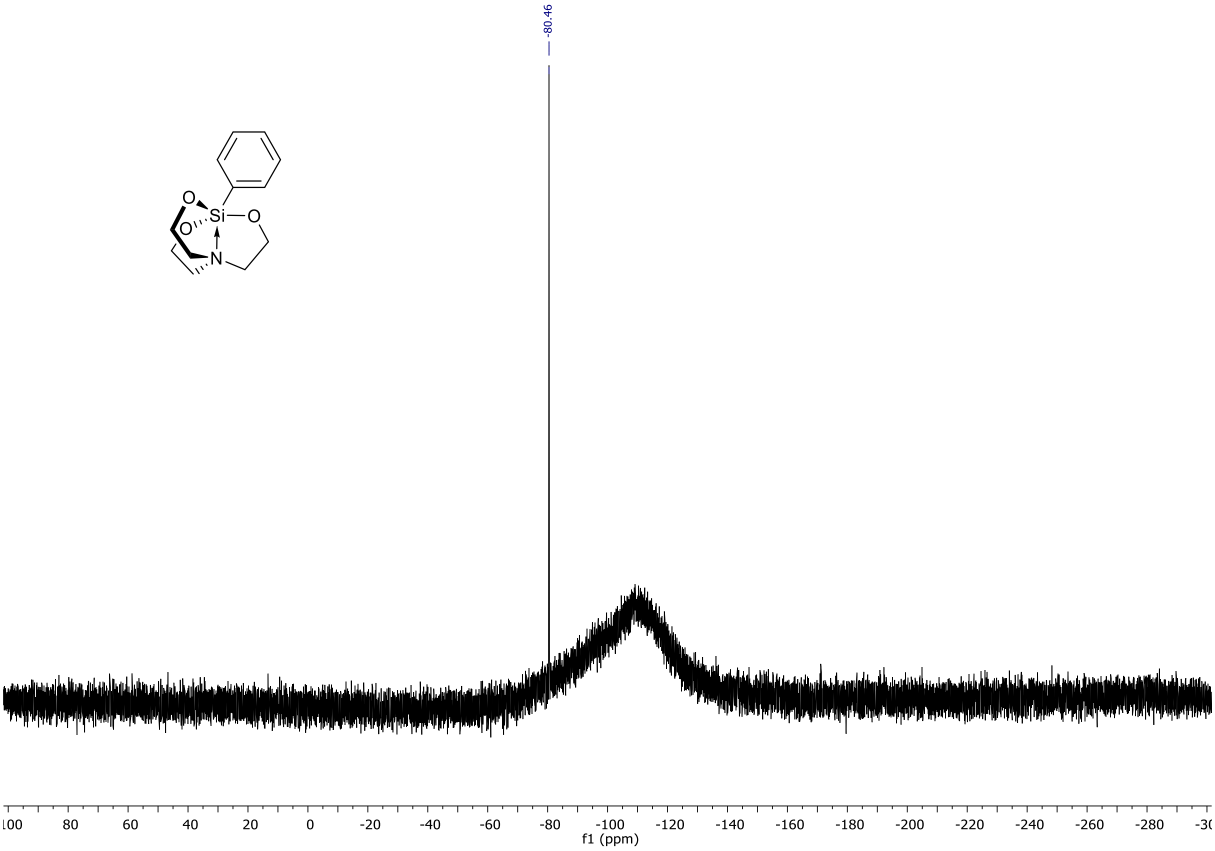

Figure S43. ^{1}H NMR spectrum of **3n** (400 MHz, $CDCl_3$).

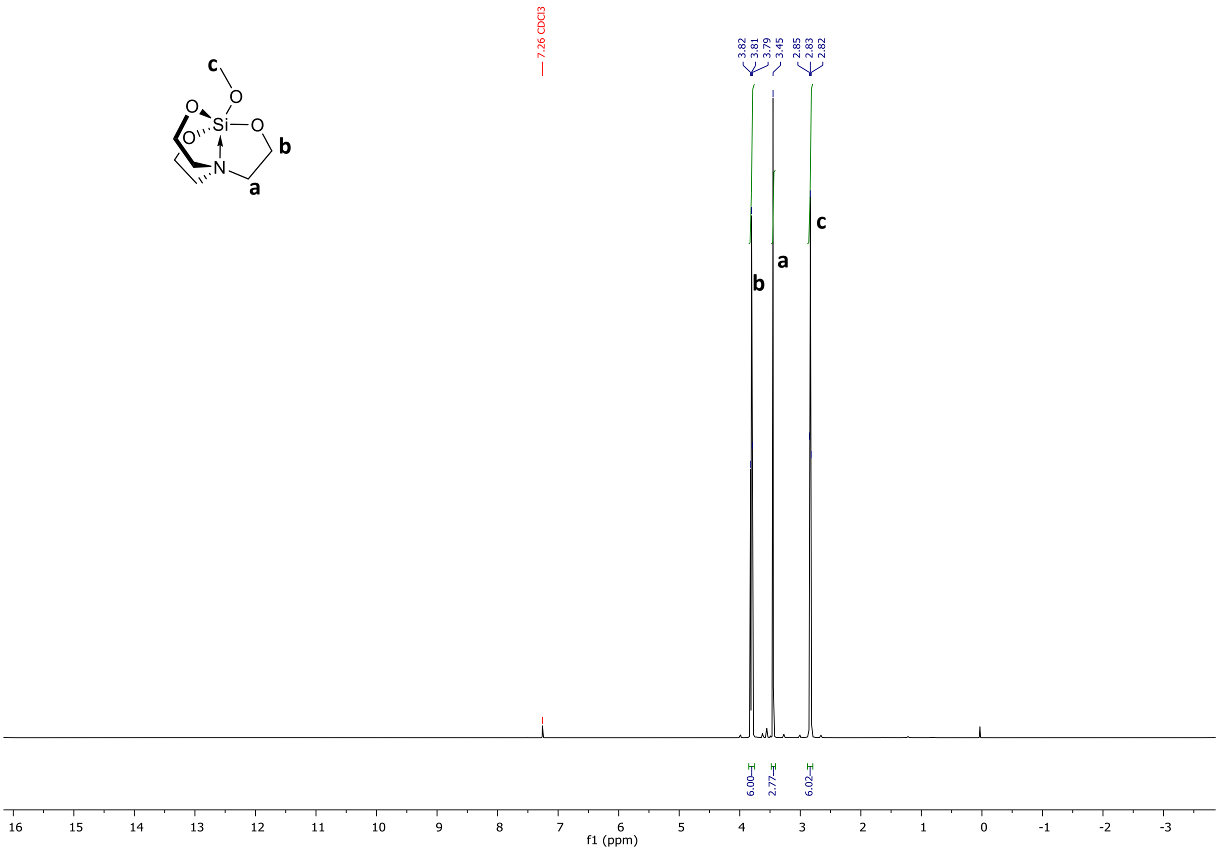

Figure S44. ^{13}C NMR spectrum of **3n** (101 MHz, $CDCl_3$).

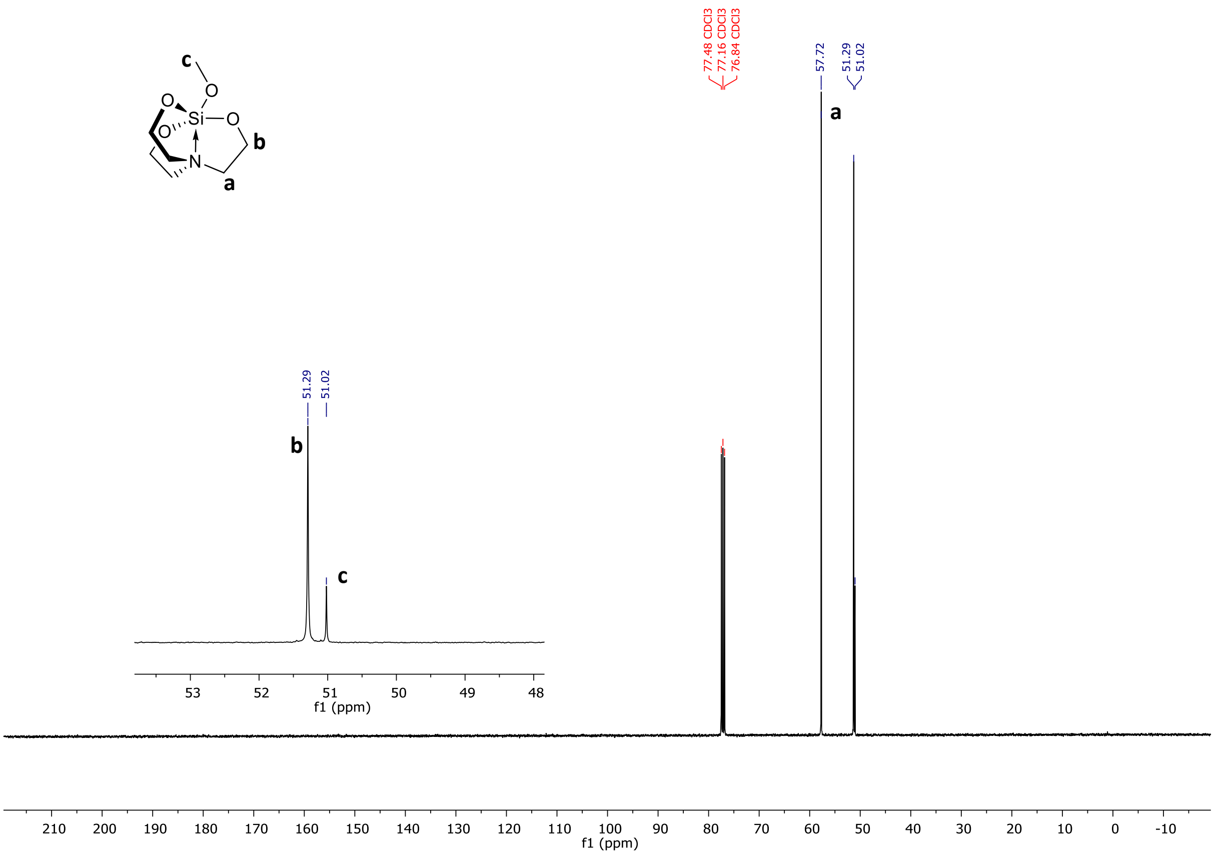

Figure S45. ^{29}Si NMR spectrum of **3n** (79 MHz, $CDCl_3$).

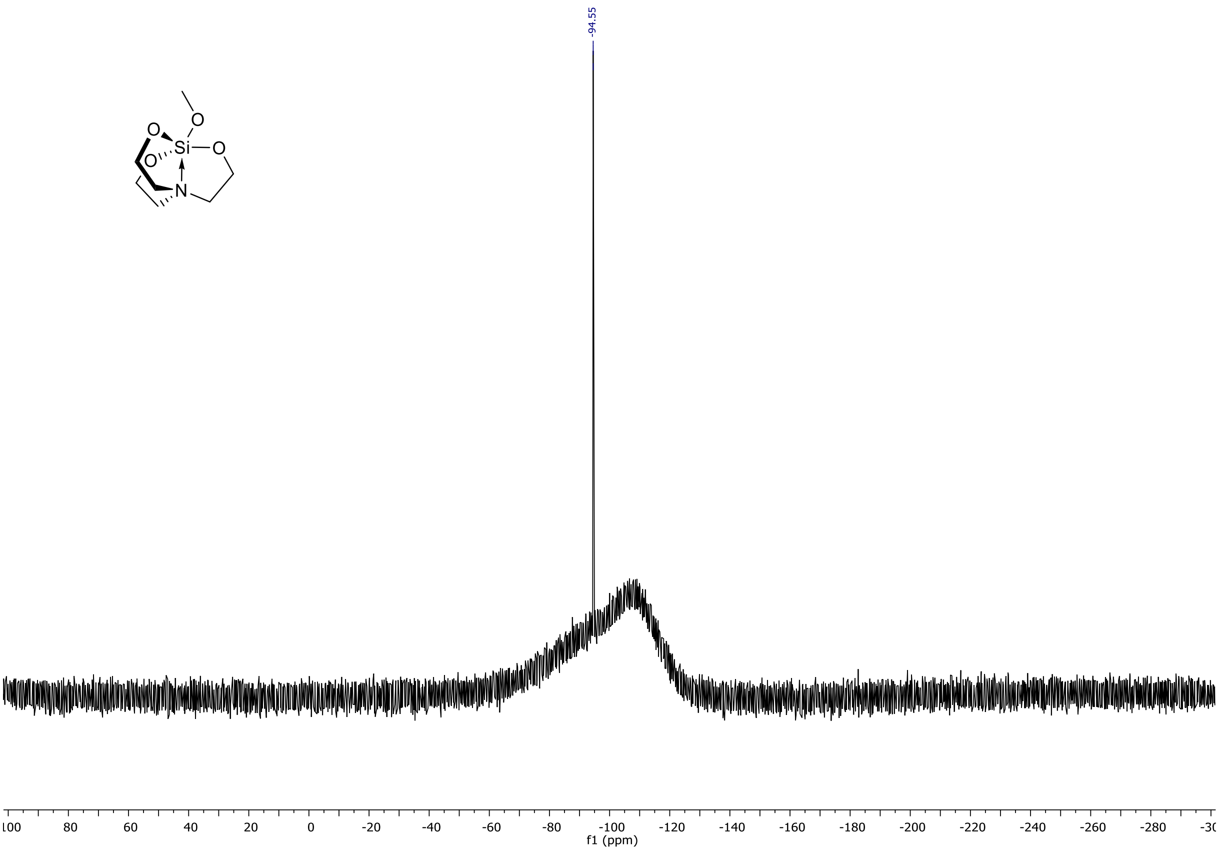

Figure S46. ^{1}H NMR spectrum of **3o** (400 MHz, $CDCl_3$).

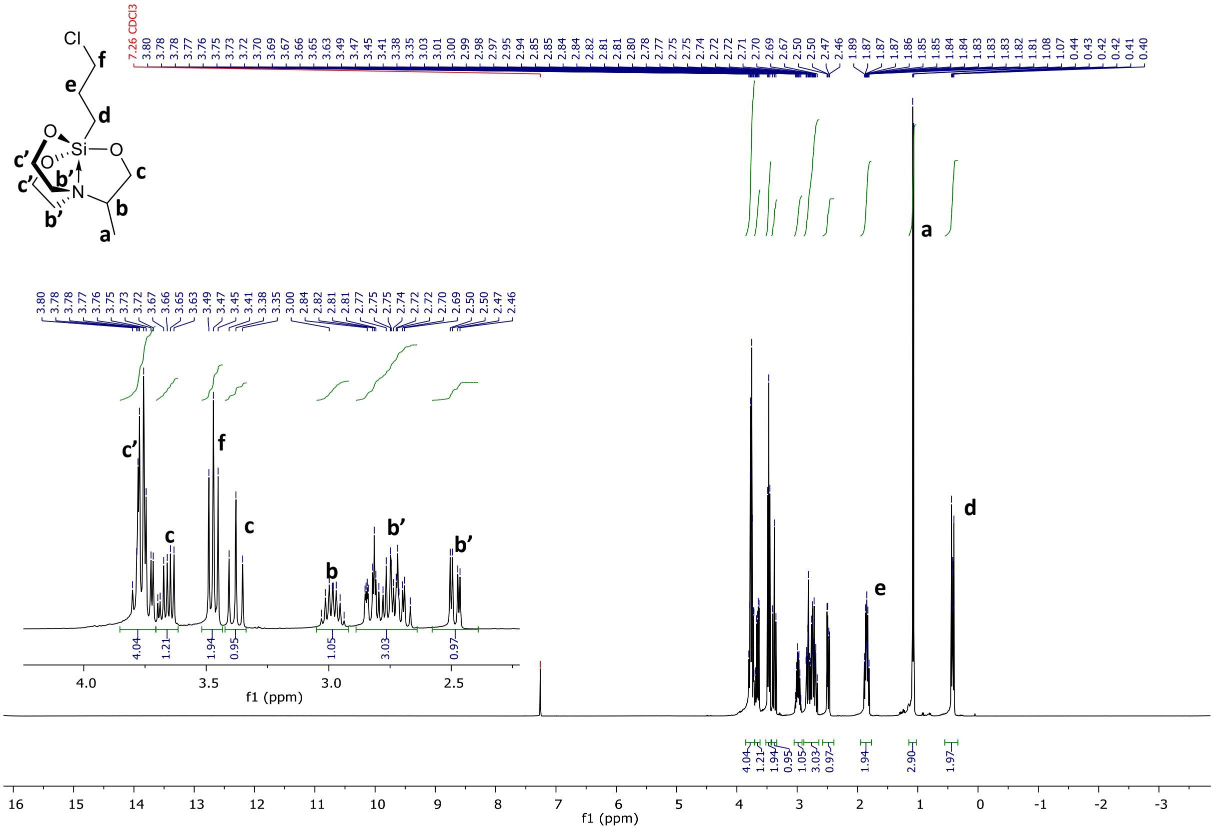

Figure S47. ^{13}C NMR spectrum of **3o** (101 MHz, $CDCl_3$).

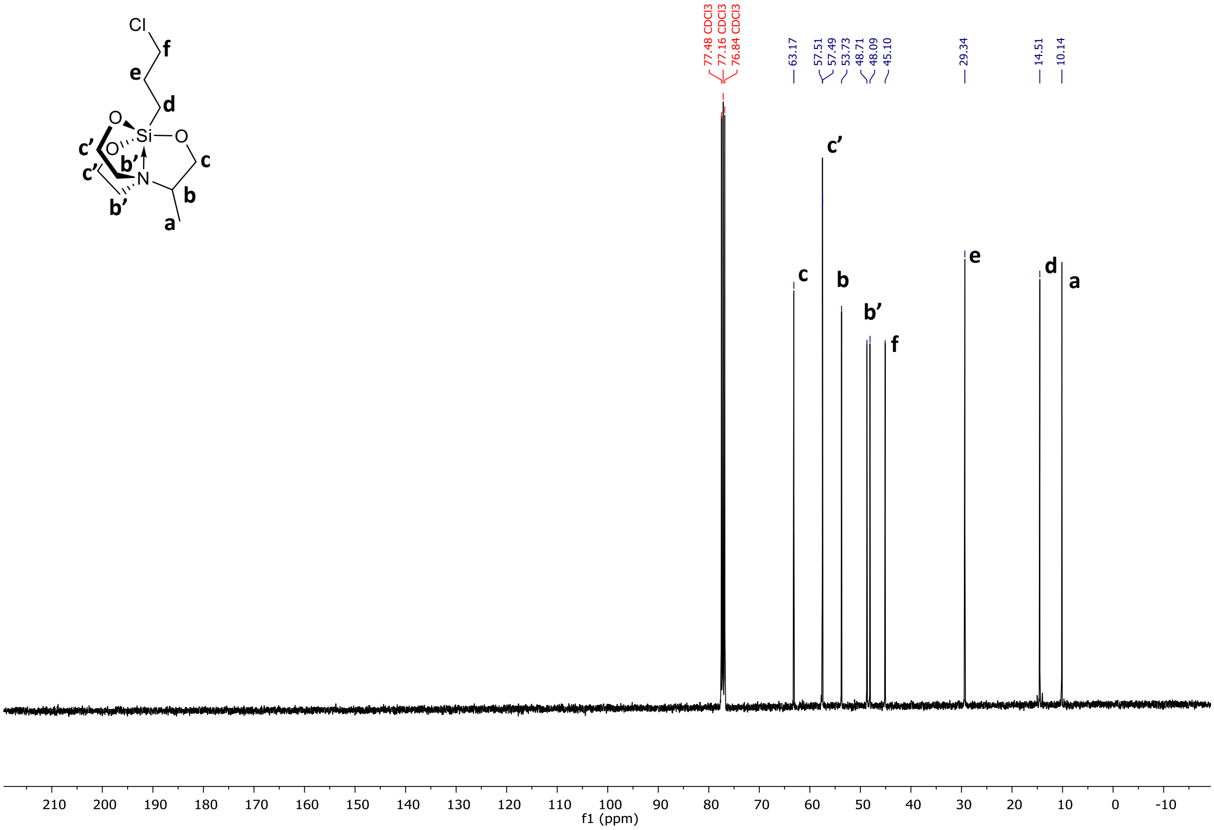

Figure S48. ^{29}Si NMR spectrum of **3o** (79 MHz, $CDCl_3$).

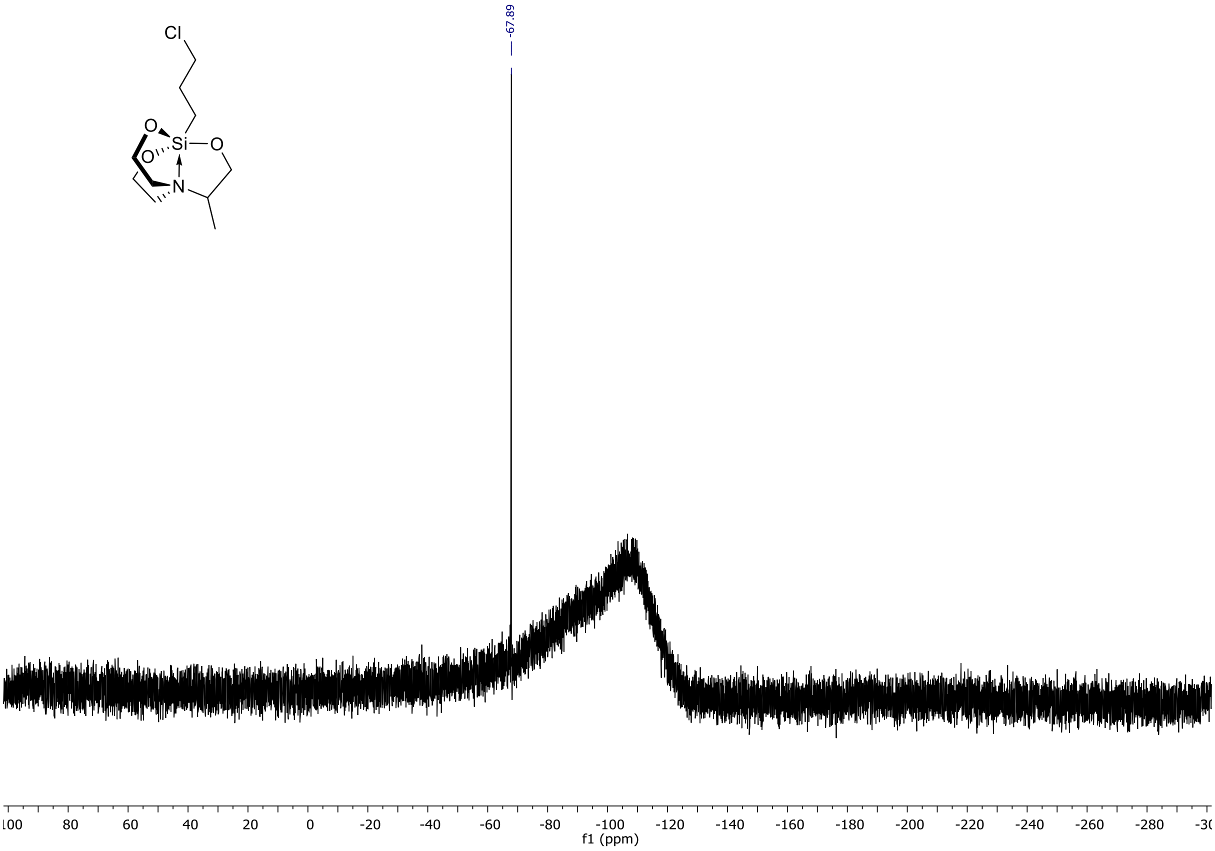

Figure S49. ^{1}H NMR spectrum of **3p** (400 MHz, $CDCl_3$).

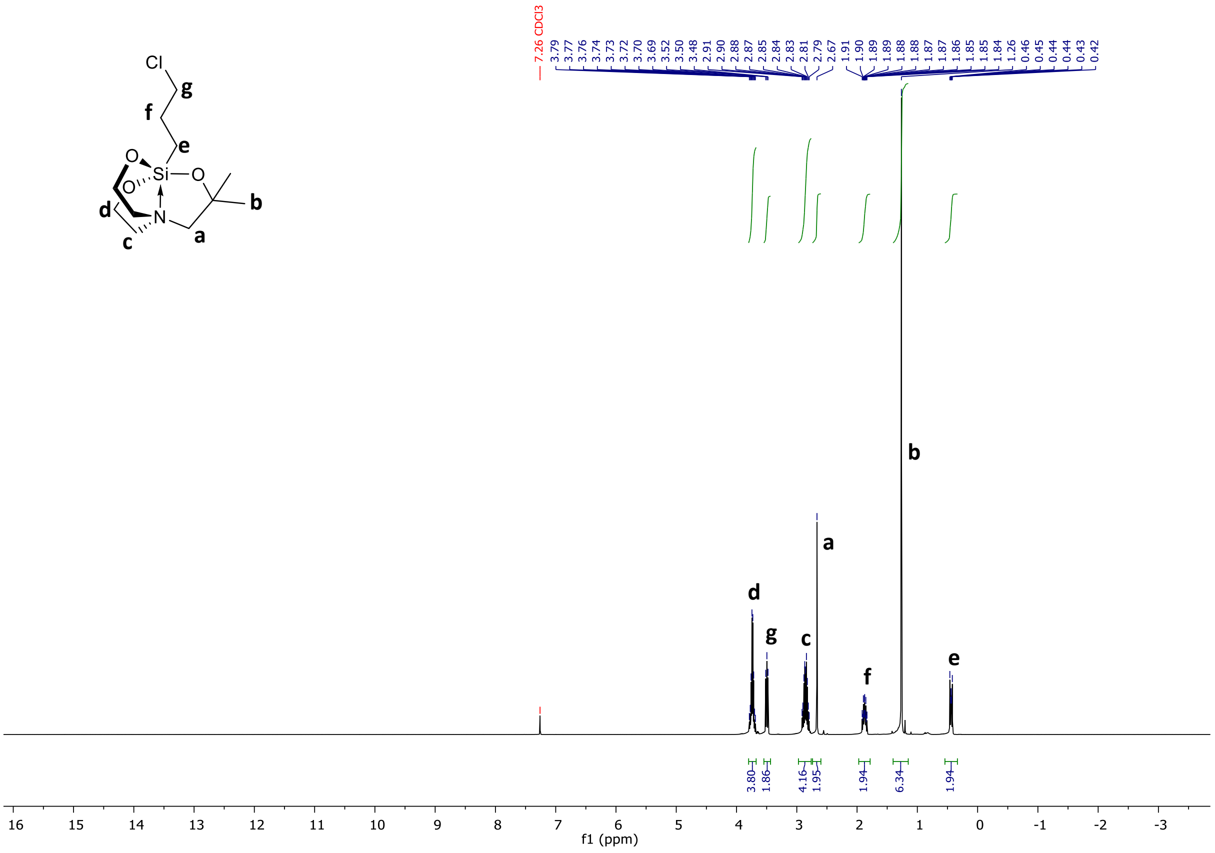

Figure S50. ^{13}C NMR spectrum of **3p** (101 MHz, $CDCl_3$).

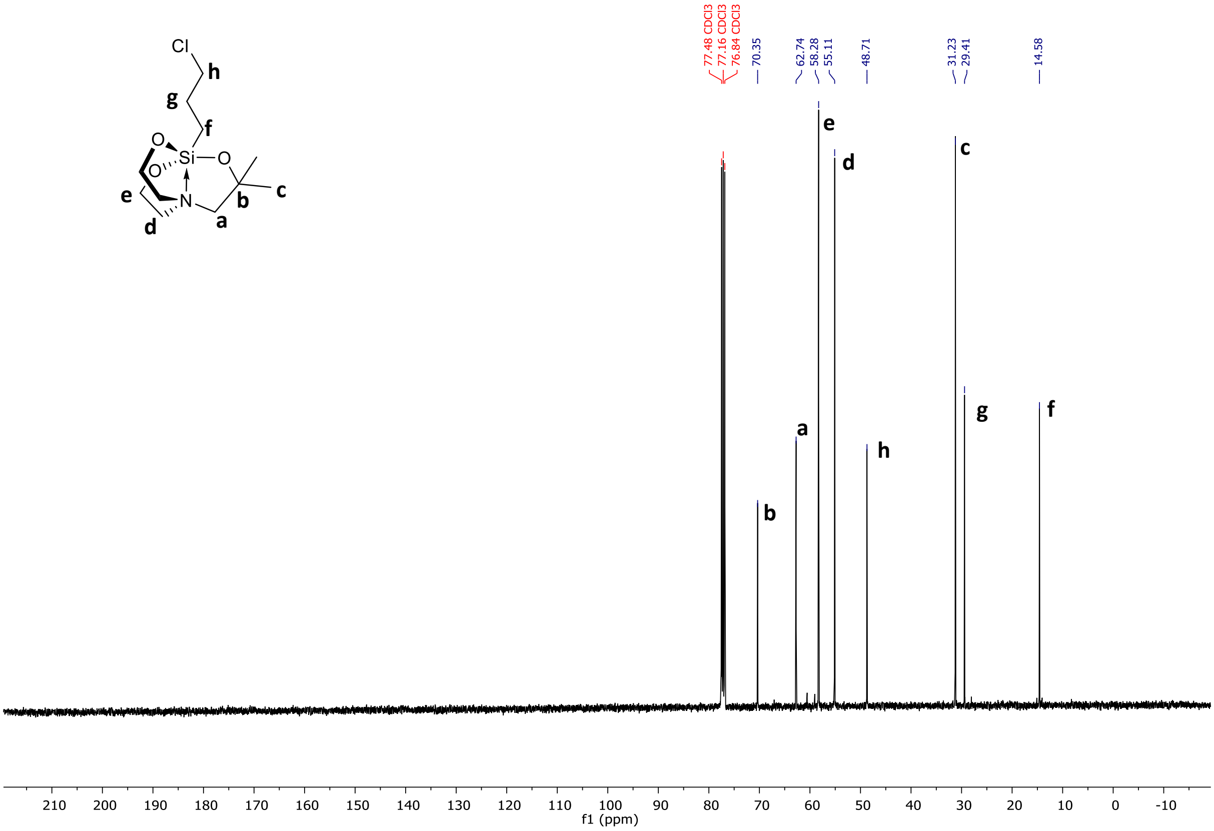

Figure S51. ^{29}Si NMR spectrum of **3p** (79 MHz, $CDCl_3$).

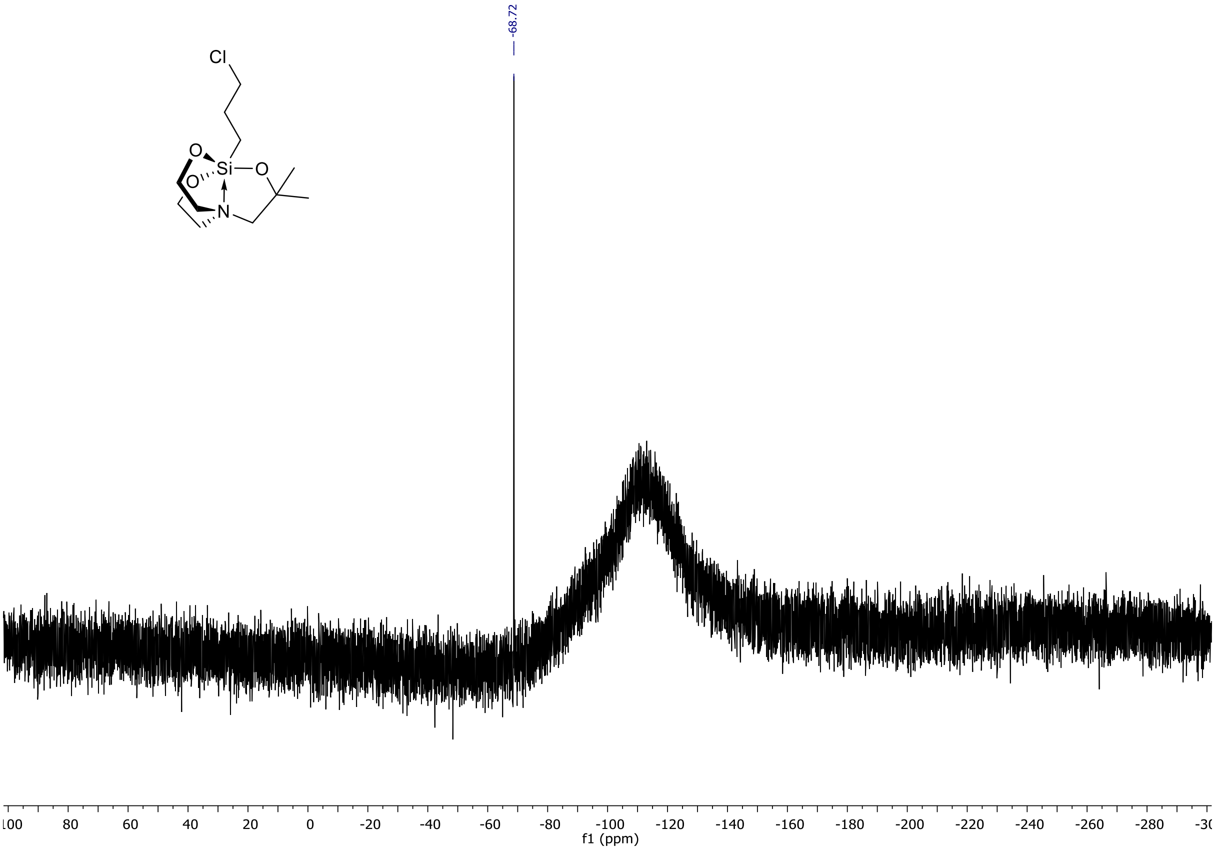

Figure S52. ^{1}H NMR spectrum of **3q** (400 MHz, $CDCl_3$).

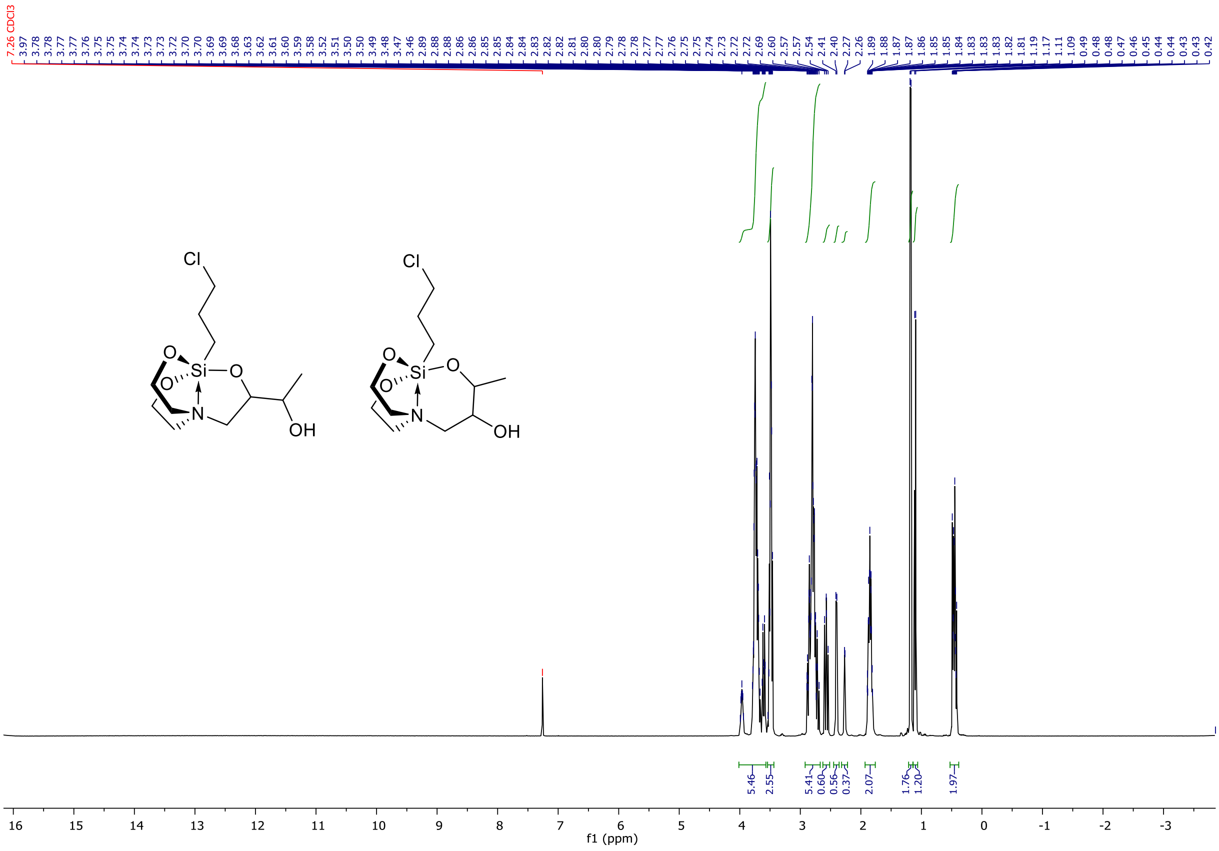

Figure S53. ^{13}C NMR spectrum of **3q** (101 MHz, $CDCl_3$).

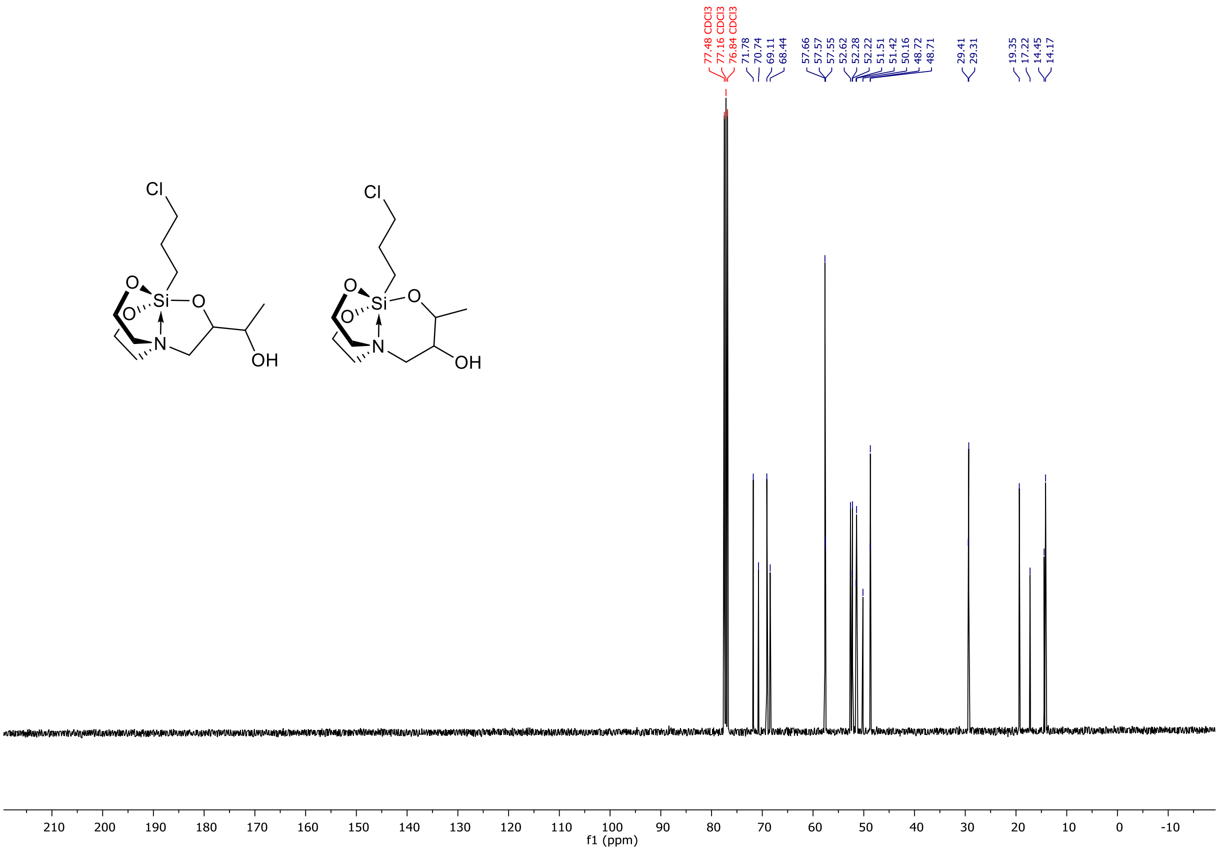

Figure S54. ^{29}Si NMR spectrum of **3q** (79 MHz, $CDCl_3$).

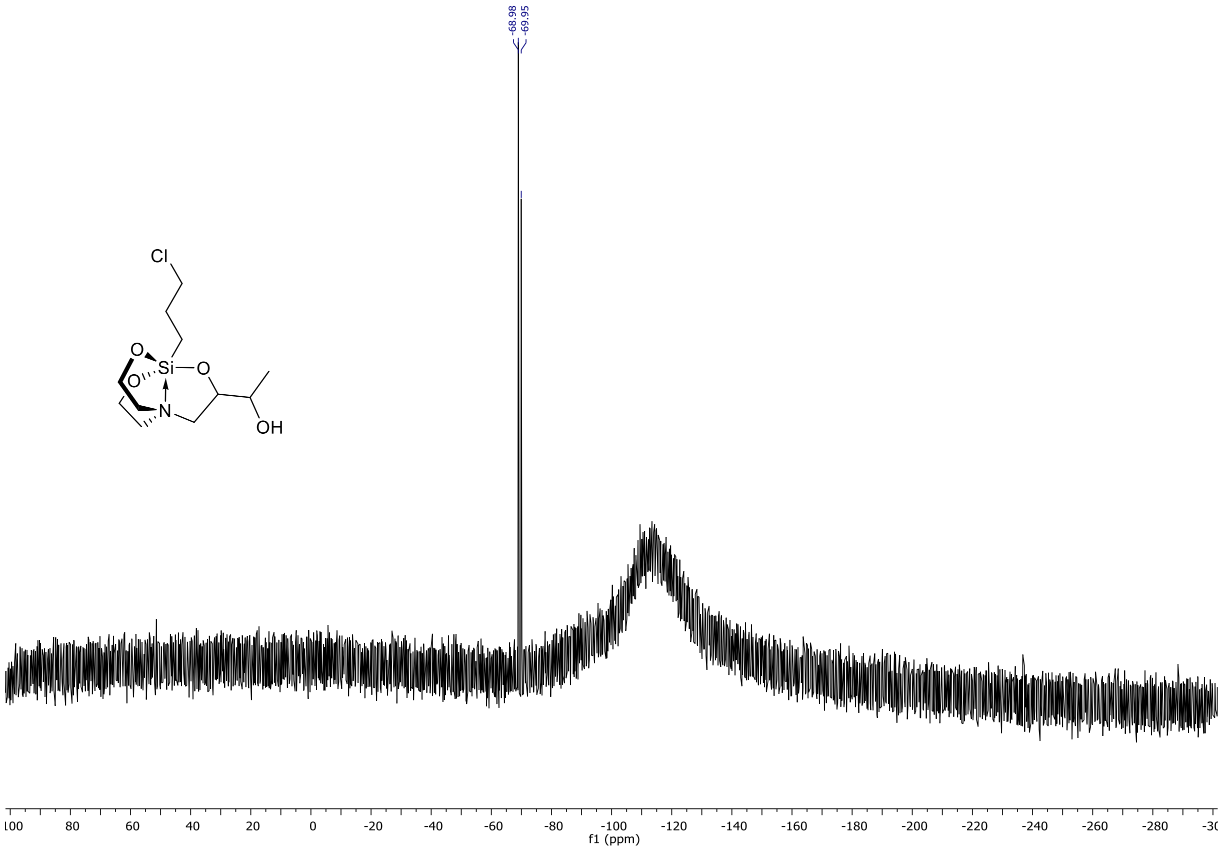

Figure S55. ^{1}H NMR spectrum of **3r** (400 MHz, $CDCl_3$).

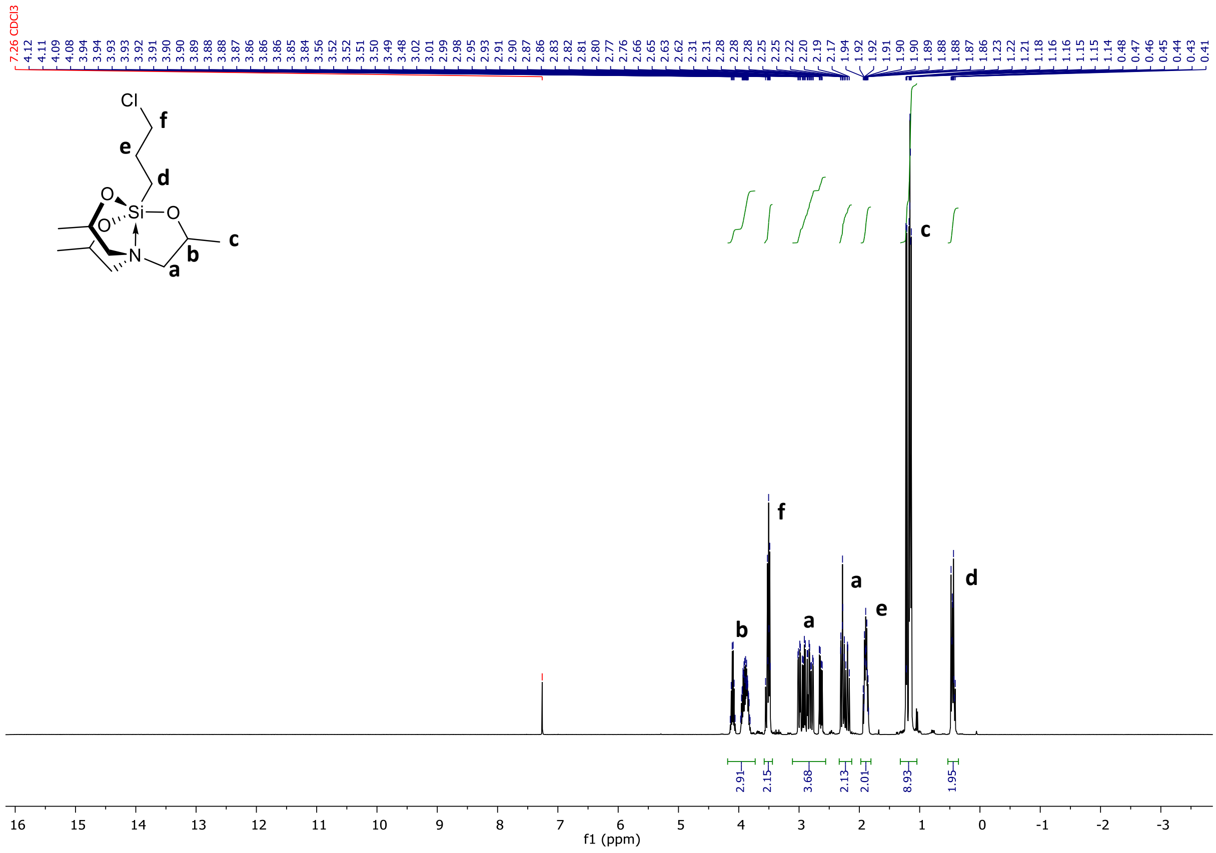

Figure S56. ^{13}C NMR spectrum of **3r** (101 MHz, $CDCl_3$).

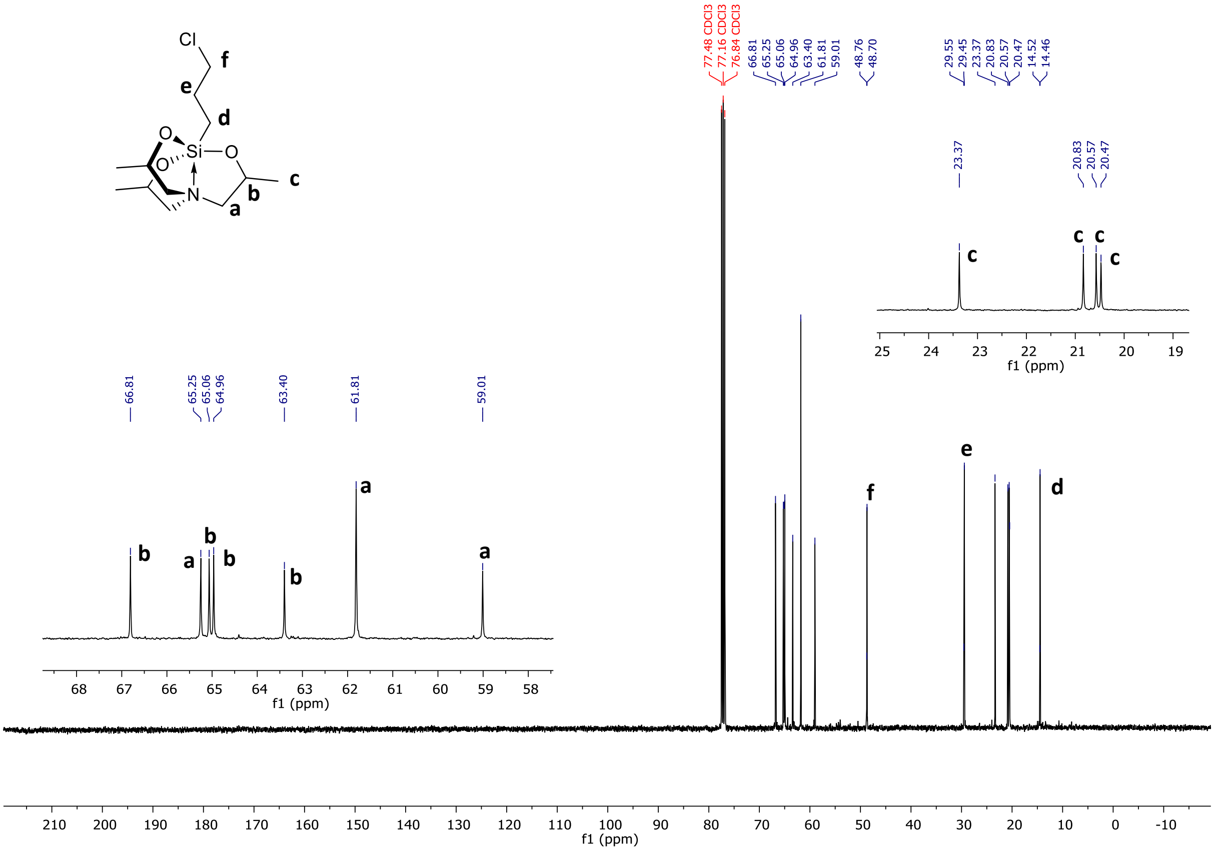

Figure S57. ^{29}Si NMR spectrum of **3r** (79 MHz, $CDCl_3$).

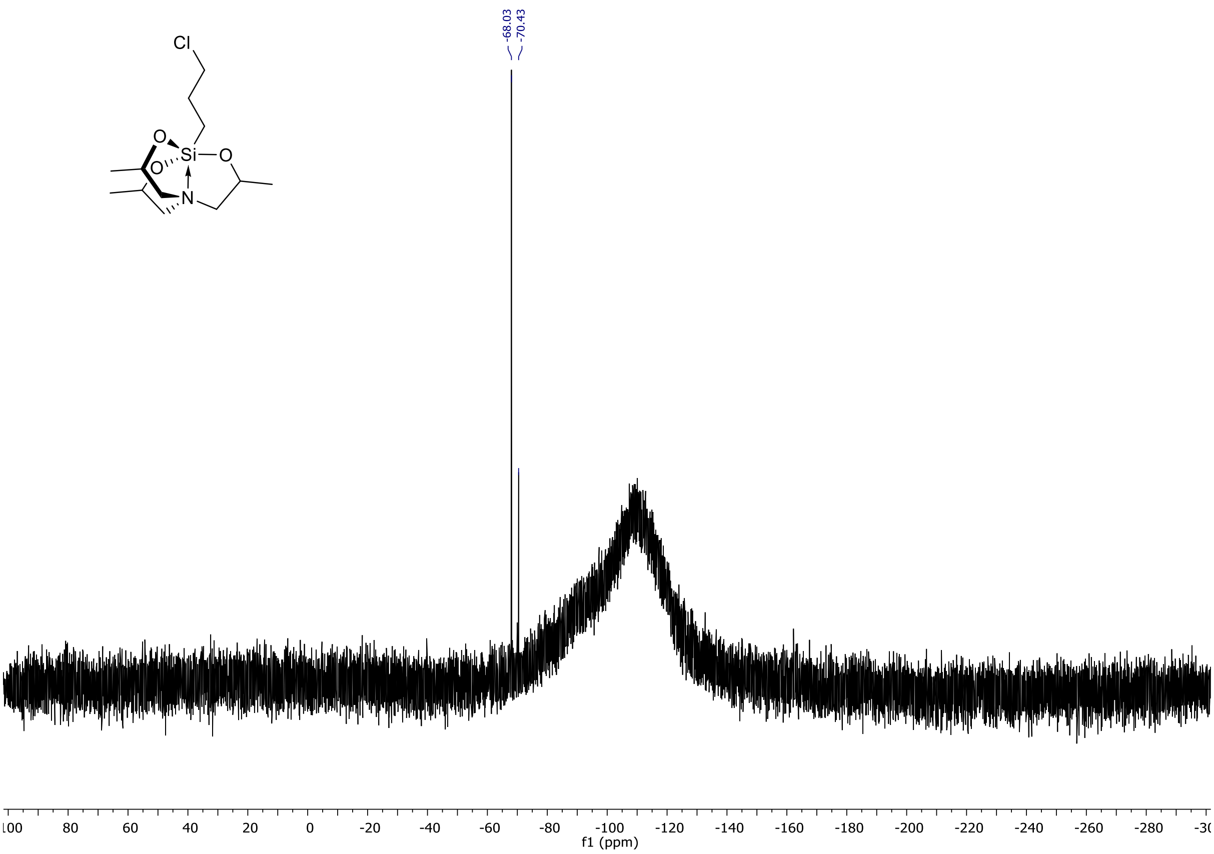

Figure S58. ^{1}H NMR spectrum of **3s** (400 MHz, CD_3CN).

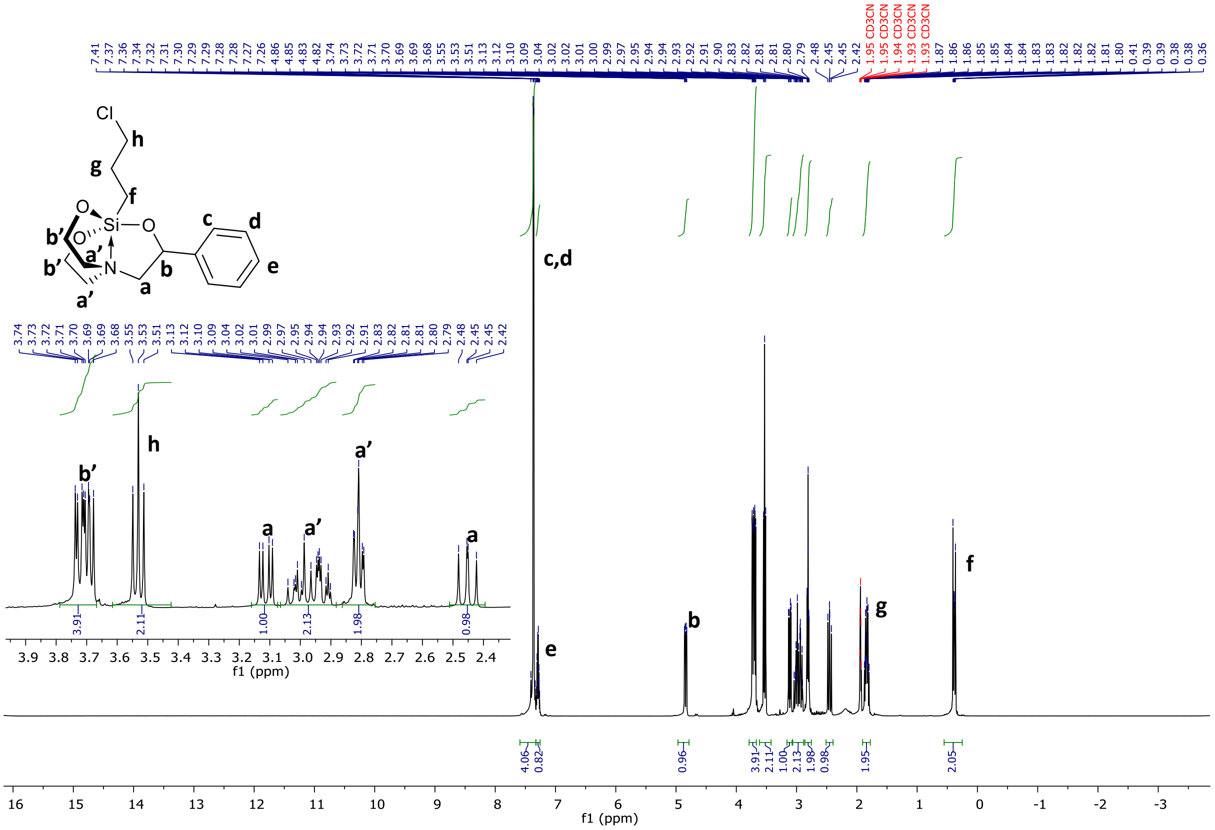

Figure S59. ^{13}C NMR spectrum of **3s** (101 MHz, CD_3CN).

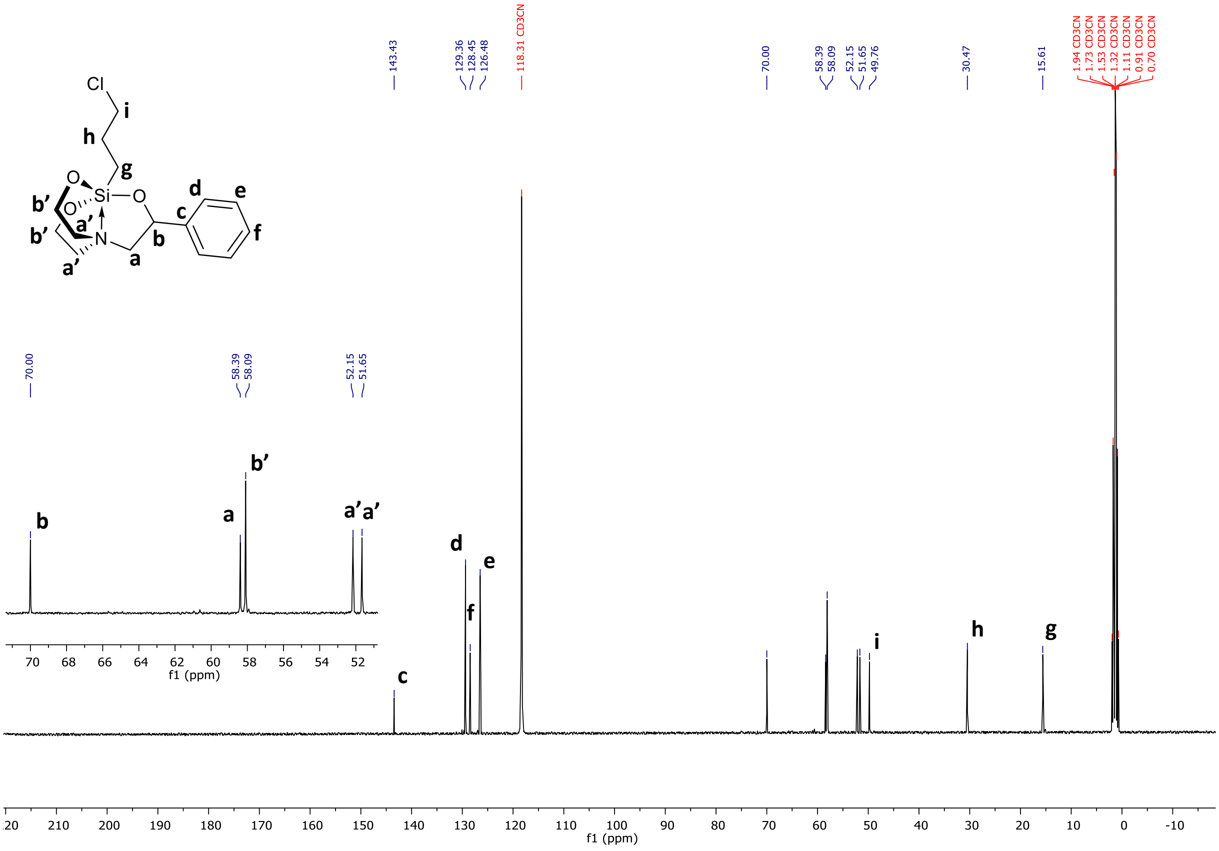

Figure S60. ^{29}Si NMR spectrum of **3s** (79 MHz, CD_3CN).

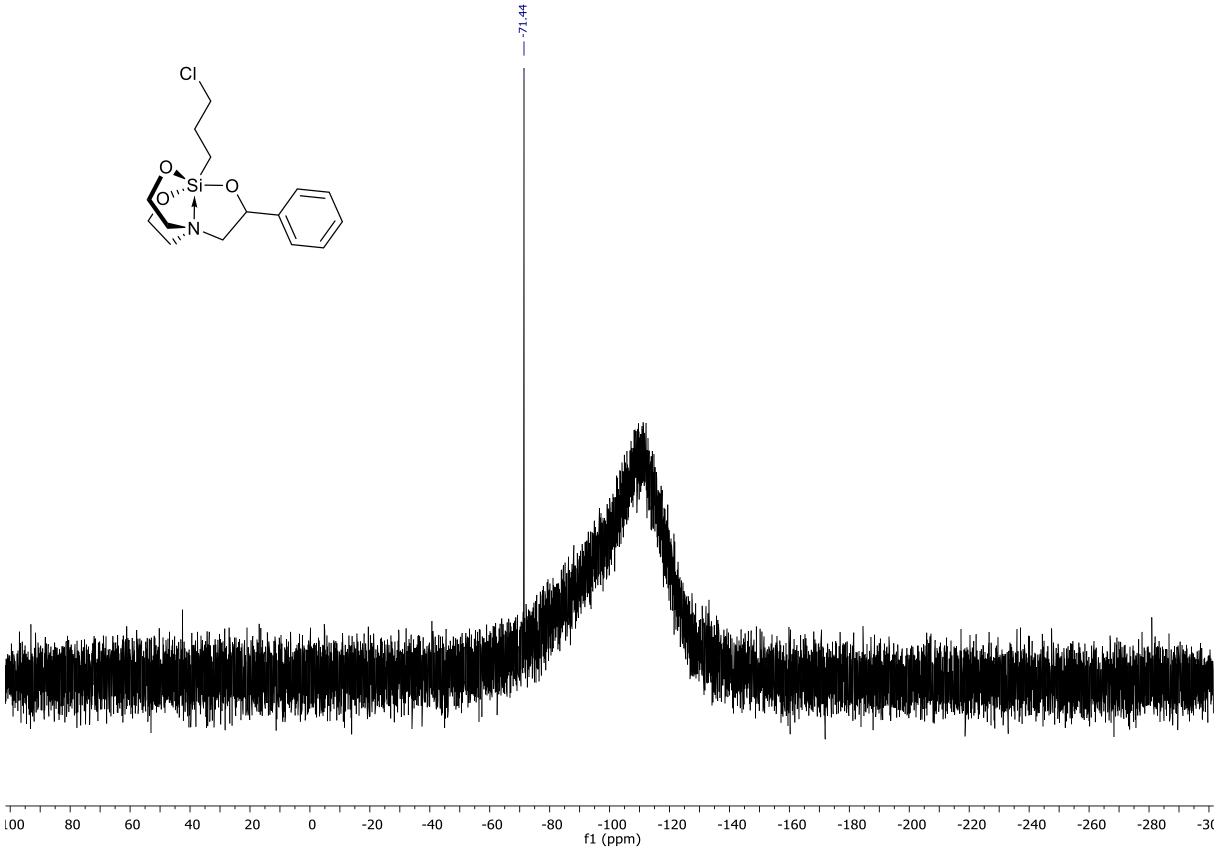

8. HRMS-ESI Spectra

Figure S61. HRMS spectrum of **3a**, predicted (left) and measured (right).

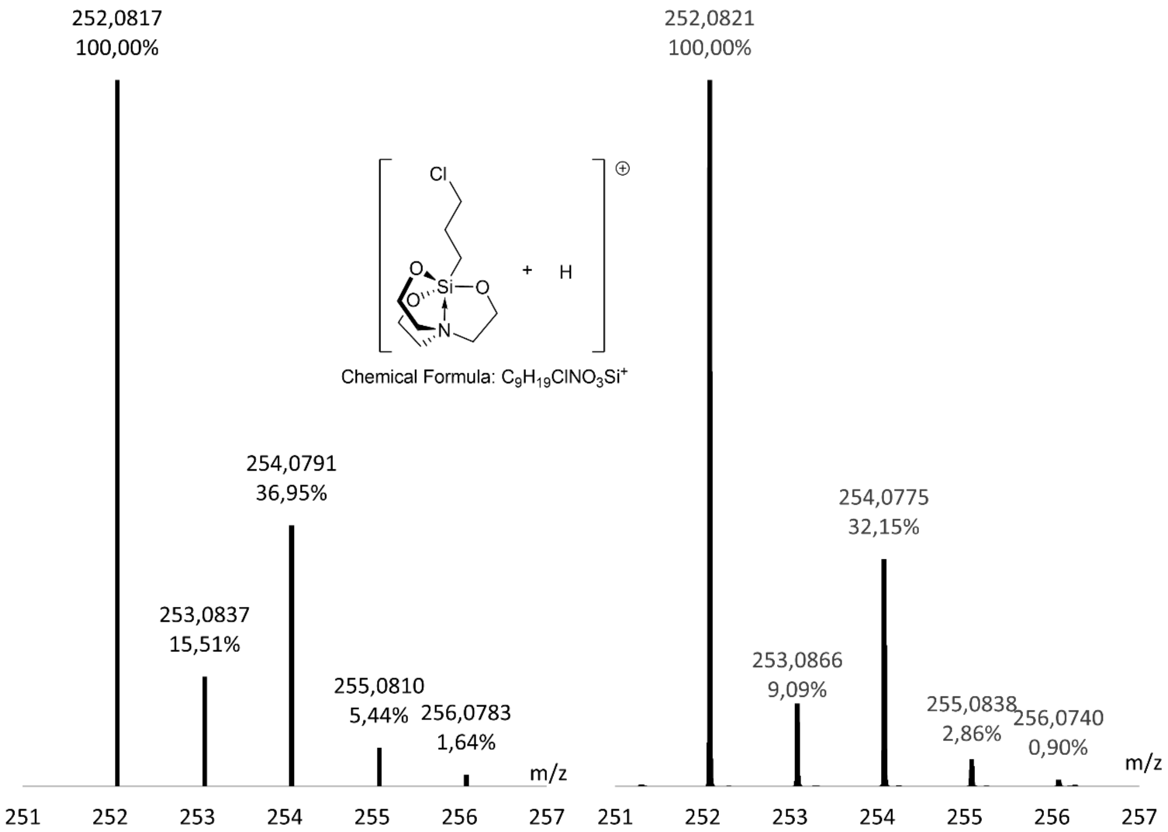

Figure S62. HRMS spectrum of **3b**, predicted (left) and measured (right).

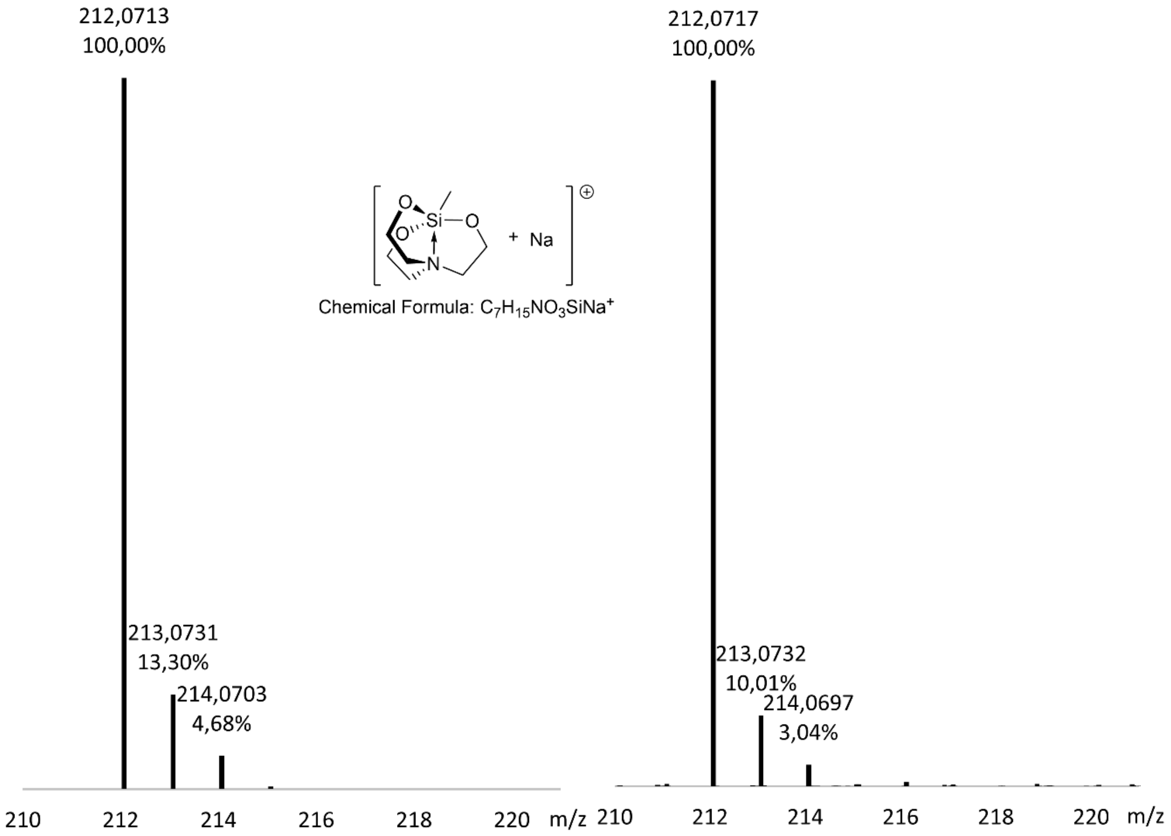

Figure S63. HRMS spectrum of **3c**, predicted (left) and measured (right).

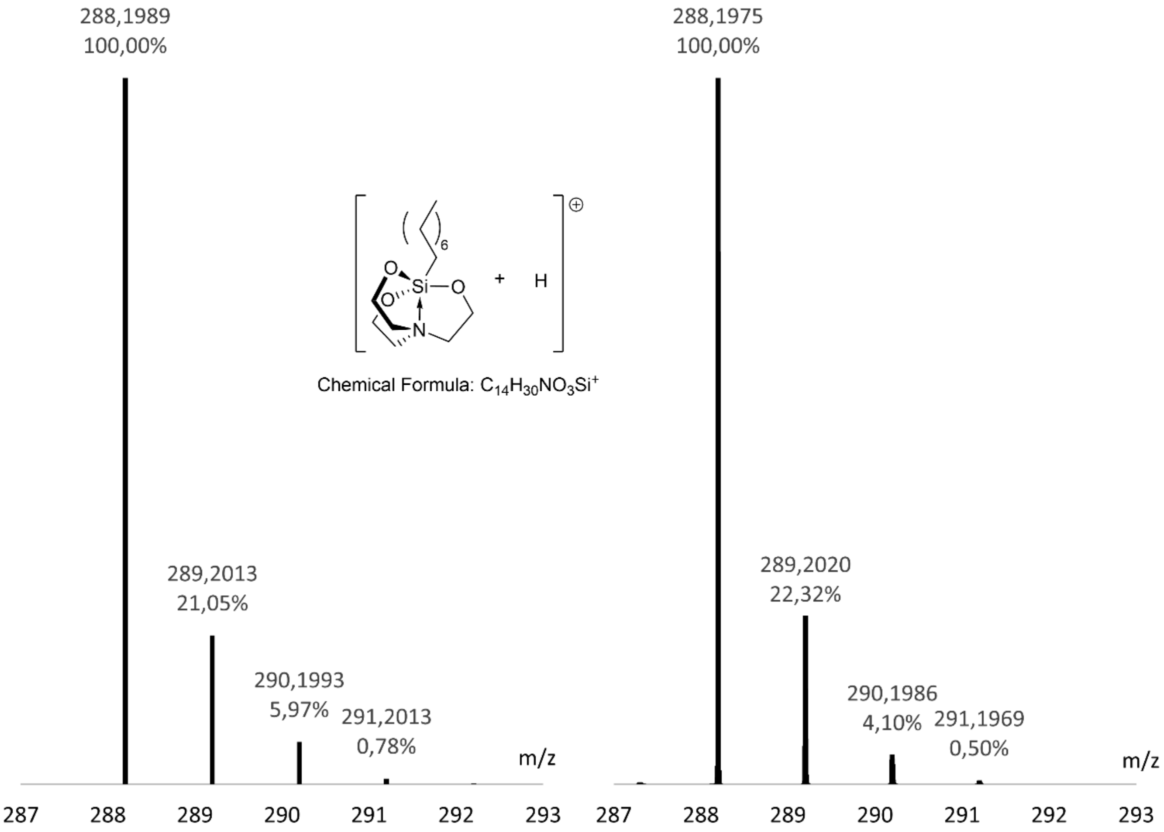

Figure S64. HRMS spectrum of **3d**, predicted (left) and measured (right).

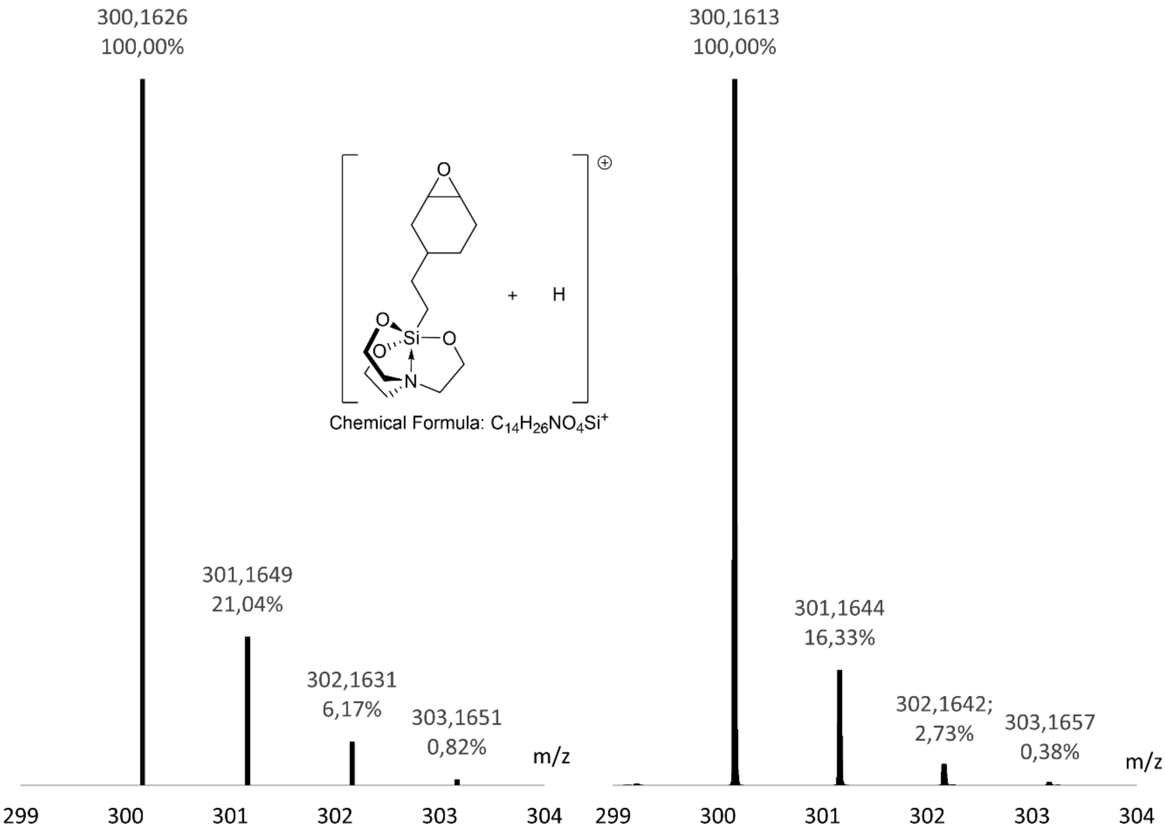

Figure S65. HRMS spectrum of **3e**, predicted (left) and measured (right).

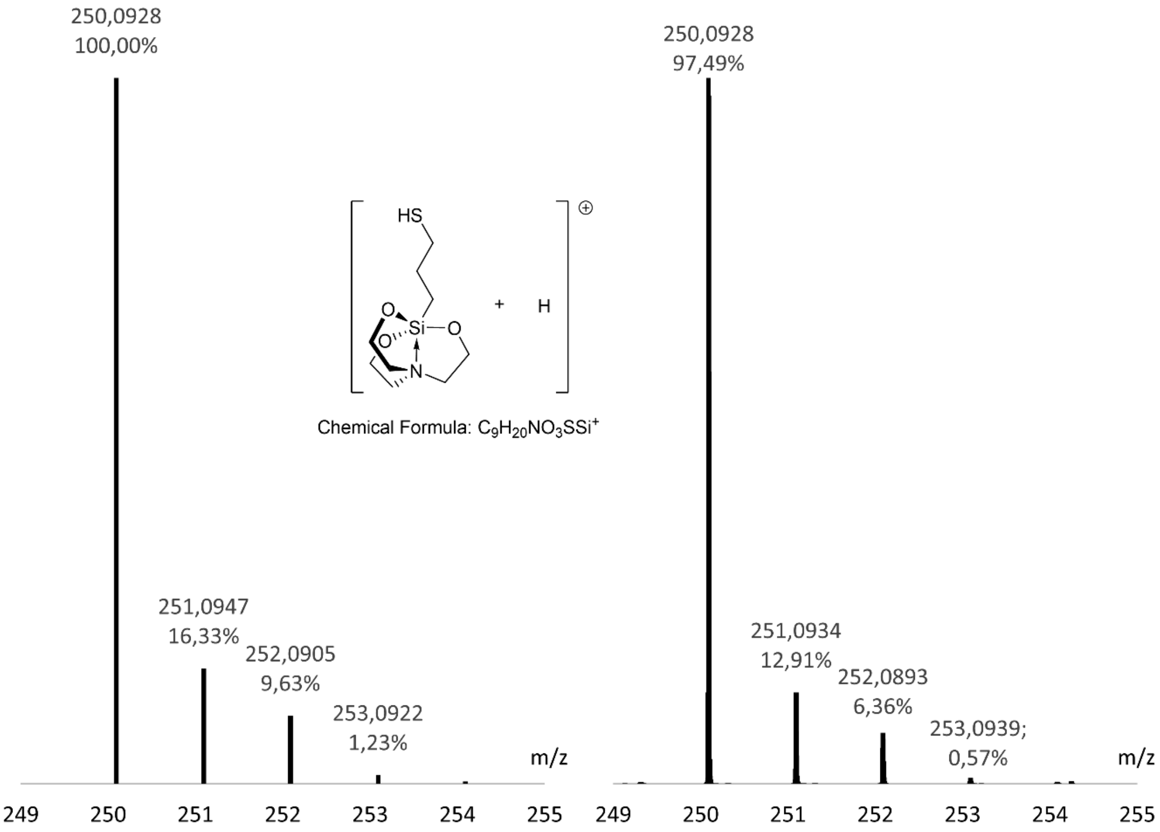

Figure S66. HRMS spectrum of **3f**, predicted (left) and measured (right).

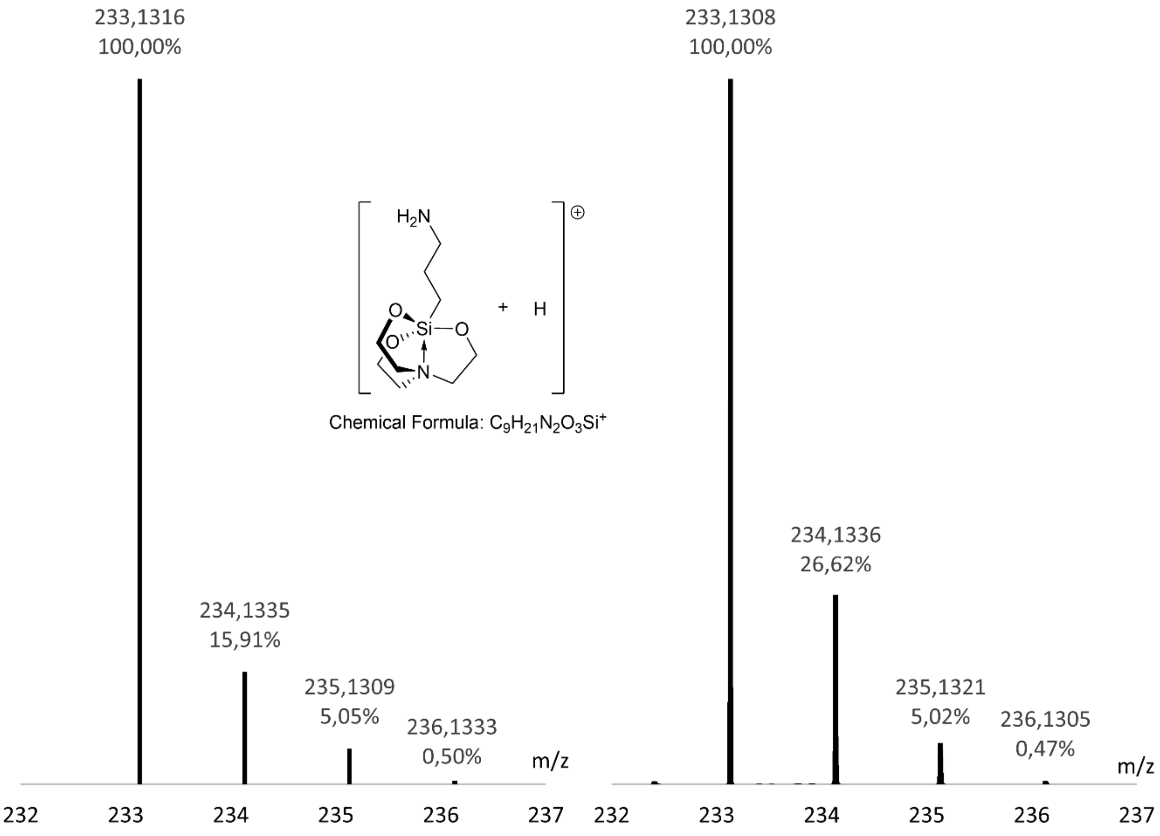

Figure S67. HRMS spectrum of **3g**, predicted (left) and measured (right).

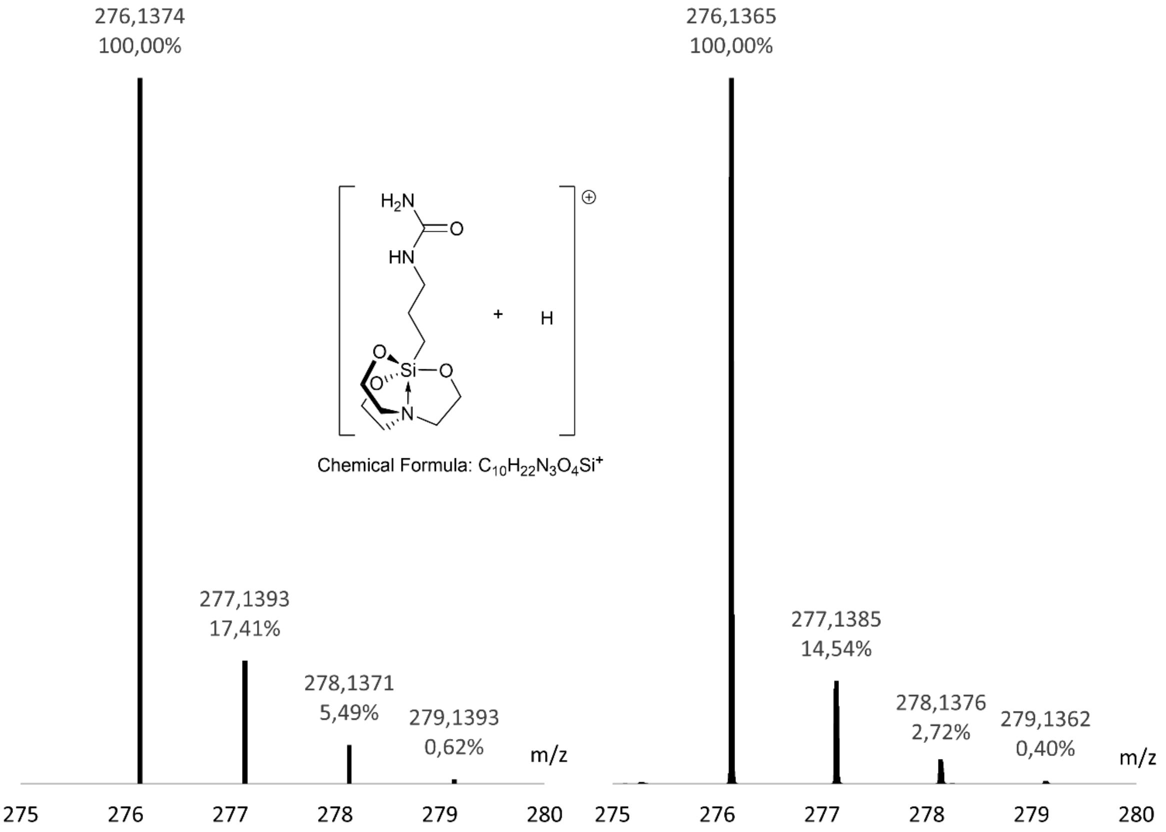

Figure S68. HRMS spectrum of **3h**, predicted (left) and measured (right).

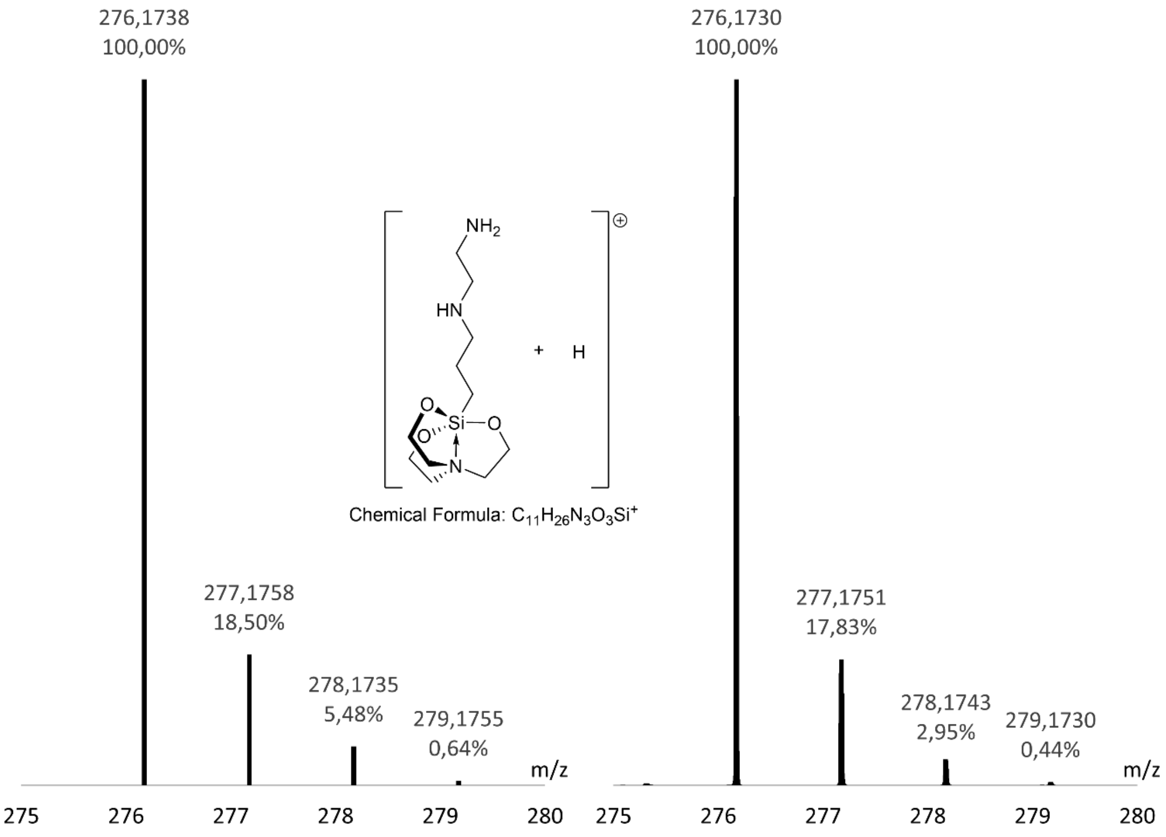

Figure S69. HRMS spectrum of **3i**, predicted (left) and measured (right).

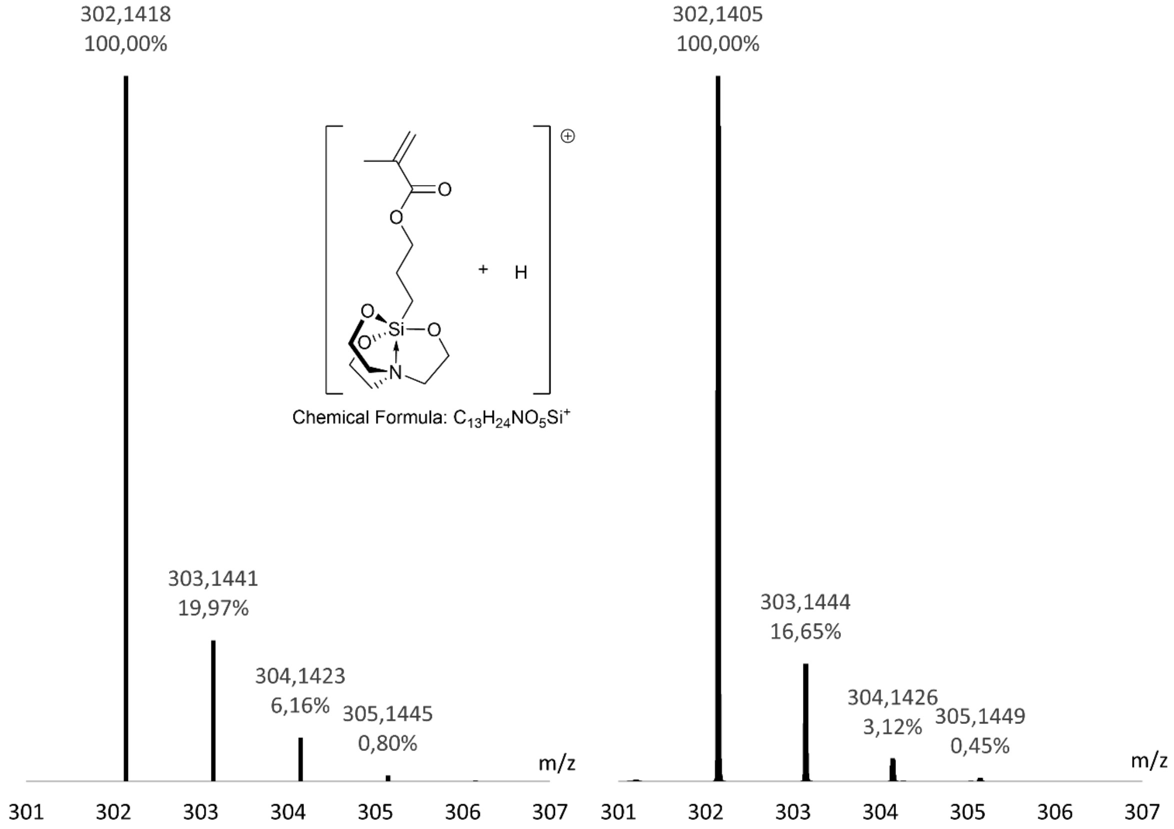

Figure S70. HRMS spectrum of **3j**, predicted (left) and measured (right).

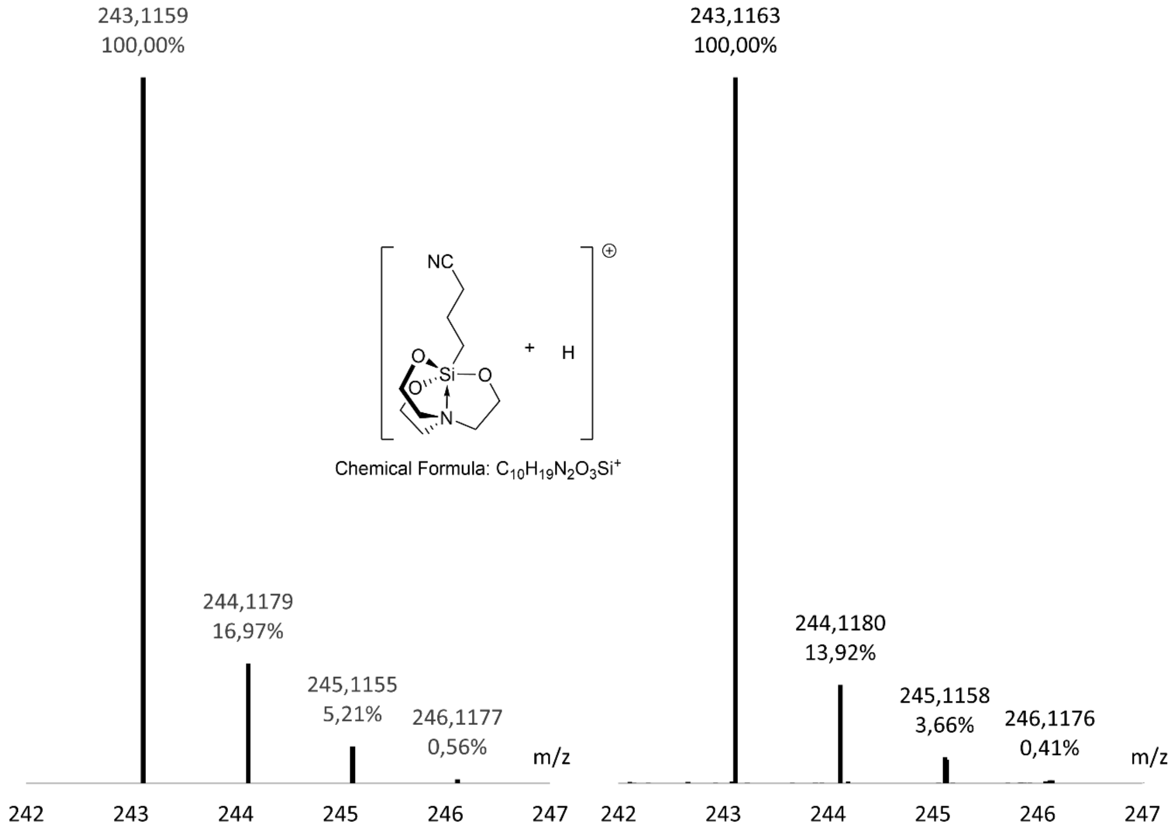

Figure S71. HRMS spectrum of **3k**, predicted (left) and measured (right).

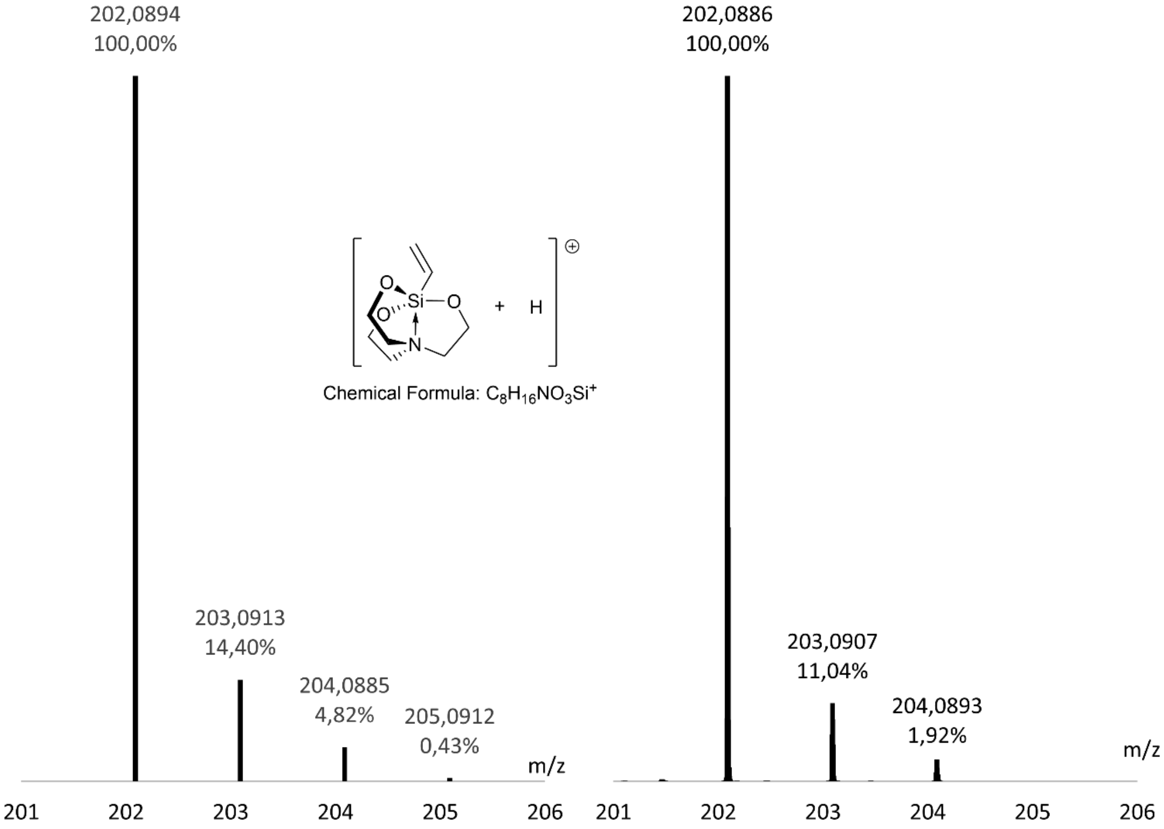

Figure S72. HRMS spectrum of **3l**, predicted (left) and measured (right).

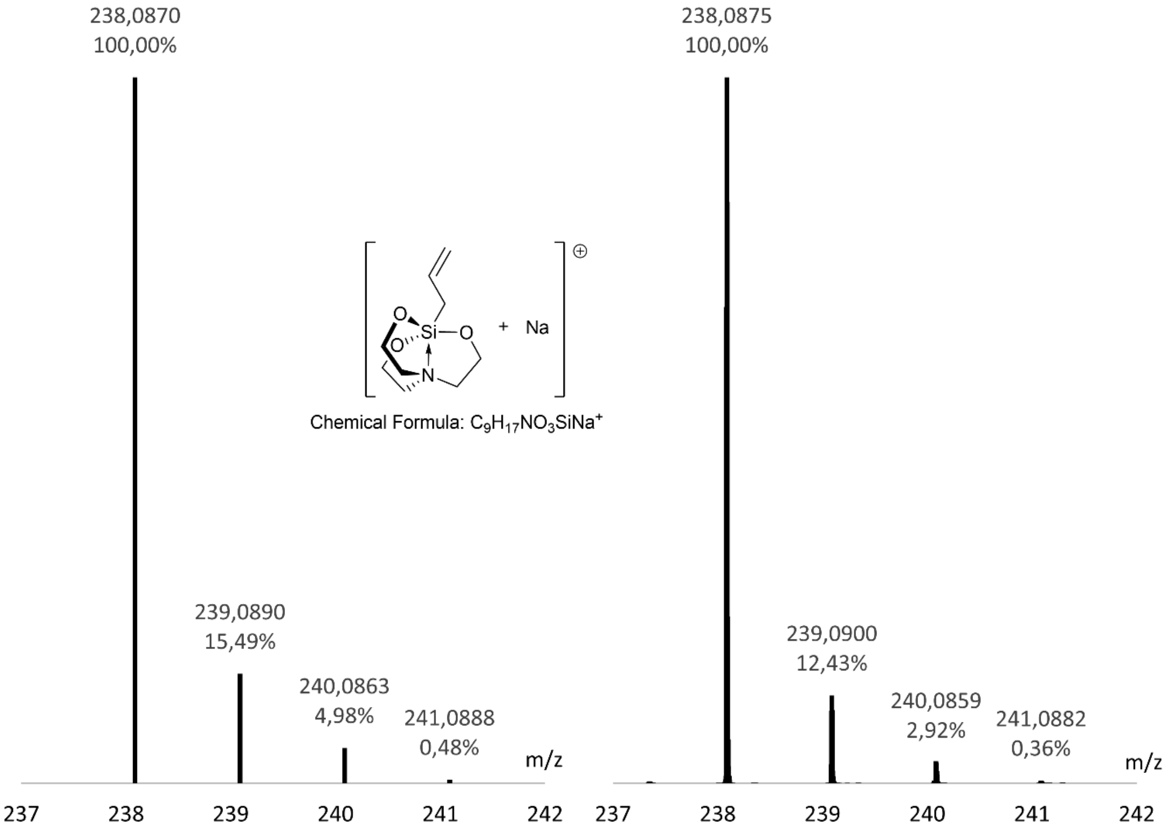

Figure S73. HRMS spectrum of **3m**, predicted (left) and measured (right).

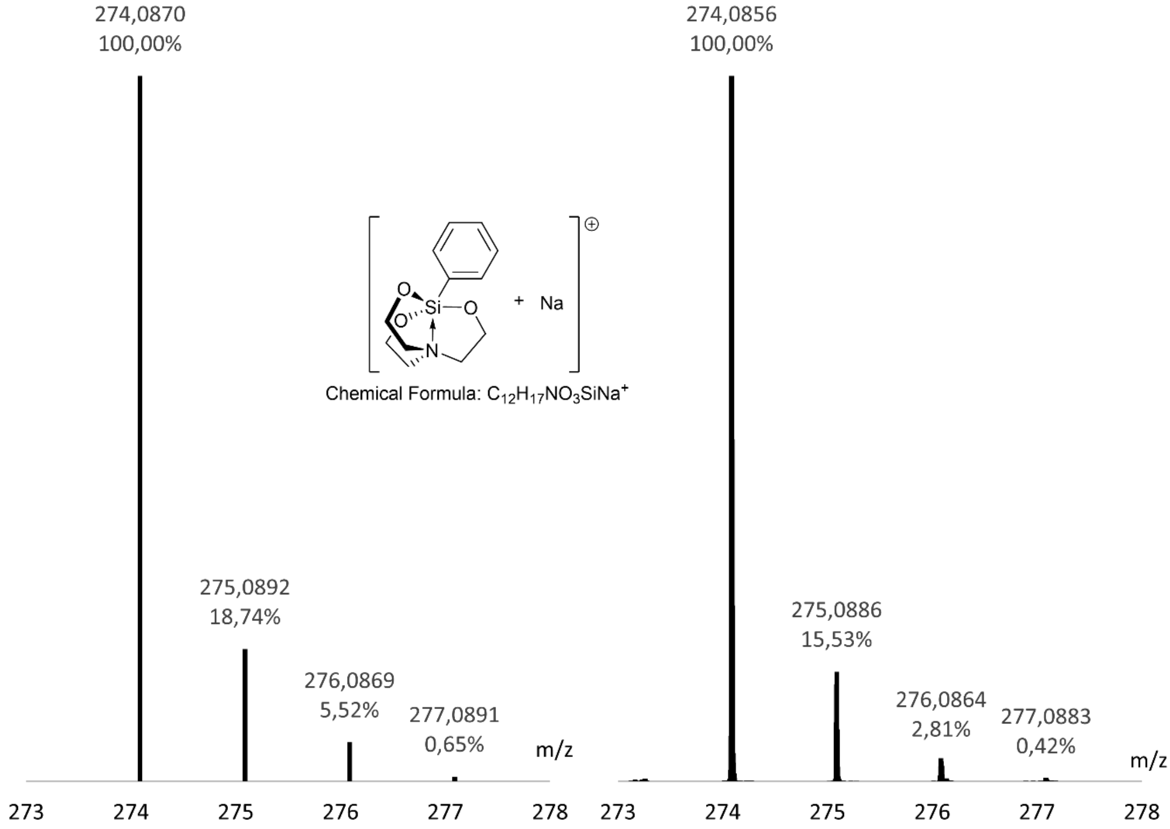

Figure S74. HRMS spectrum of **3n**, predicted (left) and measured (right).

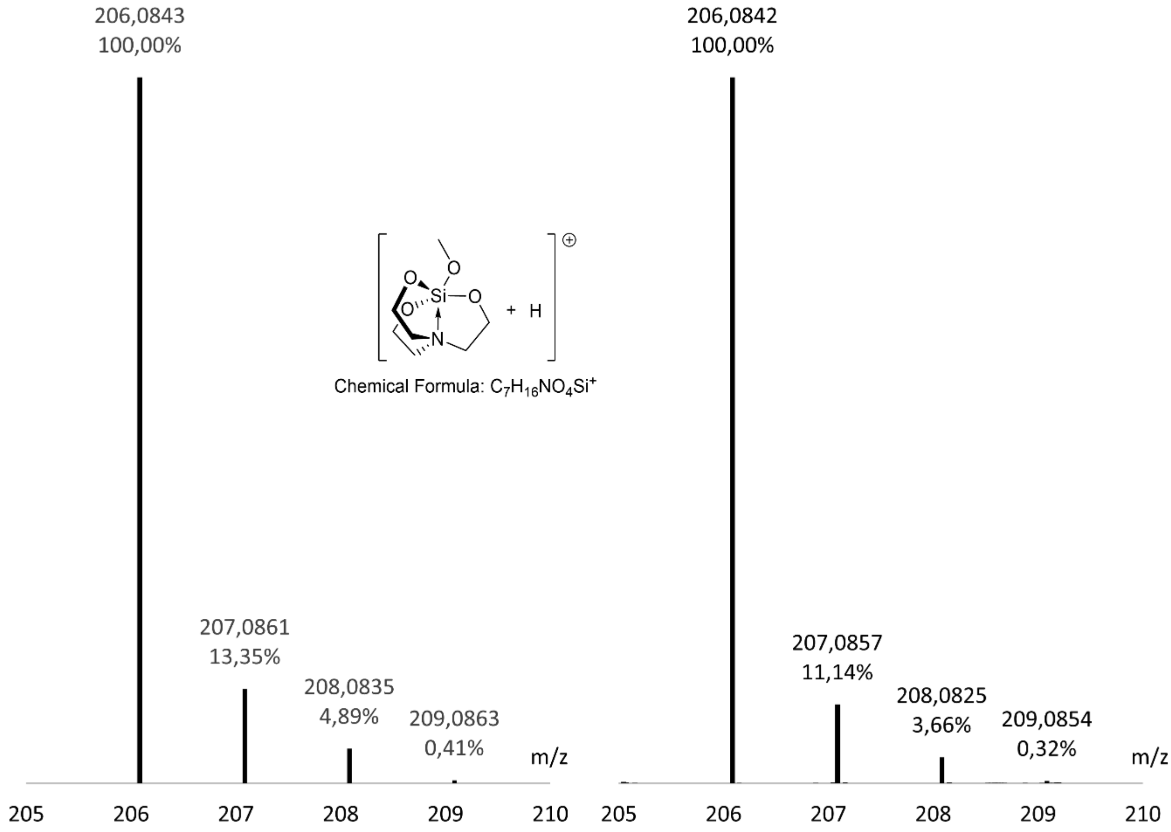

Figure S75. HRMS spectrum of **3o**, predicted (left) and measured (right).

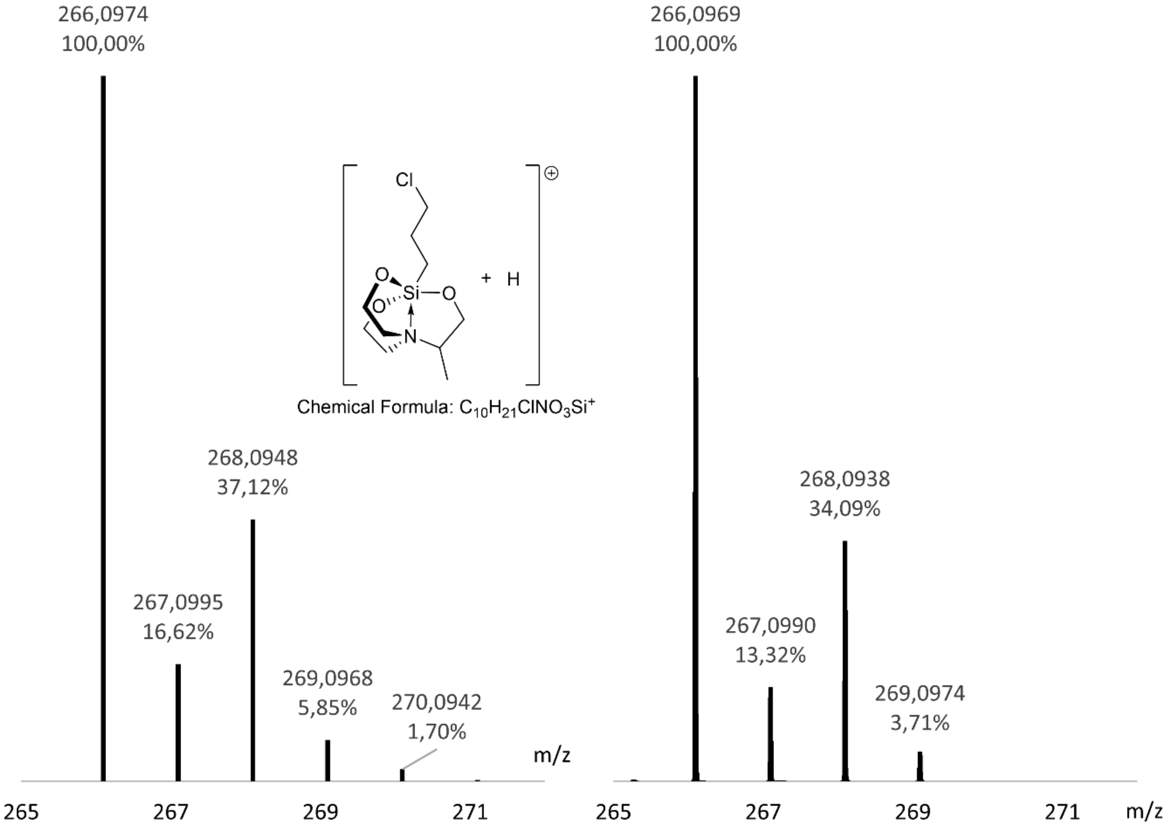

Figure S76. HRMS spectrum of **3p**, predicted (left) and measured (right).

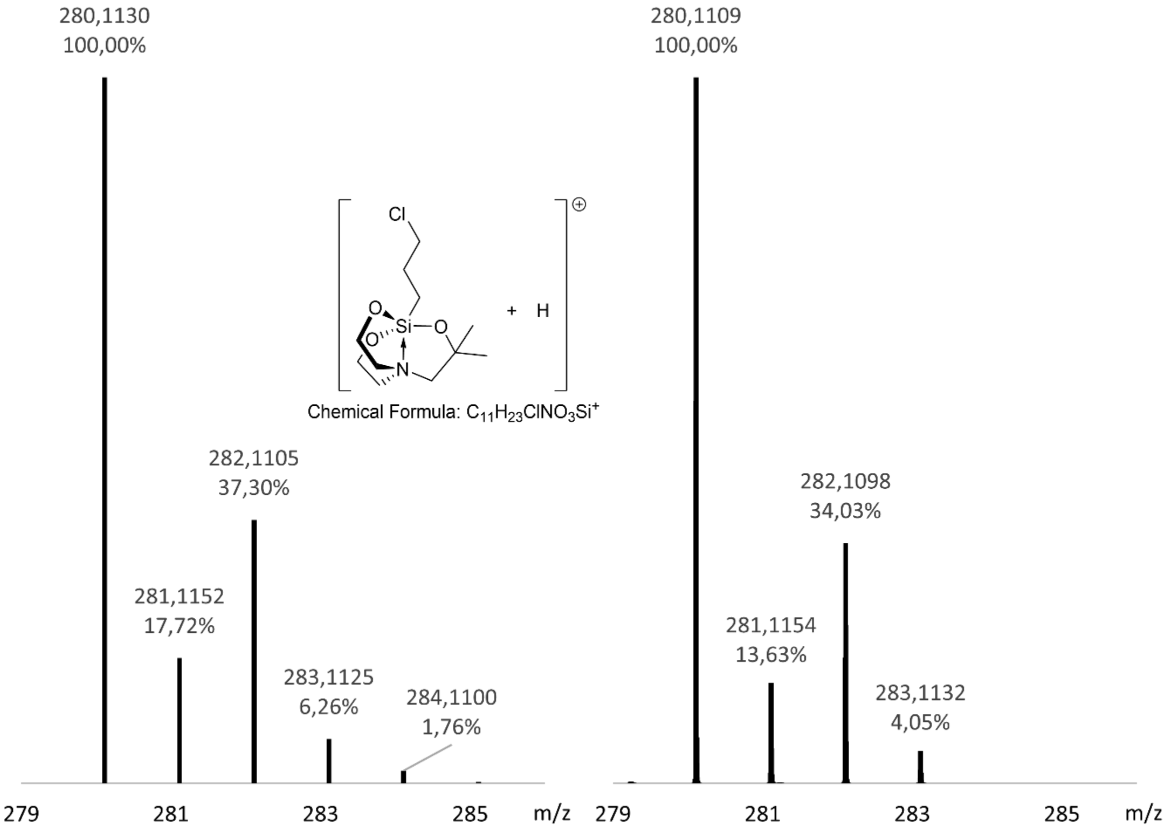

Figure S77. HRMS spectrum of **3q**, predicted (left) and measured (right).

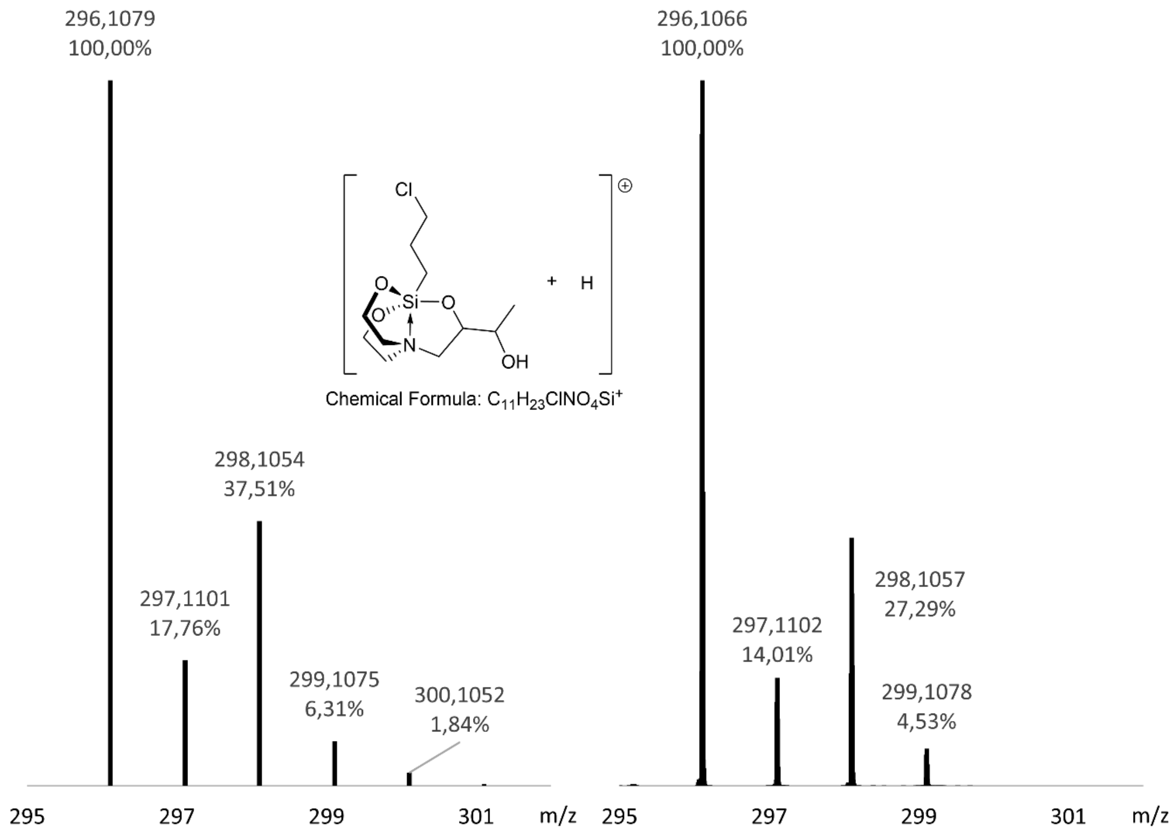

Figure S78. HRMS spectrum of **3r**, predicted (left) and measured (right).

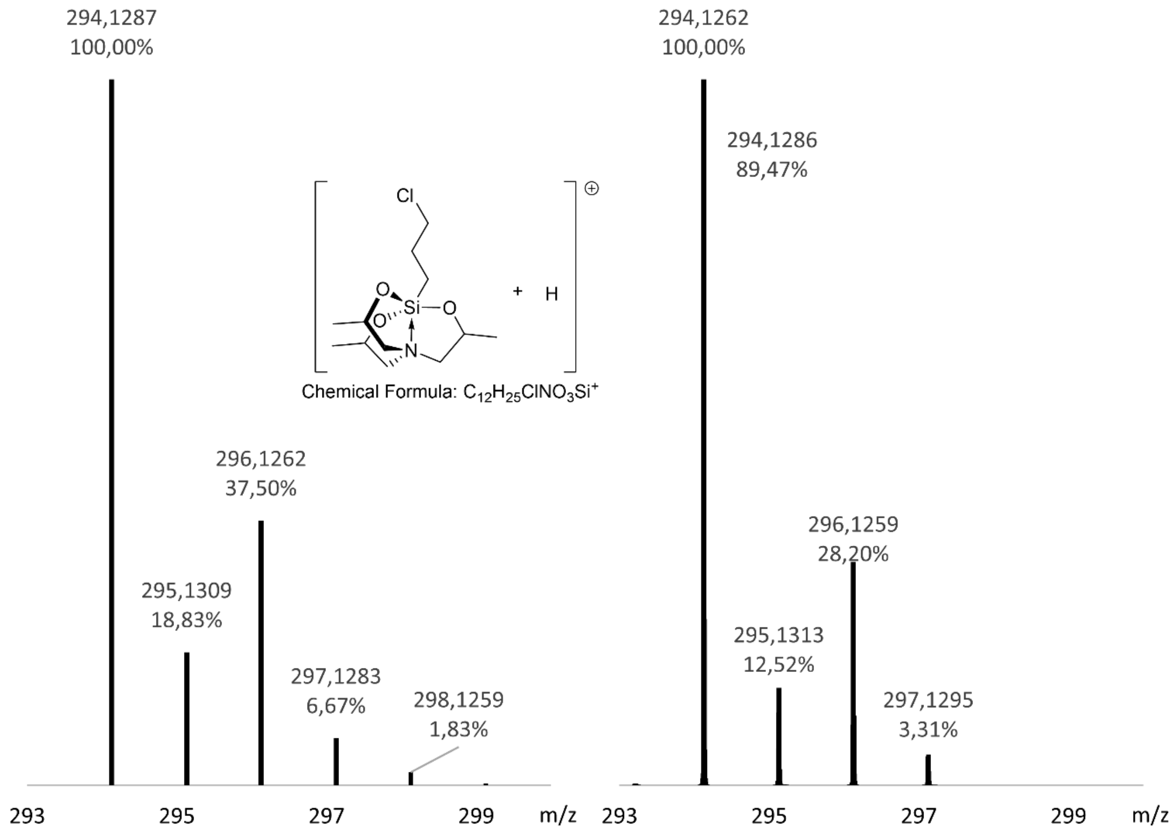

Figure S79. HRMS spectrum of **3s**, predicted (left) and measured (right).

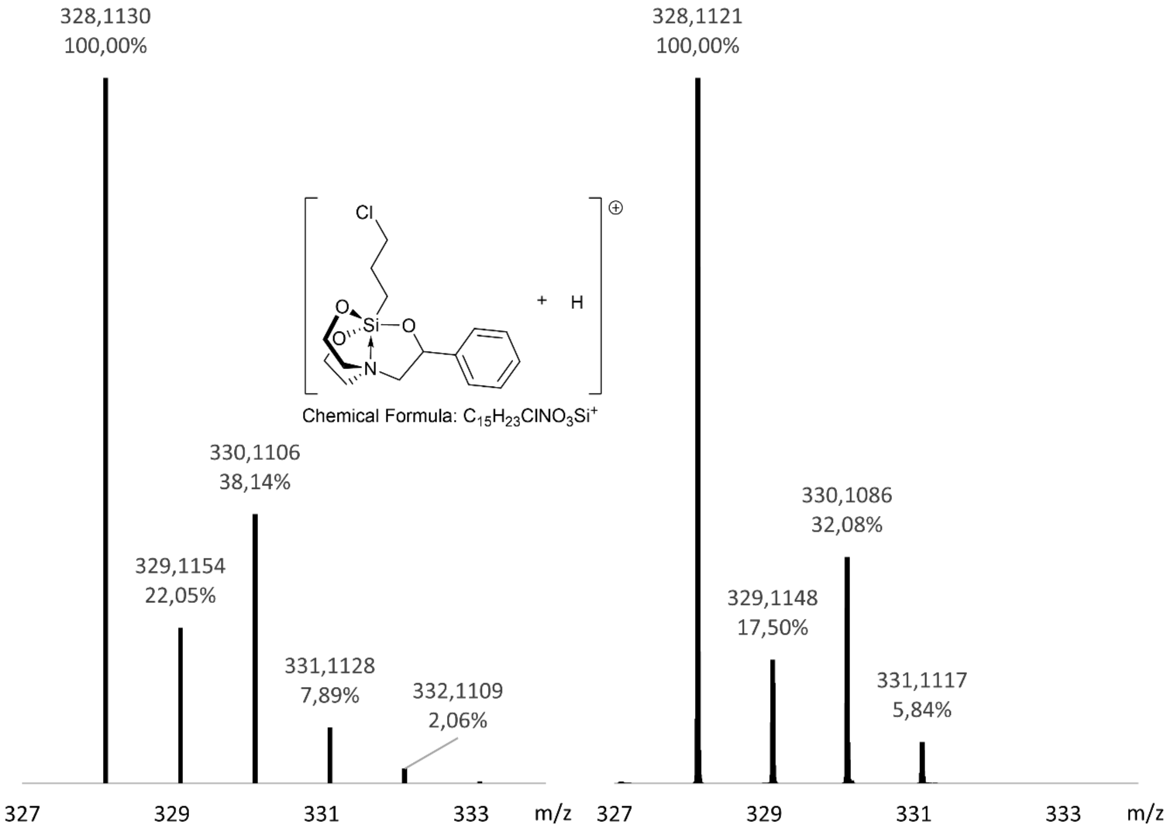

9. Single crystal X-ray crystallography

Diffraction data were collected by the ω-scan technique, using graphite-monochromated MoKα radiation (λ=0.71073 Å), at 100(1) on Rigaku XCalibur four-circle diffractometer with EOS CCD detector. The data were corrected for Lorentz-polarization as well as for absorption effects.[1] Precise unit-cell parameters were determined by a least-squares fit of the reflections of the highest intensity, chosen from the whole experiment. The structures were solved with SHELXT[2] and refined with the full-matrix least-squares procedure on F2 by SHELXL[3]. All non-hydrogen atoms were refined anisotropically. Hydrogen atoms were placed in idealized positions and refined as 'riding model' with isotropic displacement parameters set at 1.2 (1.5 for CH3) times Ueq of appropriate carrier atoms. The crystals of **3s** appeared to be twinned, and this was taken into account during both data reduction and structure refinement. The BASF parameter, describing the content of the two components, refined at 57.12(12)%. In **3c**, the substantial disorder of the ethylene bridges has been found; both alternative positions were however refined without restraints; s.o.f.'s converged at 0.78(1)/0.22(1).

Crystallographic data for the structural analysis have been deposited with the Cambridge Crystallographic Data Centre. Copies of this information may be obtained free of charge from: The Director, CCDC, 12 Union Road, Cambridge, CB2 1EZ, UK; e-mail: deposit@ccdc.cam.ac.uk, or www: www.ccdc.cam.ac.uk.

Table S2. Crystal data, data collection and structure refinement.

Compound	**3c**	**3g**
Formula	$C_{14}H_{29}NO_3Si$	$C_{10}H_{21}N_3O_4Si$
Formula weight	287.47	275.39
Crystal system	triclinic	orthorhombic
Space group	P-1	$P2_12_12_1$
a(Å)	6.8398(6)	7.0939(3)
b(Å)	10.9451(8)	8.8058(4)
c(Å)	11.8810(11)	20.8160(9)

α(º)	114.219(8)	90
β(º)	101.586(7)	90
γ(º)	93.334(6)	90
V(Å^3)	784.73(13)	1300.32(10)
Z	2	4
D_x(g cm^{-3})	1.217	1.407
F(000)	316	592
μ(mm^{-1})	0.154	0.193
Reflections:		
collected	5475	9158
unique (R_{int})	3312 (0.0154)	2646 (0.0376)
with I>2σ(I)	2917	2505
R(F) [I>2σ(I)]	0.0369	0.0333
wR(F^2) [I>2σ(I)]	0.0977	0.0823
R(F) [all data]	0.0438	0.0363
wR(F^2) [all data]	0.1047	0.0842
Goodness of fit	1.081	1.120
max/min Δρ (e·Å^{-3})	0.39/-0.31	0.41/-0.22
CCDC deposition number	2075118	2075117

Compound	**3p**	**3q**	**3s**
Formula	$C_{11}H_{22}ClNO_3Si$	$C_{11}H_{22}ClNO_4Si$	$C_{15}H_{22}ClNO_3Si$
Formula weight	279.83	295.83	327.87
Crystal system	monoclinic	monoclinic	triclinic
Space group	P2$_1$/n	P2$_1$/n	P-1
a(Å)	8.6394(3)	9.0507(3)	7.5396(7)
b(Å)	13.7908(3)	15.9418(5)	11.1082(12)
c(Å)	11.6794(3)	20.3257(8)	20.1465(14)
α(º)	90	90	93.755(7)
β(º)	96.201(3)	98.846(3)	93.076(7)
γ(º)	90	90	108.876(9)
V(Å^3)	1383.39(7)	2897.80(18)	1588.1(3)
Z	4	8	4
D_x(g cm^{-3})	1.344	1.356	1.371
F(000)	600	1264	696
μ(mm^{-1})	0.360	0.353	0.325
Reflections:			
collected	8659	12371	17992
unique (R_{int})	3007 (0.0195)	5801 (0.0274)	10969 (0.0511)
with I>2σ(I)	2649	5168	8752
R(F) [I>2σ(I)]	0.0289	0.0643	0.0569
wR(F^2) [I>2σ(I)]	0.0684	0.1390	0.1567
R(F) [all data]	0.0354	0.0718	0.0696
wR(F^2) [all data]	0.0717	0.1427	0.1631
Goodness of fit	1.042	1.185	1.072
max/min Δρ (e·Å^{-3})	0.41/-0.25	1.03/-0.42	0.48/-0.34
CCDC deposition number	2075115	2075114	2075116

Table S3. Relevant geometrical parameters (Å, °) with s.u.'s in parentheses.

	3c	3g	3p	3q A	3q B	3s A	3s B
Si-O	1.6634(10)	1.6639(19)	1.6591(10)	1.660(2)	1.661(2)	1.666(2)	1.662(2)
	1.6637(10)	1.6766(18)	1.6670(10)	1.664(2)	1.665(2)	1.671(2)	1.667(2)
	1.6747(10)	1.6790(17)	1.6695(10)	1.666(2)	1.666(2)	1.674(2)	1.672(2)
Si1-C1	1.8744(15)	1.886(3)	1.8827(14)	1.879(3)	1.874(3)	1.880(3)	1.885(3)
Si1-N1	2.2086(13)	2.162(2)	2.2094(11)	2.189(3)	2.184(3)	2.149(3)	2.151(3)
O-Si-O	118.36(6)	121.40(10)	118.05(5)	118.38(12)	119.61(13)	119.49(12)	118.72(12)
	118.35(5)	119.36(10)	120.47(5)	118.70(12)	117.33(13)	118.82(12)	120.11(12)
	117.59(5)	115.12(10)	116.50(5)	117.94(12)	118.07(13)	117.59(12)	117.11(12)
O-Si-C	98.19(6)	96.10(10)	96.05(5)	97.20(12)	97.32(13)	96.97(13)	96.59(13)
	99.13(6)	97.39(10)	98.84(6)	97.92(13)	96.69(14)	96.73(13)	97.44(13)
	96.64(6)	96.86(10)	97.55(6)	97.28(13)	98.40(13)	96.58(13)	96.13(13)
O-Si-N	82.16(5)	83.11(9)	82.53(4)	82.27(10)	82.23(11)	83.07(11)	83.18(11)
	82.09(5)	83.18(9)	82.59(4)	82.74(11)	82.39(12)	83.56(11)	83.26(11)
	81.78(5)	83.40(8)	82.50(4)	82.60(11)	83.00(11)	83.09(10)	83.41(10)
C-Si-N	178.34(6)	179.19(11)	178.35(5)	179.30(13)	178.57(13)	179.64(13)	179.28(11)
B/B'			22.7(2))	22.3(3)	25.8(2)	0.4(2)	
Si-O-C-C	27.88(16)	32.2(3)	41.85(15)	34.2(4)	31.8(4)	31.1(4)	30.6(3)
	30.24(19)	34.9(3)	43.56(14)	29.8(4)	31.0(4)	30.9(3)	29.6(3)
	25.83(17)	36.5(3)	31.01(15)	24.5(4)	37.1(4)	29.5(3)	26.0(3)
Si-N-C-C	33.09(13)	31.9(2)	28.50(11)	30.6(3)	33.5(3)	32.8(3)	33.9(3)
	32.17(17)	33.9(2)	28.90(12)	33.8(3)	32.1(3)	33.4(3)	33.9(3)
	32.36(15)	30.3 (2)	24.25(12)	26.9(3)	28.9(3)	33.7(3)	26.0(3)
O-Si-O-C	-82.05(11)	-87.9(2)	-95.69(11)	-86.4(3)	-89.8(3)	-88.0(3)	-87.1(3)
	70.95(12)	68.2(2)	57.12(11)	70.2(3)	64.6(3)	69.1(3)	70.4(3)
	-84.07(12)	-89.5(2)	-98.06(10)	-84.4(3)	-87.4(3)	-86.6(2)	-85.6(2)
	68.72(13)	68.0(2)	58.85(11)	66.8(3)	67.6(3)	70.1(3)	71.1(3)
	-81.39(12)	-93.71(19)	-91.20(11)	-87.9(4)	-89.1(3)	-85.8(3)	-82.4(3)
	71.61(12)	63.3(2)	62.96(12)	68.3(3)	65.5(3)	71.2(3)	74.7(3)
O-C-C-N	-39.38(16)	-40.3(3)	-43.15(14)	-38.6(4)	-44.2(4)	-40.4(3)	-40.9(3)
	-39.9(2)	-43.5(3)	-44.44(14)	-43.2(4)	-40.7(4)	-40.8(3)	-40.5(3)
	-37.70(18)	-41.6(3)	-33.59(14)	-32.5(4)	-37.2(4)	-40.5(3)	-39.5(3)
N-Si-O-C	-5.43(10)	-10.2(2)	-18.97(10)	-10.9(3)	-13.2(3)	-9.0(2)	-8.0(2)
	-7.86(11)	-11.3(2)	-20.30(10)	-8.8(3)	-9.9(3)	-8.5(2)	-7.2(2)
	-4.83(11)	-14.30(18)	-13.75(10)	-7.0(3)	-11.1(3)	-7.5(2)	-4.2(2)

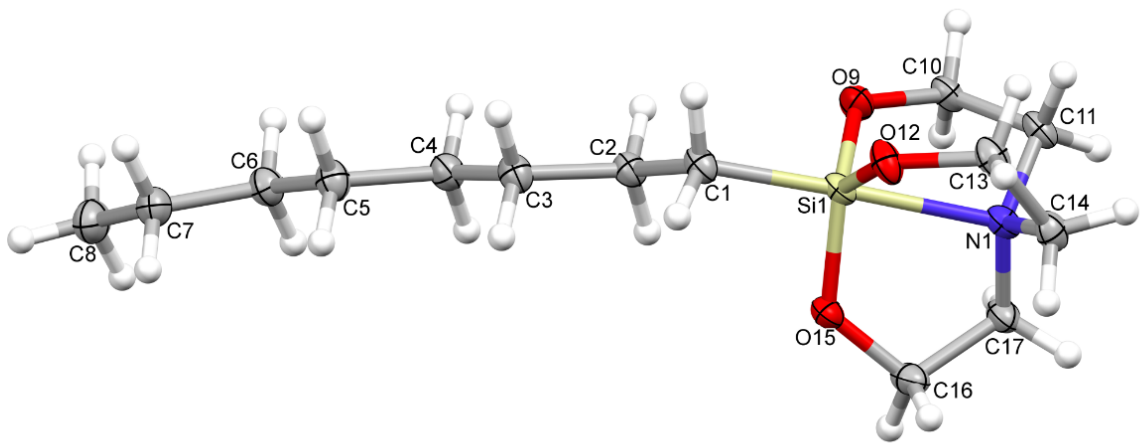

3c

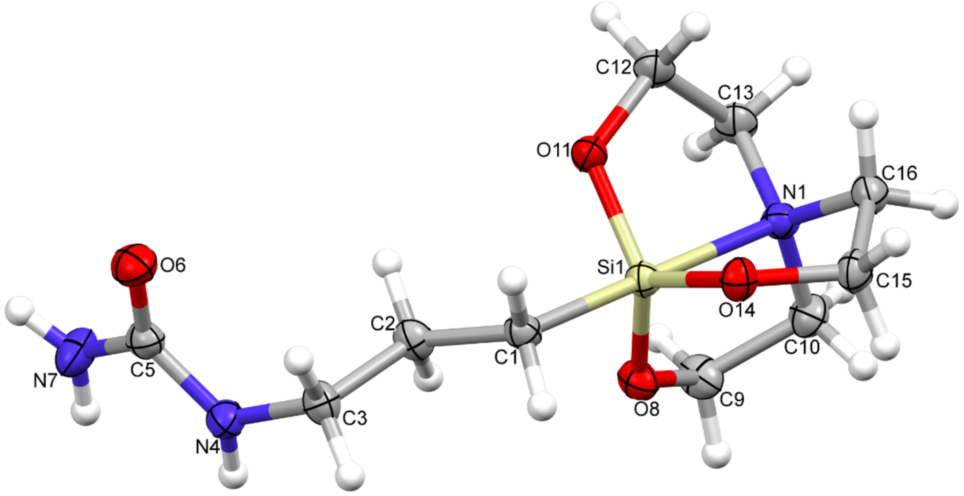
C12
C13
O11
C16
N1
O6
Si1
O14
C15
C2
C10
C1
C5
N7
C9
O8
C3
N4

3g

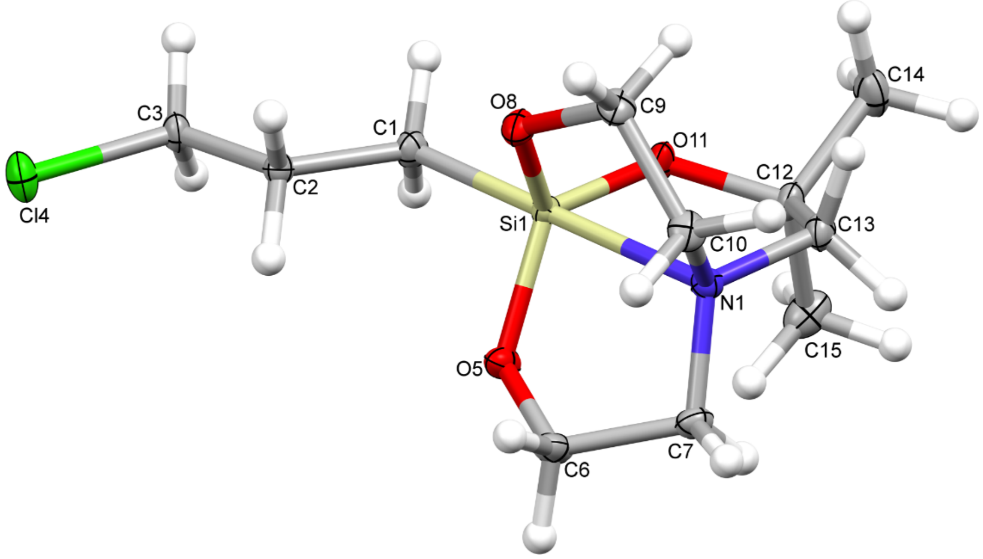
C14
C3
O8
C9
C1
O11
C12
Cl4
C2
Si1
C13
C10
N1
C15
O5
C7
C6

3p

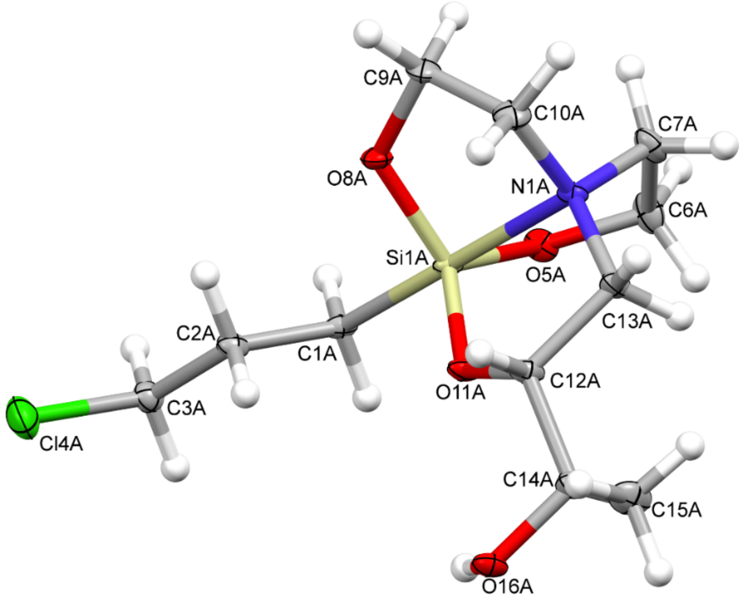
C9A
C10A
C7A
O8A
N1A
C6A
Si1A
O5A
C2A
C13A
C1A
C3A
O11A
C12A
Cl4A
C14A
C15A
O16A

3q

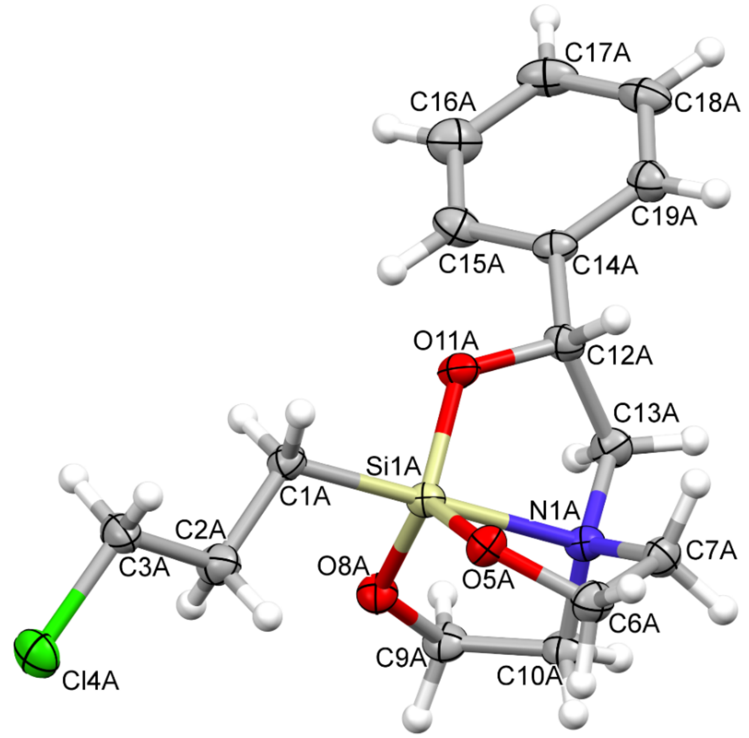

3s

Figure S80. Perspective views of the complexes (one of the symmetry-independent, very similar molecules in cases of **3q** and **3s**). Ellipsoids are drawn at the 50% probability level, hydrogen atoms are shown as spheres of arbitrary radii.

The overall shape of the rigid silatrane cage is very similar in all five compounds (seven molecules, as **3q** and **3s** crystallize with two symmetry-independent molecules in the asymmetric part of the unit cell), as can be seen in Figure S81, which shows the comparison of all molecules. This similarity can be also seen in the values of intra-cage torsion angles, listed in Table S3. The orientation of the substituents differs, depending on the nature of the functional groups and on packing interactions. Besides the structures of **3g** and **3q**, where here are well-defined hydrogen bonds (Figure S82), in all other crystal structures only weak C-H···O and C-H···Cl (in **3q** and **3s**) can be observed. So, in these cases these interactions together with van der Waals forces seem to influence the crystal architectures, which differ form structure to structure.

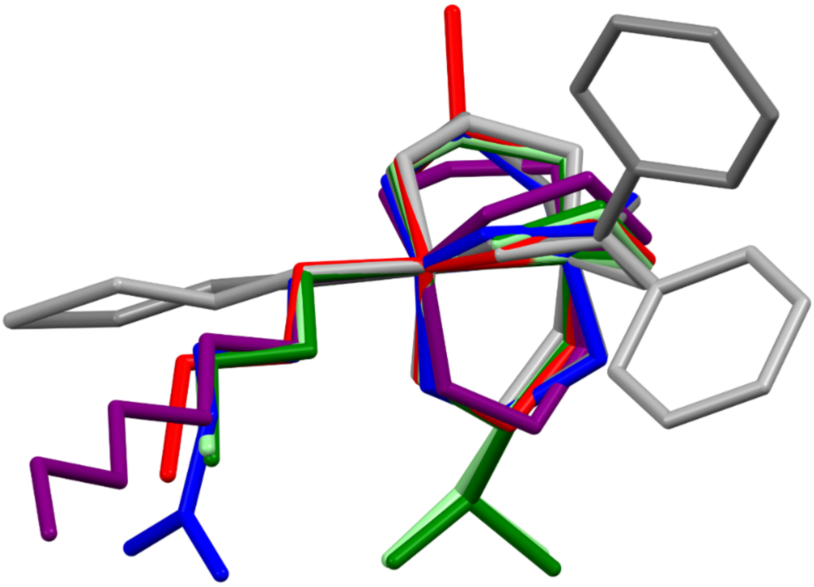

Figure S81. An overlap of all seven molecules found in the crystal structures.

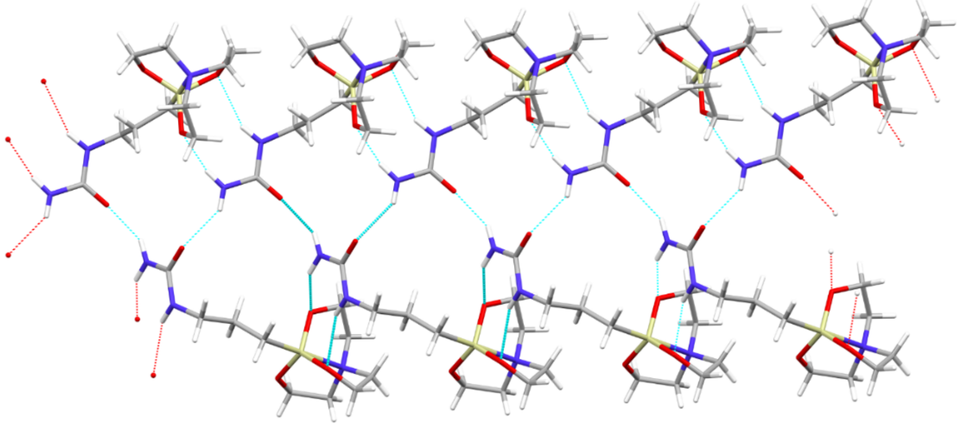

3g

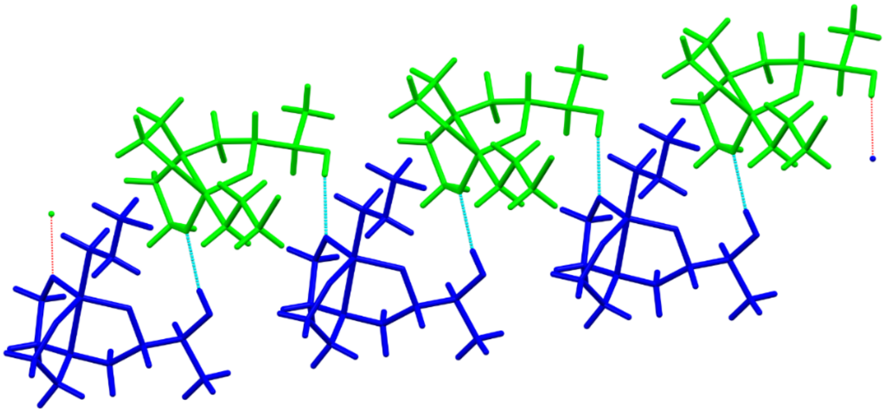

3q

Figure S82. Hydrogen-bond network in the structure of **3g** and **3q** (hydrogen bonds are shown as dashed blue lines); in the case of **3q** the different colours show the different symmetry-independent molecules, so the chain is of ~ABABAB~ composition.

10. References

[1] Rigaku Oxford Diffraction (2015) CrysAlis PRO (Version 1.171.38.41).

[2] G. M. Sheldrick, *Acta Cryst. Part A*, 2015, **71**, 3-8.

[3] G. M. Sheldrick ,*Acta Cryst. Part C*, 2015, **71**, 3-8
